# The Sequence-specific Peptide-binding Activity of the Protein Sulfide Isomerase AGR2 Directs Its Stable Binding to the Oncogenic Receptor EpCAM*[Fn FN2]

**DOI:** 10.1074/mcp.RA118.000573

**Published:** 2018-01-16

**Authors:** M. Aiman Mohtar, Lenka Hernychova, J. Robert O'Neill, Melanie L. Lawrence, Euan Murray, Borek Vojtesek, Ted R. Hupp

**Affiliations:** From the ‡University of Edinburgh, Institute of Genetics and Molecular Medicine, Edinburgh, Scotland, United Kingdom, EH4 2XR;; §National University of Malaysia, UKM Medical Molecular Biology Institute (UMBI), 56000 Kuala Lumpur, Malaysia;; ¶Regional Centre for Applied Molecular Oncology, Masaryk Memorial Cancer Institute, 656 53 Brno, Czech Republic;; ‖University of Gdansk, International Centre for Cancer Vaccine Science, ul. Wita Stwosza 63, 80–308 Gdansk, Poland

## Abstract

AGR2 is an oncogenic endoplasmic reticulum (ER)-resident protein disulfide isomerase. AGR2 protein has a relatively unique property for a chaperone in that it can bind sequence-specifically to a specific peptide motif (TTIYY). A synthetic TTIYY-containing peptide column was used to affinity-purify AGR2 from crude lysates highlighting peptide selectivity in complex mixtures. Hydrogen-deuterium exchange mass spectrometry localized the dominant region in AGR2 that interacts with the TTIYY peptide to within a structural loop from amino acids 131–135 (VDPSL). A peptide binding site consensus of Tx[IL][YF][YF] was developed for AGR2 by measuring its activity against a mutant peptide library. Screening the human proteome for proteins harboring this motif revealed an enrichment in transmembrane proteins and we focused on validating EpCAM as a potential AGR2-interacting protein. AGR2 and EpCAM proteins formed a dose-dependent protein-protein interaction *in vitro*. Proximity ligation assays demonstrated that endogenous AGR2 and EpCAM protein associate in cells. Introducing a single alanine mutation in EpCAM at Tyr251 attenuated its binding to AGR2 *in vitro* and in cells. Hydrogen-deuterium exchange mass spectrometry was used to identify a stable binding site for AGR2 on EpCAM, adjacent to the TLIYY motif and surrounding EpCAM's detergent binding site. These data define a dominant site on AGR2 that mediates its specific peptide-binding function. EpCAM forms a model client protein for AGR2 to study how an ER-resident chaperone can dock specifically to a peptide motif and regulate the trafficking a protein destined for the secretory pathway.

Anterior Gradient-2 (AGR2)^1^ is an endoplasmic reticulum (ER) localized protein disulfide isomerase superfamily member (1) that is up-regulated in a large number of human cancers (2). Three biological paradigms have emerged from studies on AGR2. The first paradigm holds that the normal cell adhesion associated function of AGR2 is exploited as an oncogenic signal in cancer development. This concept was developed based on data demonstrating that AGR2 protein is required to assemble the dorso-anterior ectoderm that forms the cement gland in vertebrates thus maintaining forebrain integrity (3, 4). The cement gland mediates the attachment of the growing epithelium to a solid support (5). Subsequent data highlighting a role for AGR2 in mammalian cancer-associated cell adhesion (6) (7) provided the link between the normal developmental function of AGR2 and its oncogenic activity.

The second paradigm maintains that the normal cell migration-promoting function of AGR2 that mediates the regeneration of limb of amphibian (8) is exploited as an oncogenic signal during cancer progression. Consistent with this data, recent studies have also highlighted that topical application of AGR2 protein can accelerate wound-healing in mammalian models (9). Finally, studies in transgenic mice have shown that AGR2-null animals are defective in mucin production, have alterations in asthma incidence (10), and are primed to develop inflammatory bowel disease (11). This third paradigm, therefore, claims that the ability of AGR2 to mediate oncogenic growth is linked to its ability to catalyze the maturation of cysteine-rich receptors that play cancer associated functions *in vivo*. Recent data has further highlighted an extracellular role for AGR2 in promoting cancer growth and complex organoid structures (12). Thus, the three paradigms derived from normal developmental biology have suggested that the cancer-associated function for AGR2 derives from its ability to promote cell adhesion, stimulate cell migration through an extra-cellular activity, and catalyze plasma membrane receptor trafficking through an intra-cellular function. The biochemical mechanisms underlying how AGR2 mediates these three biological pathways are not fully defined.

The ER organelle is a site of protein folding and assembly. Thioredoxin-type molecular chaperones like AGR2 presumably enable the folding and trafficking of cysteine-rich transmembrane receptors with complex protein folding requirements. Classic thioredoxins have a conserved thioredoxin fold comprised of the CxxC motif that mediates covalent bond formation with cysteine-containing client proteins followed by resolution through cycles of reduction-oxidation (13). AGR2, by contrast, is part of the thioredoxin superfamily that contain CxxS motifs and which lack the ability to exploit a two-cysteine redox system that classically mediates client protein oxidation and reduction cycles (14). Through this single cysteine, AGR2 can mediate the maturation of receptors including MUC5 family and MUC2 (10) (15). Trafficking of EGFR to the plasma membrane through AGR2-dependent catalysis in cancers might also be important for its' oncogenic function (16) because AGR2-negative cells fail to localize EGFR to its membrane destination. Presumably, AGR2 interacts with EGFR in the ER and facilitates its' folding and maturation, although there is no evidence for this. There might also be trafficking-independent functions for AGR2 in cancer. AGR2 overexpression in isogenic cancer cells can upregulate the proliferation biomarker Ki67 and the tumor modifier TSG101 resulting in p53 protein degradation (17) and AGR2 has extra-cellular oncogenic roles independent of its intra-cellular ER secretory activity (12).

In addition to its classic thioredoxin fold, additional structure-function features of AGR2 protein are emerging. In addition to its N-terminal ER-leader sequence, and degenerated C-terminal ER-retention site (KTEL), there are additional peptide motifs that impact on its protein-protein interactions. The protein can exist as a homodimer through protein-protein contacts stabilized by an EALYK motif from amino acids 60–64 (7). An N-terminal stretch of amino acids from 21–40 are intrinsically disordered and negative regulatory dimer stability; it is striking that deletion of the N-terminal 40 amino acids of AGR2 can increase dimer stability by three orders of magnitude (7). These data indicate that the full-length AGR2 might have a propensity to exist in a monomeric rather than a fully dimeric state. The function of this weak dimeric structure of AGR2 is becoming apparent. The monomeric E60A mutant of AGR2 is fully active in cell-adhesion activity, whereas deletion of amino acids 21–40 that removes the intrinsically disordered domain attenuates its migration function(7). These data together suggest that monomeric form of AGR2 is responsible for its' cell adhesion functions.

There are very few well-validated binding proteins for AGR2 that explain its oncogenic activity. One yeast two-hybrid screen identified the oncogenic membrane receptor Dystroglycan, although this protein-protein interaction is largely unvalidated (18). Nevertheless, antibodies to Dystroglycan or AGR2 can inhibit cancer cell growth (19). Another yeast-two hybrid screen has identified a set of AGR2-interacting proteins that provide clues into its intracellular functions (20). One of the most well-validated of these protein-protein interactions is with the hexameric molecular chaperone and AAA+ superfamily protein Reptin (21). Reptin binds to a divergent hydrophobic peptide motif on AGR2 (amino acids 104–111) (20). New drug leads that bind to the Walker-A ATP-binding pocket of Reptin can also stimulate Reptin binding to AGR2 (22). Deletion of the N-terminal domain of AGR2 (*e.g.* stabilizing the dimeric form) can stimulate binding of AGR2 to Reptin (23). These data together suggest that the monomeric and dimeric forms of AGR2 can have distinct functions. Whether Reptin and AGR2 cooperate in protein-folding pathways remains unknown.

Molecular chaperones and protein disulfide isomerases are generally thought to interact non-specifically with hydrophobic polypeptide regions or cysteine residues, respectively. Accordingly, then, perhaps the most striking feature of AGR2 protein is its ability to bind to peptides in a sequence-specific manner. The AGR2 protein was screened for peptide binding aptamers using peptide-phage libraries resulting in the acquisition of two types of peptides that bind to different domains on the protein (24). However, the function of this sequence-specific peptide binding function of AGR2 has not been defined. In the case of perhaps the most well-characterized specific peptide-binding protein, MDM2 (25), the function of this peptide binding motif is to drive selective interaction with several interacting proteins in cells (26).

In this report, we probed this specific peptide binding function of AGR2 to define a possible biological function for this activity. For example, the interaction site could form a docking site for client proteins that enter the ER or it could simply be involved in trafficking AGR2 through adaptor proteins. Hydrogen-deuterium exchange mass spectrometry was first applied to determine whether a specific peptide-docking site could be mapped on AGR2. Subsequently, an optimized consensus site for AGR2 binding peptides was used to search for human proteins with this consensus motif. An enrichment of transmembrane proteins containing the motif was identified suggesting that a major role for this motif could be in binding to client proteins destined for membrane localizations. A specific protein-protein interaction with the oncogenic membrane receptor EpCAM was validated. This suggests a mechanism for how this docking site on EpCAM can impact on the AGR2-EpCAM protein-protein interaction. These data have implications for how this ER-resident chaperone mediates maturation of proteins destined for the plasma membrane. During these studies, a clinical proteomics analysis using resected biopsies from patients with esophageal adenocarcinoma identified AGR2 and EpCAM as highly expressed in primary adenocarcinoma as well as cancer-associated lymph nodes (27). This allows scientific information acquired on AGR2 and EpCAM pathway regulation to be translated into novel therapeutics that target the AGR2:EpCAM axis for improved cancer management.

## EXPERIMENTAL PROCEDURES

### 

#### 

##### Chemicals, Antibodies, and Peptides

All chemicals and solvents were obtained from commercial sources and of high purity or HPLC spectral grade. AGR2 polyclonal antibody K47 (Moravian-Biotechnology, Brno, Czech Republic) was used at 1:1000 dilution, EpCAM monoclonal antibody (VU-1D9, Calbiochem) was used at 1:1000, mCherry monoclonal antibody (ab125096, Abcam) was used at 1:1000, and GFP polyclonal antibody (A-11122, Thermo Fischer Scientific) was used at 1:1000. Secondary antibodies were Horseradish-peroxidase (HRP) conjugated anti-Rabbit IgG (P0217, Dako) at 1:1000 and HRP-conjugated anti-Mouse IgG (P0260, Dako) at 1:1000. Peptides were from Chiron Mimotopes, Melbourne, Australia.

##### Bacterial Strains and Growth Conditions

The bacterial strains and plasmids used in this work are described in Table I*A* and *B. E. coli* strains harboring plasmids were grown aerobically at 37 °C and 200 rpm in Luria-Bertani (LB) broth medium, supplemented with ampicillin (100 μg/ml).

**Table I:** Strains and plasmids used in this work

Plasmid Name	Description	Reference
A. Bacteria *Escherichia coli*		
DH5α	F– Φ80*lac*ZΔM15 Δ(*lac*ZYA-*arg*F) U169 *rec*A1 *end*A1 *hsd*R17 (rK–, mK+) *pho*A *sup*E44 λ– *thi*-1 *gyr*A96 *rel*A1	Thermo Fisher Scientific
BL21-DE(3)	fhuA2 [lon] ompT gal (λ DE3) [dcm] ΔhsdS λ DE3 = λ sBamHIo ΔEcoRI-B int::(lacI::PlacUV5::T7 gene1) i21 Δnin5	Thermo Fisher Scientific
BL21-AI	F– ompT hsdSB(rB–, mB–) gal dcm araB::T7RNAP-tetA	Thermo Fisher Scientific
B. Plasmids		
pmAGR2	Mature AGR2 (amino acids 21–175). N-term 6xHis-tag. Amp^R^	This work
pEpEX-CO	Human codon optimized extracellular domain of EpCAM (amino acids 24–265). N-Term 6xHis-tag. Amp^R^	Thermo Fisher Scientific
pmAGR2D132A	Asp132 of pmAGR2 mutated to Ala. Amp^R^	This work
pmAGR2P133A	Pro133 of pmAGR2 mutated to Ala. Amp^R^	This work
pmAGR2S134A	Ser133 of pmAGR2 mutated to Ala. Amp^R^	This work
pEpEXY251A-CO	Tyrosine251 of pEpEX-CO mutated to Ala. Amp^R^	This work
pAGR2-C-mCherry	Full-length AGR2 cloned into pcDNA3/GW-Cherry. Amp^R^	This work
pEpCAM-C-EGFP	Full-length EpCAM cloned into pEGFP-N1. Kan^R^	This work
pEpCAM-Y251A-C-eGFP	Tyrosine251 of pEpCAM-C-eGFP mutated to Ala. Amp^R^	This work

##### Plasmids

The AGR2 recombinant plasmid (pmAGR2) encoding the mature version of the protein was described previously (24). AGR2 was previously cloned into pDEST17 using Gateway cloning system (Invitrogen). The plasmid pmAGR2 contains an in-frame 6× Histidine (His) epitope tag fused to the N terminus of AGR2 (amino acid 21–175). EpEX (amino acid 24–265 of accession number NM_002354) was chemically synthesized and human codon optimized (GeneArt Gene Synthesis, ThermoFisher Scientific) and cloned into pET151/d-TOPO harboring 6x His epitope tag followed by TEV protease site fused to N terminus of EpEX. Mutants His-AGR2 and His-EpCAM were made according to the protocol described in QuickChange site-directed mutagenesis (Agilent Technologies) using High Fidelity PCR, 2x *Pfu* Turbo master mix (Rovalab). AGR2 mutants pmAGR2D132A (containing Asp132 to Ala), pmAGR2P133A (containing Pro133 to Ala), pmAGR2S134A (containing Ser134 to Ala) were generated using AGR2-D132A-F and AGR2-D132-R, AGR2-P133A-F and AGR2-P133A-R, and AGR2-S134A-F and AGR2-S134A-R mutagenic primers respectively as described in Table II using pmAGR2 as a template. EpCAM mutants pEpEX-Y251A-CO and pEpCAM-Y251A-C-EGFP (containing Tyrosine251 to Ala) were generated using p-Y251A-F and pEpEX-Y251A-R and pEpCAMY251A-C-EGFP-F and pEpCAM-Y251A-C-EGFP-R mutagenic primers respectively as described in Table II. Full-length AGR2 was previously cloned into pcDNA3/GW-Cherry(28). This construct harbors C-terminally tagged mCherry. Full-length EpCAM was PCR amplified using forward primer EpCAM-C-EGFP-F and reverse primer EpCAM-C-EGFP-R (Table II) that contains XhoI and *Age*I restriction sites at N and C terminus respectively of FL-EpCAM and cloned into multiple cloning sites of pEGFP-N1 using the same restriction sites. FL-EpCAM from this construct harbors C-terminally tagged EGFP. All constructs were confirmed by DNA sequencing and analyzed using SnapGene v3.0.

**Table 2:** Nucleotide sequences of the oligonucleotide primers used in this study

Primer Names	Plasmid	Sequence 5′-3′
AGR2-D132A-F	pmAGR2D132A	CC AGG ATT ATG TTT GTT *GCC* CCA TCT CTG ACA GTT AG
AGR2-D132A-R		CT AAC TGT CAG AGA TGG *GGC* AAC AAA CAT AAT CCT GG
AGR2-P133A-F	pmAGR2P133A	C AGG ATT ATG TTT GTT GAC *GCA* TCT CTG ACA GTT AGA GC
AGR2-P133A-R		GC TCT AAC TGT CAG AGA *TGC* GTC AAC AAA CAT AAT CCT G
AGR2-S134A-F	pmAGR2S134A	ATT ATG TTT GTT GAC CCA *GCT* CTG ACA GTT AGA GCC G
AGR2-S134A-R		C GGC TCT AAC TGT CAG *AGC* TGG GTC AAC AAA CAT AAT
pEpEX-Y251A-F	pEpEX-CO	T CCG GGT CAG ACC CTG ATC TAT GCT GTT GAT GAA AAA GCAC
pEpEX-Y251A-R		G TGC TTT TTC ATC AAC AGC ATA GAT CAG GGT CTG ACC CGG A
EpCAM-C-EGFP-F	pEGFP-N1	GCT CTC GAG ATG GCG CCC CCG CAG GTC CTC
EpCAM-C-EGFP-R		C GAC CGG TGC ATT GAG TTC CCT ATG CAT CTC
EpCAMY251A-C-EGFP-F	pEpCAM-C-EGFP	G GAT CTG GAT CCT GGT CAA ACT TTA ATT TAT GCT GTT GAT GAA AAA GCA CCT
EpCAMY251A-C-EGFP-R		AGG TGC TTT TTC ATC AAC AGC ATA AAT TAA AGT TTG ACC AGG ATC CAG ATC C

##### Protein Purification

For purification of His-AGR2 WT and its mutant derivatives His-AGR2 D132A, His-AGR2 P133A, His-AGR2 S134A, *E. coli* strains BL21-AI transformed with His-AGR2 WT and mutant plasmids were grown aerobically at 37 °C and 200 rpm in LB broth medium supplemented with ampicillin (100 μg/ml). Cultures were induced at *A_600 nm_* of 0.6–0.8 by the addition of 0.2% arabinose (w/v) for 3 h at 37 °C and 200 rpm. Cell pellets were collected by centrifugation 10,000 rpm for 10 min at 4 °C. Cell pellets were resuspended in buffer A (20 mm Tris-HCL pH8, 150 mm NaCl, 10 mm MgCl_2_, 0.1%Nonidet P-40, 10% glycerol 20 mm Imidazole) supplemented with 0.1 mg/ml lysozyme and incubated on ice for 30 min. The resuspended cells were then sonicated 3 × 15 s on ice with a 30-s interval to prevent overheat using a small probe at 15 amplitude microns. Sonicated cells were then centrifuged at 10,000 rpm for 10 min at 4 °C to remove cell debris. Cell lysates were mixed with 1 ml Ni-NTA agarose (Qiagen) for 1 h before loading into 10 ml column with 35 μm filter pore size (Mobitec) equilibrated with wash buffer A. After several washes with increasing amount of imidazole in wash buffer A and wash buffer B (buffer A with 40 mm Imidazole), the proteins were eluted with the same buffer but with 150 mm Imidazole.

For purification of His-EpEX-CO, *E. coli* strains BL21-DE3 were transformed with the pEpEX-CO construct and were grown aerobically at 37 °C at 200 rpm in LB medium supplemented with ampicillin (100 μg/ml). Cultures were induced at *A*_600 nm_ of 1.0 by the addition of 1 mm isopropyl β-d-1-thiogalactopyranoside for 4 h at room temperature and 200 rpm. Cell pellets were collected by centrifugation at 10,000 rpm for 10 min at 4 °C. The pellets were resuspended in resuspension buffer (10% Sucrose (w/v), 50 mm HEPES pH8.0). The pellets were supplemented with final concentrations of 0.5 m NaCl, 1 mm Benzamidine, 1 mm DTT, 0.1% Triton X-100, 1x Protease Inhibitor Cocktails Tablet (Roche) and 0.1 mg/ml lysozyme followed by incubation on ice for 30 min. Cells were then sonicated 3 × 15 s on ice with a 30-s interval to prevent overheat using a small probe at 10 amplitudes. Solid urea powder (7 m) was added directly to the sonicated cells and incubated in rotating wheel at 4 °C until the urea dissolves. Cell pellets containing cellular debris were collected by centrifugation at 10,000 rpm and the lysate was added to 0.3 ml Ni-NTA agarose (Qiagen). The Ni-NTA agarose beads with bound His-EpEX-CO were transferred to a microfuge tube and were incubated overnight in a rotating wheel at 4 °C. The beads were washed several times with wash buffer (50 mm HEPES pH8.0, 0.5 m NaCl, 1 mm Benzamidine, 1 mm DTT, 2 m Urea, 10 mm Imidazole). His-pEpEX-CO was eluted from the beads by addition of elution buffer (50 mm HEPES pH8.0, 0.5 m NaCl, 1 mm Benzamidine, 100 mm DTT, 0.3 m Imidazole).

##### Solid-phase Binding Assay

Purified protein His-EpEX-CO as stated amounts was immobilized on polystyrene microtitre plates in 0.1 m NaHCO_3_ (pH 8.6) overnight at 4 °C. Alternatively, biotin-labeled AGR2 binding peptide at saturating amounts (5 μg/well in dH_2_0) was captured onto a microtitre plate coated with streptavidin (1 μg/well in dH_2_0) incubated at 37 °C overnight. Following washing in 4x PBS supplemented with 0.1% (v/v) Tween-20 (PBS-T), nonreactive sites were blocked using 3% (w/v) BSA in PBS-T. A titration of the protein and/or peptide of interest was added in 3% BSA in PBS-T for 1 h at room temperature. Remaining free protein in solution was removed and washed with 6 × 0.1% PBS-T, and the portion of the partner protein bound to the immobilized protein was quantified through detection via AGR2 polyclonal primary antibody K47 followed by anti-rabbit/HRP secondary antibody and quantified using Fluoroskan Ascent FL (Thermo Scientific) as relative light unit (RLU).

##### SDS-PAGE and Immunoblot Analyses

Cells were lysed in urea buffer (7 m urea, 0.1 m DTT, 0.1% Triton X-100, 25 mm NaCl, 20 mm HEPES–KOH pH 7.6, 5 mm NaF, 2 mm Na_3_VO_4_, 2.5 mm Na_4_P_2_O_7_). Proteins were quantified using Protein Assay Dye Reagent (Bio-Rad) according to Bradford assay (29). Proteins were resolved by SDS-PAGE using 12–15% gels according to (30) and transferred onto nitrocellulose membranes (Amersham Biosciences Protran, GE Healthcare). Membranes were probed with primary antibodies, followed by secondary antibodies conjugated to HRP. Bound antibody was detected by enhanced chemiluminescence (ECL) as RLU.

##### Proteins Used for Hydrogen-Deuterium Exchange Mass Spectrometry

The amino acid sequences of the AGR2 recombinant proteins with N-terminal His-tag EpCAM recombinant proteins are:

1. wild-type AGR2: MSYYHHHHHHLESTSLYKKAGFEGDRTMRDTTVKPGAKKDTKDSRPKLPQTLSRGWGDQLIWTQTYEEALYKSKTSSKPLMIIHHLDECPHSQALKKVFAENKEIQKLAEQFVLLNLVYETTDKHLSPDGQYVPRIMFVDPSLTVRADITGRYSNRLYAYEPADTALLLDNMKKALKLLKTEL; and
2. mutant AGR2^S134A^: MSYYHHHHHHLESTSLYKKAGFEGDRTMRDTTVKPGAKKDTKDSRPKLPQTLSRGWGDQLIWTQTYEEALYKSKTSSKPLMIIHHLDECPHSQALKKVFAENKEIQKLAEQFVLLNLVYETTDKHLSPDGQYVPRIMFVDP*A*LTVRADITGRYSNRLYAYEPADTALLLDNMKKALKLLKTEL.
3. wild-type EpCAM: QEECVCENYKLAVNCFVNNNRQCQCTSVGAQNTVICSKLAAKCLVMKAEMNGSKLGRRAKPEGALQNNDGLYDPDCDESGLFKAKQCNGTSMCWCVNTAGVRRTDKDTEITCSERVRTYWIIIELKHKAREKPYDSKSLRTALQKEITTRYQLDPKFITSILYENNVITIDLVQNSSQKTQNDVDIADVAYYFEKDVKGESLFHSKKMDLTVNGEQLDLDPGQTLIYYVDEKAPEFSMQGLK.
4. mutant EpCAM^Y251A^: QEECVCENYKLAVNCFVNNNRQCQCTSVGAQNTVICSKLAAKCLVMKAEMNGSKLGRRAKPEGALQNNDGLYDPDCDESGLFKAKQCNGTSMCWCVNTAGVRRTDKDTEITCSERVRTYWIIIELKHKAREKPYDSKSLRTALQKEITTRYQLDPKFITSILYENNVITIDLVQNSSQKTQNDVDIADVAYYFEKDVKGESLFHSKKMDLTVNGEQLDLDPGQTLIYAVDEKAPEFSMQGLK.

The proteins were purified as described above and exchanged using gel filtration into a buffer with the composition of 20 mm Tris pH8.0, 150 mm NaCl and 10 mm MgCl_2_. The 16mer AGR2-interacting peptide has the amino acid sequence SGSG-HLP*TTIYY*GPPG and the stock solution is at 5 mg/ml in DMSO. The peptide: AGR2 protein ratio was at 10:1 to give a final concentration of the AGR2-binding peptide at 10 μm and a final concentration of DMSO was 1%.

##### Experimental Design and Statistical Rationale for Hydrogen-deuterium Exchange Mass Spectrometry

##### a. Sample Preparation of AGR2 Proteins

Deuteration of the His-AGR2 WT and His-AGR2^S134A^ proteins was initiated by a sequential 10-fold dilution into a deuterated buffer (20 mm Tris-HCl, pD 7.6, 150 mm NaCl, and 10 mm MgCl*_2_). In the peptide-binding mapping experiment, His-AGR2 WT at 1 μ*m final concentration was incubated with a final concentration of peptide at 10 μm (31). This higher concentration of peptide was used as we had previously defined the *K_d_* to be 15–45 μm using fluorescence polarization (31). Samples were processed in biological replicates (*e.g.* the deuteration reaction and sample processing were performed on four different days) with representative data from a time course of deuteration (AGR2 without and with peptides or wt-AGR2 compares to the AGR2^S134A^ mutant) highlighted in each experiment. Multiple peptide replicates were used to select data and the HDExaminer software selects peptides with high confidence meaning the selected peptides are identified in all submitted MS data of all samples (nondeuterated and deuterated time intervals). When peptic peptides are not identified in all time course samples, then the peptide is excluded from the analysis. Shorter peptides were used for HDX data interpretation (such as Figs. 4 and 5) and all set of the peptides identified by LC-MS/MS and submitted for HDExaminer evaluation are included in the Supplementary Figures. Protein was incubated with peptide for 30 min prior to the exchange. The deuterium exchange was carried out at room temperature and was quenched by the addition of 10 μl of 1 m HCl in 1 m glycine at 30 s, 1 min, 3 min, 10 min, 30 min, 1 h, and 3 h followed by rapid freezing in liquid nitrogen.

##### b. Sample Preparation of MDM2 Protein

Deuteration of full-length MDM2 without or with Nutlin-3 was performed as described previously (32). Full-length MDM2 was purified was expressed with a glutathione-S-transferase-tag from pGEX-6P1 and purified from *Escherichia coli* lysates using glutathione beads (GE Healthcare). Cells were lysed with 10% sucrose, 50 mm Tris-HCl (pH 8.0), 150 mm NaCl, 0.5% Nonidet P-40, 1 mm DTT, 1 mm benzamidine, 150 μg/ml lysozyme, and protease inhibitors for 30 min on ice prior to sonication. The sonicated supernatant was incubated with glutathione beads for 2 h at 4 °C with rotation, followed by three washes with 20 mm Hepes (pH7.5), 150 mm NaCl, and 1 mm DTT. Protein was cleaved off the beads using Precision protease (GE Healthcare) into a buffer with 20 mm Hepes (pH 7.5), 150 mm NaCl, 1 mm DTT, and 10% glycerol. The Full-length MDM2 (2 μm) was incubated for 30 min at room temperature in a final 5 μl volume of buffer (20 mm Tris-HCl, pH 7.6, 150 mm NaCl, and 10 mm MgCl_2_) with DMSO at 1% or with Nutlin-3 (8 μm final concentration). The deuteration was initiated by a 10-fold dilution into a deuterated buffer (25 mm Hepes, pD 7.5, and 150 mm NaCl) and was incubated with Nutlin-3 for 60 min before the exchange. Aliquots were taken various times (as in Fig. 2) and reactions were quenched by the addition of 10 μl of 1 m HCl in 1 m glycine followed by rapid freezing in liquid nitrogen.

##### c. Sample Preparation of EpCAM Proteins

EpCAM WT or EpCAM^Y251A^ proteins purified with 1 mm DTT in the final elution buffer (1 μg; as tested in Fig. 8) was incubated for 60 min at room temperature in a final 5 μl volume of buffer (20 mm Tris-HCl, pH 8, 150 mm NaCl, and 10 mm MgCl_2_) or with the AGR2 WT protein (2.2 μg in the matched buffer) prior to initiating the deuteration exchange. Under these conditions, the molar ratio of AGR2: EpCAM protein was 4:1 and incubations were carried out for 60 min at room temperature. The deuteration was then initiated by a sequential 10-fold dilution into a deuterated buffer (20 mm Tris-HCl, pD 7.6, 150 mm NaCl, and 10 mm MgCl_2_) carried out at room temperature. The EpCAM internal disulfide bonds were subsequently reduced with dithiothreitol (14 mm final concentration, 2 min) followed quenching by the addition 0.875 m HCl in 1 m glycine with pepsin (0.042 mg/ml final concentration).

##### d. LC-MS/MS Analysis and Data Evaluation

EpCAM samples were immediately submitted to analysis (because of aggregation following freeze-thaw cycles (data not shown) but the remaining samples (AGR2 or MDM2) were snap-frozen, thawed, and injected onto an immobilized pepsin column (15 μl bed volume, flow rate 100 μl/minutes in 2% acetonitrile/0.05% trifluoroacetic acid). Peptides were trapped and desalted on-line on a peptide microtrap (Michrom Bioresources, Auburn, CA) for 3 min at flow rate 50 μl/minutes. The peptides were eluted onto an analytical column (Jupiter C18, 1.0 × 50 mm, 5 μm, 300Å, Phenomenex, CA) and separated using a linear gradient elution of 10% B for 2 min, followed by 17 min isocratic elution at 40% B. Solvents were: A - 0.1% formic acid in water, B - 80% acetonitrile/0.08% formic acid. The immobilized pepsin column, trap cartridge, and the analytical column were kept at 1 °C. Mass spectrometric analysis was carried out using an Orbitrap Elite mass spectrometer (Thermo Fisher Scientific) with ESI ionization on-line connected with a robotic system based on the HTS-XT platform (CTC Analytics). The instrument was operated in a data-dependent mode for peptide mapping (HPLC-MS/MS). Each MS scan was followed by MS/MS scans of the top three most intensive ions from both CID and HCD fragmentation spectra. Tandem mass spectra were searched using SequestHT against the cRap protein database (ftp://ftp.thegpm.org/fasta/cRAP) containing sequences of the AGR2 proteins with the following search settings: mass tolerance for precursor ions of 10 ppm, mass tolerance for fragment ions of 0.6 Da, no enzyme specificity, two maximum missed cleavage sites, and no fixed or variable modifications were applied. The false discovery rate at peptide identification level was set to 1%. Sequence coverage was analyzed with Proteome Discoverer software version 1.4 (Thermo Fisher Scientific). Analysis of deuterated samples was performed in HPLC-MS mode with ion detection in the orbital ion trap and the data were processed using HDExaminer (Sierra Analytics). Graphs summarizing deuteration kinetics were plotted using the Draw H/D Protection Plot (34). All peptide plots (graphs showing % deuteration over a time course) are summarized in supplemental Figs. S1–S8. The mass spectrometry proteomics data have been deposited to the ProteomeXchange Consortium via the PRIDE repository with the data set identifier PXD005782.

##### Fluorescent Microscopy of AGR2 and EpCAM Proteins

MCF7 breast cancer cells were grown in DMEM (10% FCS). MCF-7 cells were seeded onto glass coverslips and incubated at 37 °C overnight. Cells were transiently transfected with DNA constructs as described using Lipofectamine 2000 (Thermo Fischer Scientific) according to manufacturer's protocol and incubated 37 °C for 24 h. Cells were then washed three times with sterile PBS before incubating with 4% paraformaldehyde for 15 min to fix the cells. Coverslips were mounted with DAPI nuclear stain (Invitrogen) diluted in Fluorescent Mounting Medium (Dako) and viewed using Zeiss Axioplan 2 fluorescent microscope.

##### Proximity Ligation Assays

The method was performed using the *Duolink* kit (Sigma Aldrich; 92014) according to manufacturers recommendations. Cells were grown in 24 well plates over glass coverslips (diameter: 16 mm). Cells were transfected as indicated in the legends of Figs. 14 and 15. Twenty-four hours later, cells were fixed with 4% paraformaldehyde in PBS for 15 min at RT, permeabilized using 0.25% TritonX-100 in PBS for 10 min, and blocked with 3% BSA in PBS for 30 min. Antibodies from different species (as described in the legends of Figs. 13–15) were incubated on the slides, with combinations of AGR2 mouse (4.1 (34)) or rabbit antibodies (K47) and EpCAM antibodies (rabbit antibody, HPA026761, from Sigma, or mouse antibody, VU-1D9, from Calbiochem) at dilutions of 1:250 overnight at 4 °C. Proximity ligation was measured using the OLIGO Duolink designated protocol (35) using anti-mouse and anti-rabbit probes (Sigma, probe product number are 92004 (mouse minus) and 92002 (rabbit plus)). Then coverslips were stained with DAPI and mounted. Images were captured at 40X by an Olympus BX51 epifluorescence microscope.

## RESULTS

### 

#### 

##### Defining the Specificity of TTIYY Peptide Binding to AGR2 in Crude Cancer Cell Lysates

**Fig. 1.**
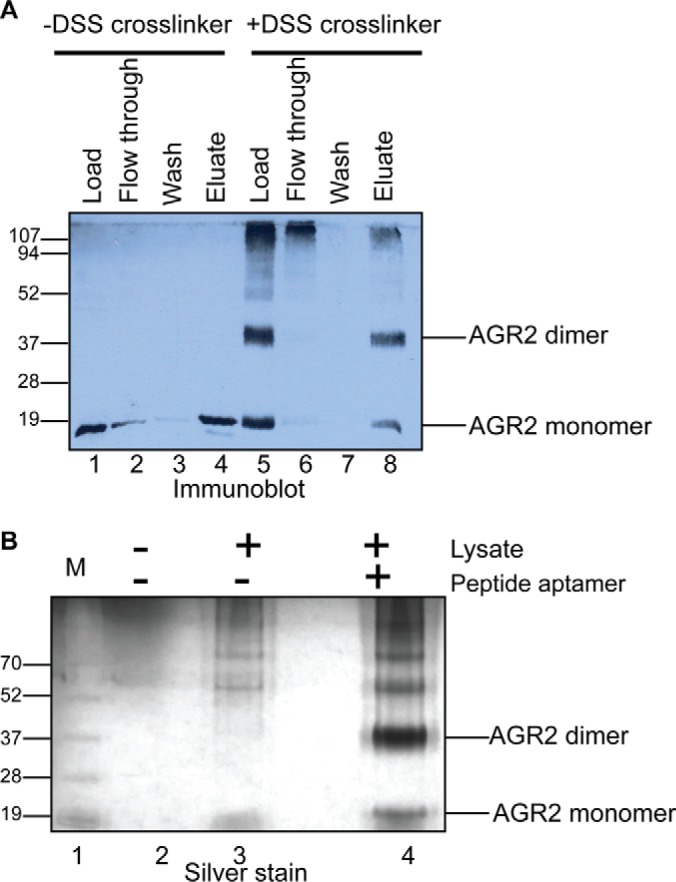
**The specificity of an AGR2-binding peptide aptamer for use in the affinity purification of *in vivo* cross-linked AGR2 protein from crude lysates**. *Affinity purification of AGR2 from crude cell lysates*. MCF7 cells grown in media with 10% FCS were incubated with DMSO (lanes 1–4) or with a fixed concentration of the cell-membrane permeable cross-linker DSS (lanes 5–8) for 1 h at 37 °C. Cells were harvested, lysed using a Tris-HCl (pH 8.0) buffer containing 1% Nonidet P-40, lysates were incubated with an optimized biotinylated peptide aptamer (named A4) linked to streptavidin beads, which can be used to affinity purify the AGR2 protein in crude cell lysates (24). The input lysates (Load, lanes 1 and 5), flow-through fractions (lanes 2 and 6), washes (lanes 3 and 7), and eluates (lanes 4 and 8) were separated by electrophoresis. The protein in the polyacrylamide gel was (A) immunoblotted to determine whether intermediary species of cross-linked AGR2 could be affinity purified and (B) stained with silver to measure total protein captured. In part B, streptavidin beads were incubated with buffer only (lane 1), lysates (lane 2), and lysates with preconjugated peptide-A4-beads (lane 3). The 18 kDa and 36 kDa cross-linked and affinity purified silver-stained proteins (arrows, lane 3 *versus* lane 2) were excised using trypsin and AGR2 protein was confirmed present by MALDI-TOF mass spectrometry (data not shown; dimeric excised band; only human AGR2 peptides were detected, Score = 161; queries matched = 4; emPAI = 0.86).

Peptide A4 with the sequence HLP*TTIYY*GPPG (containing the key functional pentapeptide sequence underlined) was previously shown to bind AGR2 (24). We evaluated the specificity of the peptide in binding to and affinity purifying monomeric or dimerized AGR2 protein from human cell lysates. MCF7 cells were left untreated or treated with a cell-membrane permeable cross-linker that stabilizes the dimer via a K95-K95 homodimeric cross-link (23). When lysates from untreated cells are incubated with streptavidin beads coated with Peptide A4, monomeric AGR2 protein can be affinity purified from the crude lysate (Fig. 1*A*, lane 4 *versus* Load, Flow-though, and wash, lanes 1–3). In addition, the chemically stabilized dimer (Fig. 1*A*, lane 5) can also be affinity purified using the Peptide A4 coupled beads (Fig. 1*A*, lane 8 *versus* 5–7). We evaluated the specificity of the peptide in affinity capture of AGR2 protein from beads without peptide (Fig. 1*B*, lane 2) and beads with peptide (Fig. 1*B*, lane 3). These data indicate that Peptide A4 can be used to affinity purify dimeric or monomeric protein and suggest the bioactivity of this peptide motif in crude lysates to AGR2 is relatively specific. However, the silver stain gel does reveal high molecular mass proteins that might either be binding to the peptide direct or through association with AGR2. These potential AGR2 binding proteins that were in these fractions were not evaluated. Nevertheless, the relative specificity of this peptide for AGR2, as defined by its ability to affinity purify the protein from crude lysates, suggests that this feature of AGR2 might selectively drive (some of) its protein-protein interaction functions. Thus, we continued to characterize this specific peptide binding activity to discover bonafide client proteins that harbor this consensus peptide motif.

##### Mapping AGR2 Peptide Docking Sites Using Hydrogen-Deuterium Exchange Mass Spectrometry

We next aimed to determine whether we could detect the peptide A4 binding to a specific region of AGR2 or whether its binding might “denature” or destabilize AGR2 protein. Such data might indicate whether specific peptide binding is a function of AGR2 or whether proteins with this motif that bind AGR2 impact on its' structure. When a protein binds to a ligand the kinetics of hydrogen-deuterium exchange can be altered at amide bonds that can reflect conformational changes or binding events (36) and this method was used to determine whether the peptide-A4 interacts with any specificity on AGR2 and/or whether it alters AGR2 protein conformation. We had previously set up hydrogen-deuterium exchange using the N-terminal domain of MDM2 protein (amino acids 1–126) as a model system to define effects of ligand binding on the exchange reaction (32) and the methodology in this study used *ExPro Script* to measure deuteration rates. Because this data was published, a new software package became available for use by life scientists (*HDExaminer*). This provides an additional robust tool to study protein dynamics in solution using deuterium exchange methodologies. We first evaluated, therefore, MDM2-ligand binding using HDExaminer as a positive control to measure the integrity of the data acquired using our methodology including; sample acidification after deuteration, freezing, thawing, and injection onto an immobilized pepsin column (flow rate 100 μl/minute), peptide trapping and desalting using a peptide microtrap for 3 min at flow rate 50 μl/minute and peptide separation using an analytical column with a linear gradient.

**Fig. 2.**
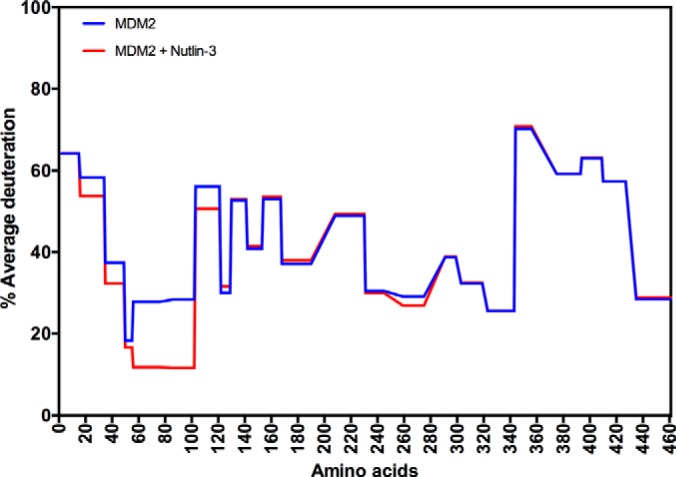
**Establishing methodology for measuring the effects of a ligand on the target protein MDM2 using hydrogen-deuterium exchange mass spectrometry**. MDM2 protein (2 μm final concentration) was assembled with DMSO or with Nutlin-3 (8 μm) at room temperature for 30 min, as described previously for the N-terminal domain of MDM2 (amino acids 1–126) (32). The reactions were then diluted with D_2_O by sequentially adding, slowly with mixing, 5.14 μl, 10 μl, 20 μl, and 27 μl of D_2_O. The reactions were incubated from 40 to 18,000 s and quenched with 3 μl of 0.87 m HCl with 1 m Glycine, frozen, and processed for pepsinization as in the materials and methods. The deuterium exchange rates of individual peptides (supplemental Fig. S1) is summarized using the HDX exchange plots for the 300-s deuteration time course.

MDM2 is an oncoprotein with a druggable peptide-binding pocket that can be studied using the ligand Nutlin-3 (37). This ligand mimics the p53 peptide and binds stably within its N-terminal hydrophobic pocket. Full-length MDM2 was deuterated without or with Nutlin-3 using the hydrogen-deuterium exchange methodology (Fig. 2 and supplemental Fig. S1). Purified full-length MDM2 (2 μm) was incubated with DMSO or the ligand Nutlin-3 (1:4 ratio) for one hour, diluted in a 10-fold excess of D_2_O buffer, and processed with immobilized pepsin (as described above) to define changes in deuteration of peptic products. The data show, as expected, suppression of deuteration at the N-terminal drug-binding pocket of MDM2 from amino acids 56–103 (Fig. 2). Suppression of deuteration from amino acids 245–265 might reflect allosteric effects of the drug on MDM2 protein conformation or it might reflect an interaction with Nutlin-3 at this site (Fig. 2) (38). Amino acid residues ranging from 20–50 also show suppressed deuteration (Fig. 2) suggesting conformational changes occur outside and adjacent to the Nutlin-3 binding pocket. Visualization using a butterfly plot that incorporates several parameters including time and % deuteration further provides perspective on the impact of ligand on the overall conformational dynamics of full-length MDM2 (supplemental Fig. S2). These results establish HDExaminer in combination with the experimental approach as a reliable methodology for mapping the effects of a ligand on a protein.

**Fig. 3.**
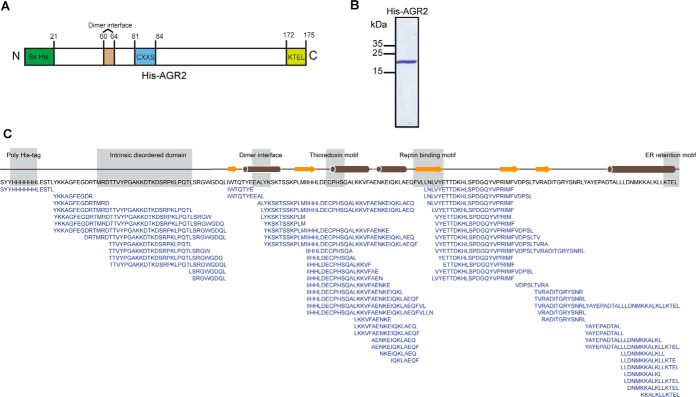
**Peptic coverage of AGR2 protein in the seven-point deuteration reaction time course.** *A*, Gene structure of the recombinant AGR2 protein. The panel highlights the functional motifs within recombinant human AGR2; the poly-His-tag used for nickel affinity purification; an N-terminal intrinsically disordered region; a dimerization motif; a thioredoxin fold, the Reptin docking site, and the ER retention site. *B*, Coomassie Blue staining of purified His-AGR2 showing the major band at 20 kDa under reducing SDS-PAGE condition. *C*, Peptic peptides recovered from ligand-free mature AGR2 protein (with the N-terminal 20 amino acids containing the hydrophobic ER leader sequence removed). The proteolytic peptide fragments can be grouped into four regions based on extents of recovery after mass spectrometry; (i) The N-terminal region containing the poly his-tag and amino acids Arg21-Leu52 containing the intrinsically disordered region that plays a negative regulatory role in dimer stability (23) (7); (ii) a central grouping from Ile53 to Asn108, containing the beginning of the dimerization motif (60-EALYK-64), the CxxS thioredoxin fold, and the Reptin binding motif (104-FVLLNLVY-111); (iii) a third pepsin resistant region from amino acids 109–130; and (iv) a region of a cluster of overlapping peptic fragments including the specific peptide binding domain (this study) and the degenerate KTEL endoplasmic reticulum retention site.

Using this optimized protocol, AGR2 protein (1 μm; Fig. 3*A* and 3*B*) was incubated without ligand or with Peptide SGSGHLPTTIYYGPPG (10 μm) to allow complex formation. The samples were then diluted 10-fold in D_2_O buffer over a time course from 30 to 10800 s, reactions were quenched by acidification and freezing, then the thawed samples were subjected to pepsin digestion (supplemental Fig. S3 contains all of the primary raw graphical data and supplemental Fig. S4 visualizes overall datasets using a butterfly plot). The peptic coverage of a representative experiment using ligand-free AGR2 is shown in Fig. 3*C*. Peptic cleavage is not completely random throughout the sequence and can generally be grouped into four distinct domains (Fig. 3*C*). These domains contain (1) the N-terminal domain from amino acids 21–53 that harbor intrinsically disordered sequences that negatively regulate dimer formation (7) (23); (2) the central domain containing amino acids 54–108 that harbor the dimer interface (7), the CXXS thioredoxin fold, and the Reptin docking site (20); (3) a region N-terminal to the specific peptide-binding domain (amino acids 109–130; this study); and (4) the specific peptide binding domain adjacent to the ER retention site (Fig. 3*C*). Preferred cleavage sites or hotspots of pepsin cleavage can be visualized such as Thr23 (in the N-terminal disordered region; Fig. 3), Tyr63 (within the dimer interface of 60-EALYK-64), Ile74 (N-terminal to the CxxS motif), and Val110 (within the Reptin binding motif (20); Fig. 3*C*). The existence of four regions with a degree of pepsin preference, suggests some secondary/tertiary conformation is maintained on acidification. It is interesting to note that very few peptides were obtained covering the complete dimerization motif from amino acids 60–64 (Fig. 3C; highlighted by bracketed amino acids within sequencing EALYK). In fact, a hotspot of cleavage is at Tyr63 (within the dimer interface of 60-EALYK-64) suggesting this region is relatively more susceptible to proteolysis when in the acidified and denatured state.

**Fig. 4.**
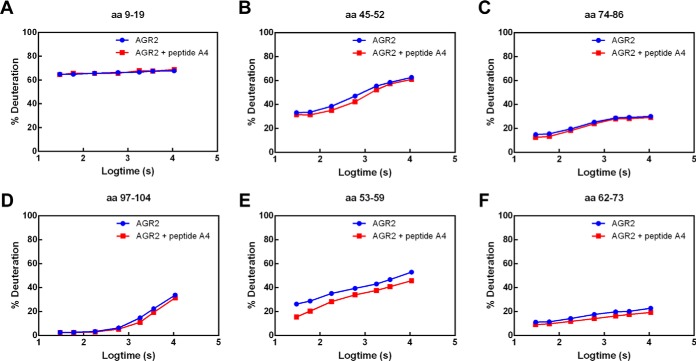
**Representative deuteration rates of peptides derived from the N-terminal domain of AGR2 after binding of peptide A4.** Ligand-free AGR2 or AGR2-peptide A4 complexes were deuterated over a 7-point time course from 30 s to 10,800 s followed by acidification, pepsinization, and separation of fragments using mass spectrometry. *A–C*, Representative segments of AGR2 protein that do not exhibit significant changes in deuteration after peptide A4 binding over the time course are highlighted including; *A*, YKKAGFEGDRT from the N-terminal HIS tag; *B*, a peptic fragment N-terminal to the dimerization interface containing amino acids 45-SRGWGDQL-52; *C*, a peptide containing the thioredoxin fold, from amino acids 74-IIHHLDECPHSA-86; and (*D*) a peptic fragment adjacent to the Reptin docking site, containing amino acids 97-IQKLAEQF-104. Segments of AGR2 protein that do show a degree of deuterium suppression over the entire time course included; E, a peptide containing amino acids 53-IWTQTYE-59 just adjacent to and N-terminal to the dimerization motif; and (*F*) peptide containing half of the dimerization motif from amino acids 62-LYKSKTSSKPLM-73. The data are plotted as % of deuteron exchange as a function of time (log10 in seconds; from 30, 60, 180, 600, 1800, 3600, and 10800).

On peptide binding by AGR2, peptic fragments within the intrinsically disordered N-terminal domain exhibited marginal changes in deuteration (Fig. 4*A*–4*D*), suggesting that this region is not significantly impacted on peptide binding. Such small mass peptic fragments included the N-terminal tag (YKKAGFEGDRT; Fig. 4*A*) and a peptic fragment N-terminal to the dimerization interface (45-SRGWGDQL-52; Fig. 4*B*). Interestingly, over the time course of deuteration, the peptide 53-IWTQTYE-59 just adjacent to the dimerization motif was attenuated in its deuteration (Fig. 4*E*) and marginal deuterium suppression over the time course from amino acids 62-LYKSKTSSKPLM-73 was observed. These latter data suggest that peptide A4 binding can impact on this dimeric interface. There is a degree of specificity in this deuterium suppression around the dimeric interface because the peptide 74-IIHHLDECPHSA-86 containing the thioredoxin fold does not exhibit significant deuteration changes in the presence of peptide A4 (Fig. 4*C*). Nor does a small mass peptic fragment adjacent to the Reptin docking site, 97-IQKLAEQF-104, exhibit significant changes in deuteration (Fig. 4*D*).

**Fig. 5.**
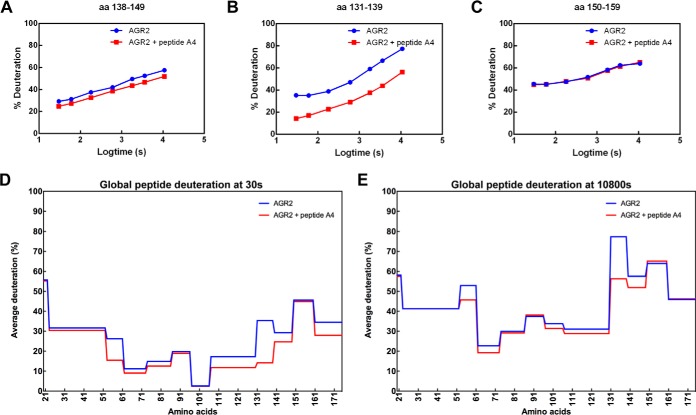
**Representative deuteration rates of peptides derived from the C-terminal domain of AGR2 after binding of peptide A4.** Ligand-free AGR2 or AGR2-peptide A4 complexes were deuterated over a 7-point time course from 30 s to 10,800 s followed by acidification, pepsinization, and separation of fragments using mass spectrometry. The C-terminal domain showed the most significant global changes (suppression) in deuteration of individual peptic fragments after peptide A4 binding across the time course are highlighted, including: *A*, Peptide containing amino acids 138-RADITGRYSNRL-149, and *B*, the peptide contiguous and N-terminal to (*A*) containing amino acids 131-VDPSLTVRA-139. *C*, The peptide contiguous and C-terminal to (*A*) containing amino acids 150-YAYEPADTAL-159 exhibited minimal deuteration changes on peptide A4 binding. *D* and *E*, A visualization of the global changes in peptide deuteration without (blue) and with peptide A4 ligand (red) after (*D*) 30 s of incubation in D_2_O or (*E*) after 10,800 s of incubation in D_2_O. The data are plotted as % of deuteron exchange as a function of time (log10 in seconds; from 30, 60, 180, 600, 1800, 3600, and 10800).

The C-terminal domain showed the most significant changes in deuteration after peptide binding across the time course (Fig. 5). For example, peptide 138-RADITGRYSNRL-149 exhibited consistent suppression of deuteration throughout the entire time course (Fig. 5*A*) like the peptides surrounding the dimer interface. However, the most significantly suppressed minimal peptic fragment over the entire time course of deuteration was 131-VDPSLTVRA-139 (Fig. 5*B*). By comparison, the minimal peptide fragment 150-YAYEPADTAL-159 exhibited minimal deuteration changes on peptide binding (Fig. 5*C*). A visualization of the global changes in peptide deuteration (from Fig. 5) deuteration after early, after 30 s (Fig. 5*D*) or later, after 10,800 s (Fig. 5*E*), further highlights the most dominant impact of peptide A4 on the structural loop from amino acids 131–135 (Fig. 5*E*) but also extending C-terminal into the contiguous extended polypeptide chain from amino acids 138–149 (RADITGRYSNR). Presumably, once AGR2-peptide complexes reach some equilibrium after diluting 10-fold with D_2_O and incubations for 10,800 s, there is enhanced deuteration within the dimerization motif (compare Fig. 5*E* to 5*D*). This suggests that specific peptide binding might destabilize the dimer into the monomeric state.

**Fig. 6.**
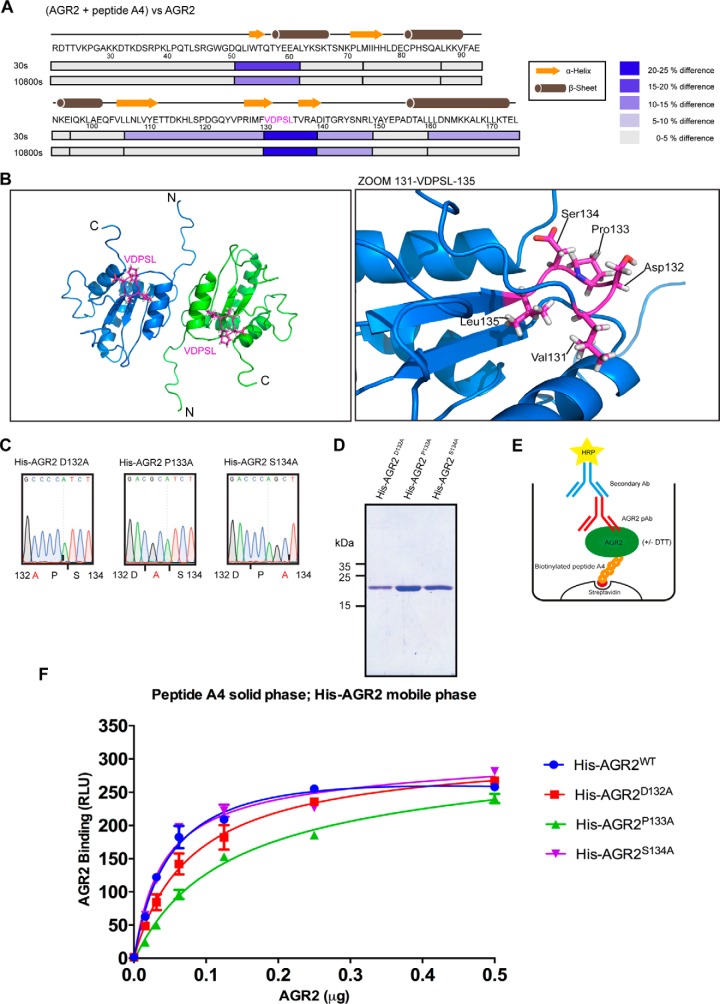
**Mutations in the dominant deuteration responsive motif impact on AGR2 peptide binding activity *in vitro*.** *A*, A secondary structure summary of changes in deuteration in the presence of the consensus peptide. The diagram shows the full amino acid sequence of the mature AGR2 protein (amino acids 21–175) with the alpha helices and β-sheets highlighted. The block colors highlight the changes in fold deuteration after 30 s or 10800 s, with dominant changes at the region 131–139 (in between two β-sheets) and the dimer interface (a 50–60). *B*, A representation of the minimal deuteration responsive motif from amino acids 131–135 motif (highlighted in pink) in AGR2 (PDB code: 2LNS) that is most significantly suppressed by peptide A4 binding. The main amino acids of focus were D132, P133, and S134 flanked by the hydrophobic amino acids V131 and L135. *C*, Data showing alanine substitution mutations generated at codons D132, P133, and S134 in bacterial expression plasmids with the corresponding DNA sequencing chromatogram traces of three of AGR2 peptide docking site mutations at position Asp132 to Ala, Pro133 to Ala, and Ser134 to Ala. *D*, An SDS-Coomassie blue gel showing the relative purity of the indicated mutant proteins expressed in *E. coli* after nickel affinity purification. *E–F*, An ELISA assay was developed to measure the binding of AGR2 to synthetic biotinylated peptide A4 captured on the streptavidin coated solid phase. Reactions were added to the solid phase to measure binding to biotinylated peptide A4 on the solid phase. The data plot the binding of AGR2 in relative light units (RLU) as a function of increasing wt or mutant AGR2 protein isoforms, as indicate (in μg) that was quantified using AGR2 specific antibody.

The region containing amino acids 131–135 (the VDPSL-containing loop or turn) exhibited consistent suppression at early and late time points and it might project a “flexible docking site”. This core VDPSL motif (Fig. 6*A* and 6*B*) was mutated in full-length AGR2 at the positions of D132, P133, and S134 to determine if alterations in PTTIYY peptide binding occur. The proteins were purified (Fig. 6*C* and 6*D*) and tested for changes in consensus site peptide binding. The mutant AGR2^D132A^ exhibited a high degree of proteolysis and instability in bacteria and its yield was lower than wild-type AGR2 (data not shown) suggesting it might have a different conformation. Nevertheless, the AGR2^D132A^ mutant was evaluated in consensus site peptide binding assays. Biotinylated peptide A4 was captured on the streptavidin solid phase (Fig. 6*E*). The wild-type and docking-site mutated AGR2 proteins were titrated to determine whether these mutations increase, decrease, or have no effect on biotinylated peptide A4 binding. AGR2^S134A^ exhibited wild-type levels of peptide binding, AGR2^D132A^ exhibited attenuated levels of peptide-binding, and the AGR2^P133A^ exhibited the lowest amounts of peptide binding (Fig. 6*F*). The marginally attenuated activities of the AGR2 mutants encoded by the P133A and D132A alleles suggest that these two amino acids play more dominant roles than the S134 side-chain in biotinylated peptide A4 peptide docking.

##### Developing a Consensus Peptide-binding Motif for AGR2

AGR2 is an ER-resident molecular chaperone that plays a role in the maturation of cysteine-rich receptors such as MUC5 (10) and EGFR (39). To our knowledge, AGR2 protein is relatively unique as a “chaperone” in possessing a sequence-specific peptide binding activity (24). It was the main aim of this study to determine whether such a protein-protein interaction plays any role in binding to potential client proteins. If so, this would place a potential filtering step for client proteins with such a motif on AGR2-dependent processing.

**Fig. 7.**
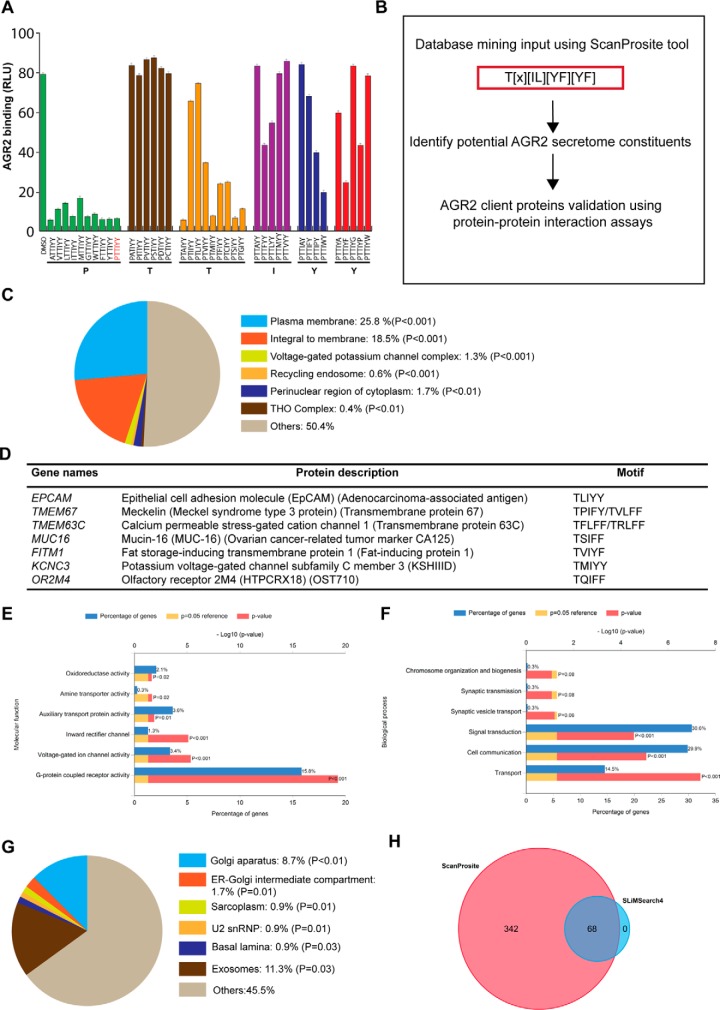
**Mining of the human proteome for proteins containing the AGR2 linear peptide consensus motif.** *A*, The PTTIYY hexapeptide was previously defined as a minimal peptide sequence that binds to AGR2 (24). A mutational scan library was synthesized containing a subset of amino acid substitutions at positions 1–6, from left PTTIYY. The substitutions included small hydrophobic (L, V, I, M, A, G), bulky hydrophobic (W, F, P), charged (D, C) and hydrophilic (S, T) of the linear peptide motif. The peptides sequences created are shown on the X-axis. The biotinylated peptide A4 was bound to the streptavidin-coated solid phase and fixed amounts (1 μg) of AGR2 protein with 100 ng of the indicated synthetic peptide was added. AGR2 binding was detected using a secondary antibody and binding is measured in as RLU. The data revealed that amino acids at positions 2, 4, 5, and 6 are relatively fixed whereas changes at positions 1 and 3 can be relatively well tolerated. *B*, Schematic illustrating strategy to find novel AGR2 client proteins using linear peptide motif database mining. The AGR2 linear peptide consensus motif was used as input using a *ScanProsite* tool (http://prosite.expasy.org/scanprosite/) and the human proteome database was screened to identify proteins containing the motif. *C*, The scan resulted in 409 protein hits when splice variants were excluded (supplemental Tables S1 and S2). The hits were scored as subcellular localization using *FunRich* (v2.1.2) (62) where the majority of the proteins found were membrane proteins. A large proportion of the hits were membrane-related proteins, which foreshadows AGR2 function in receptor maturation. Enriched terms were ranked by *p* value (Hypergeometric test). *D*, Representative of possible AGR2 binding proteins is shown containing the consensus peptide-binding motif. *E*, Bar graph of molecular function overrepresented in AGR2 linear peptide motif hits. *F*, Bar graph of biological processes overrepresented in AGR2 linear peptide motif hits (Supplemental Tables 1 and 2). The percentage of genes linking to the individual enriched terms were ranked by *p* value and are shown together with the *p* value from the Hypergeometric test (depicted in red) and the reference *p* = 0.05 value (depicted in yellow). *G*, The AGR2 linear peptide consensus motif was used as input using the SLiMSEARCH4 linear motif discovery tool (http://slim.ucd.ie/slimsearch/index.php) and the human proteome database was screened to identify proteins containing the motif. Enriched terms were ranked by *p* value (Hypergeometric test). *H*, Venn diagram highlighting the overlap and number of linear motifs identified between *ScanProsite* and *SLiMSEARCH4* tools.

The originally optimized AGR2-binding peptide, PTTIYY, was subjected to mutagenesis to produce a synthetic peptide library in which each of the six positions was mutated to a range of amino acids that are either charged, hydrophobic (bulky), and hydrophobic (small) (Fig. 7*A*). The extent to which AGR2 binding to biotinylated Peptide A4 was changed in the presence of an excess of non-biotinylated 'competitor' peptide was used to define the consensus site. Relative to the positive control of AGR2 binding being inhibited by wt-peptide PTTIYY (Fig. 7*A*, far left bar), mutating each position resulted in the production of the consensus Tx[IL][YF][YF] (Fig. 7*B*). For example, mutation of threonine at position 2 to any substitution abrogated inhibitory activity (Fig. 7*A*) suggesting a critical role for Threonine. Similarly, mutation Tyrosine at position 5 or position 6 was only tolerated by substitution to the bulky hydrophobic residues Phenylalanine and Tryptophan (Fig. 6*A*). Mutation of Isoleucine at position 4 was only tolerated by a Leucine substitution (Fig. 7*A*). By contrast, a large set of amino acid substitutions were tolerated at Proline position 1 or Threonine position 3 (Fig. 7*A*).

**Fig. 8.**
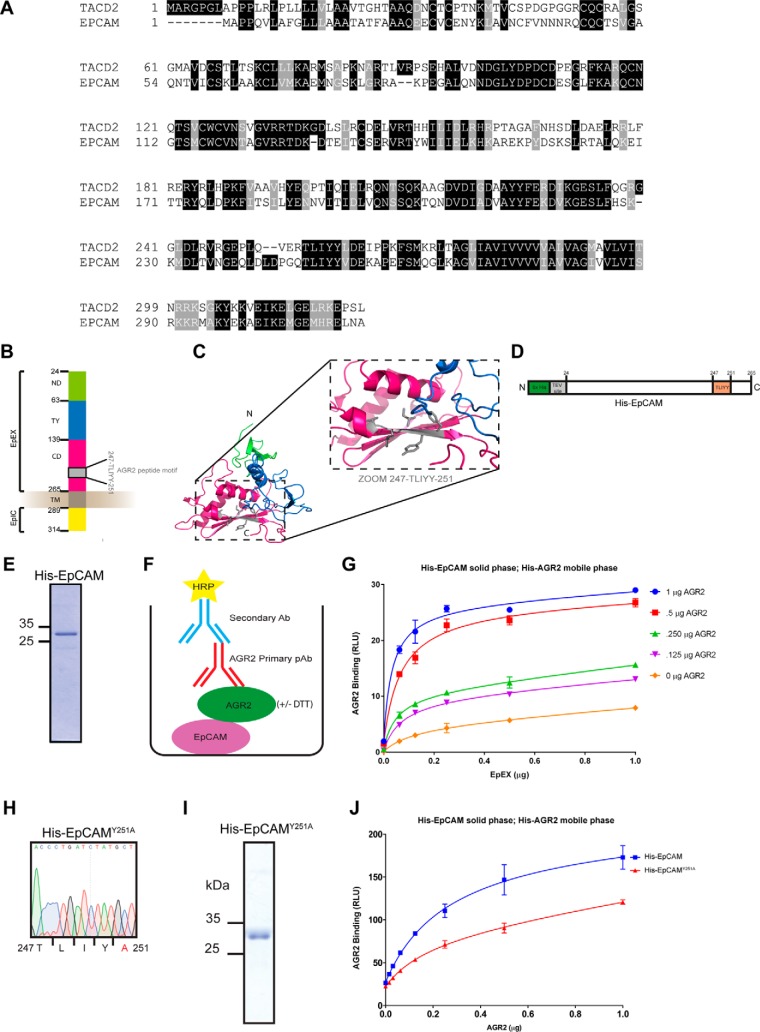
**EpCAM as a candidate AGR2 client protein.** *A*, Homology between EpCAM and its paralogue TACD2 as aligned using Clustal Omega. Both proteins were identified using *ScanProsite* (supplemental Table S1) and harbor the TLIYY motif implicated as an AGR2 linear peptide docking site. *B*, Secondary structure of EpCAM which consists of a N-domain (ND, green), Thyroglobulin type-1 domain (TY, blue) and C-domain (CD, dark pink) which altogether make up for extracellular domain (EpEX), transmembrane domain (TM, gray), intracellular domain (EpIC, yellow), and the amino acids from 247–251 containing the sequence TLIYY. *C*, Three-dimensional cartoon representation of extracellular part of human EpCAM (PDB code: 4MZ) highlighting the AGR2 linear peptide motif at amino acid position Thr247 to Tyr251 9 (gray). Color coding is the same as in (*B*). *D*, Schematic representation of his-tagged EpCAM protein sequence highlighting the extracellular domain (EpEX), the TEV cleavage site, and the TLIYY motif. *E*, Coomassie Blue staining of purified His-EpCAM showing the major band at 32 kDa under reducing SDS-PAGE condition. *F–G*, Solid-phase binding assay to measure AGR2 binding to EpCAM protein. Increasing amounts of EpCAM were immobilized on the surface of a microtiter plate (0–1 μg). AGR2 (0–1 μg) was titrated in the mobile phase and AGR2 binding to immobilized EpCAM was quantified using AGR2 specific antibody. The binding of AGR2 is plotted as the extent of protein-protein complex formation as RLU as a function of increasing protein in the mobile phase. *H,* DNA sequencing chromatogram traces of EpCAM^Y251A^. *I*, Coomassie Blue staining of purified His- EpCAM^Y251A^ mutant protein showing the major band at 32 kDa under denaturing SDS-PAGE. *J*, His-EpCAM or His-EpCAM^Y251A^ (1 μg) was immobilized onto the well surface of a microtiter plate as in (*F*) His-AGR2 WT (0–1 μg) was titrated in the mobile phase. AGR2 binding to immobilized EpCAM was quantified using AGR2 specific antibody. The binding is plotted as the extent of protein-protein complex formation as RLU as a function of increasing protein in the mobile phase.

Using the optimized stringent consensus site Tx[IL][YF][YF], *Scanprosite* was used to screen for motifs in the human proteome to identify potential human proteins that harbor the binding motif (Fig. 7*B*). Interestingly out of 409 proteins, over 40% were predicted to be membrane associated (Fig. 7*C*–7*D*). We therefore focused on validating a Tx[IL][YF][YF] motif-containing transmembrane receptor in this list (Fig. 7*D* and supplemental Tables S1 and S2) that also possesses oncogenic activity in order to identify a model protein that could validate the role of this linear motif on AGR2 binding and oncogenic capacity. Molecular functions and biological processes of such targets are listed in Figs. 7*E* and 7*F. SLiMSEARCH*4 was also used as a linear motif discovery tool in order to identify proteins harboring the consensus peptide-binding motif (supplemental Table S3). Cellular components enriched are associated with the ER + Golgi apparatus (14.9%) and exosomes (19.4%) (Fig. 7*G*). The overlap in proteins identified between the two search engines is shown in Fig. 7*H*. The receptor EpCAM is commonly identified between both searches. EpCAM contains a single TLIYY motif (Fig. 7*D*) as does its paralogue TACD2 (Fig. 8*A*). The linear motif is located within a structural domain adjacent to the extracellular stalk near the plasma membrane (Fig. 8*B* and 8*C*). We focus in this study on evaluating whether the TLIYY docking site on EpCAM plays a role in AGR2 binding *in vitro* and in cells. EpCAM is a relevant potential client protein of AGR2 because it is independently over-expressed in cancers, used as a targeted therapy with on-going clinical trials, and it's a circulating tumor biomarker(40).

##### Validating the Receptor EpCAM as a Specific AGR2 Interacting Protein

Recombinant AGR2 (Fig. 3*A* and 3*B*) and the extracellular domain of EpCAM (Fig. 8*D* and 8*E*) were purified from bacterial expression systems. The AGR2 protein was the mature isoform without its hydrophobic N-terminal leader sequence and the EpCAM protein retained its extracellular domain (amino acids 24–265) containing the TLIYY consensus motif (Fig. 8*D*). A dose-dependent titration of AGR2 in the liquid phase with increasing EpCAM protein on the solid phase (Fig. 8*F*) demonstrated that the proteins form stable protein-protein interactions using ELISA (Fig. 8*G*). An EpCAM mutant was constructed in which the Tyrosine-251 was mutated to alanine to create the TLIYA motif in EpCAM (Fig. 8*H* and 8*I*). The Y-A mutation at position 6 can attenuate, but not abrogate, AGR2 binding to the synthetic hexapeptides (Fig. 7*A*). A titration of AGR2 against the wt-EpCAM or EpCAM^Y251A^ demonstrated that AGR2 binding was also attenuated on EpCAM^Y251A^ mutant (Fig. 8*J*). These data highlight at least one binding site on EpCAM for AGR2 protein.

**Fig. 9.**
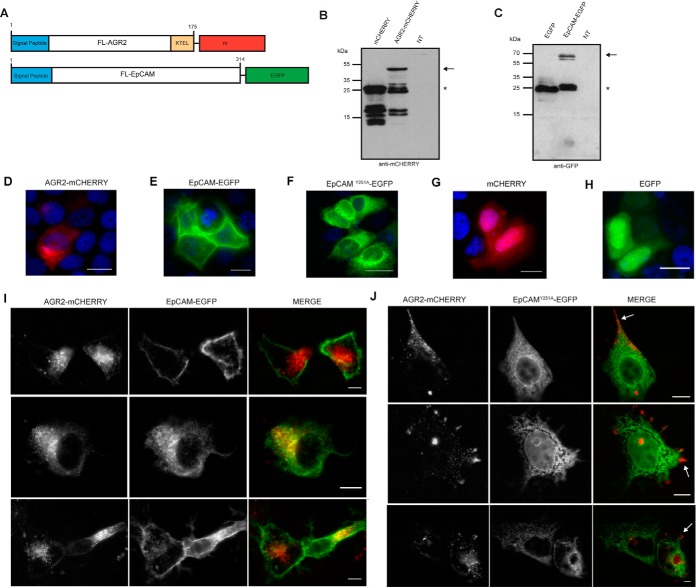
**Effects of Y251A mutation on EpCAM localization in cells.** *A–C*, Fluorescently labeled versions of AGR2 (mCHERRY) and EpCAM (EGFP) with the signal peptides were generated and expression validated in cells using immunoblotting (*B* and *C*). *B*, The immunoblotting of mCHERRY and mCHERRY-AGR2 transfected cells highlights the expression of mCHERRY alone (lane 1) and mCHERRY-AGR2 (lane 2). Blots were incubated with an anti-mCHERRY antibody. The arrow marks the location of full-length mCHERRY-AGR2 and the asterisk marks the location of mCHERRY. We noticed the reproducible small molecular mass “cleavage” or synthesis products when mCHERRY was transfected into cells. *C*, The immunoblotting of EGFP and EGFP-EpCAM transfected cells highlights the expression of EGFP alone (lane 1) and EGFP-EpCAM (lane 2). Blots were incubated with a GFP antibody. The arrow marks the location of full-length EGFP-EpCAM and the asterisk mark the location of EGFP. The EGFP was not subjected to the production of smaller molecular mass adducts as was the mCHERRY protein. Fluorescent microscopy was used to measure the relative localization of the following proteins; *D*, mCHERRY-AGR2 *E*, EGFP-EpCAM *F*, EGFP-EpCAM^Y251A^ *G*, mCHERRY and *H*, EGFP. *I* and *J*, The impact of cotransfection of mCHERRY-AGR2 and EGFP-EpCAM or EGFP-EpCAM^Y251A^ on their respective localizations. Representative images of the wild-type and mutant EpCAM, as well as AGR2, are highlighted in both panels. The arrow in J highlights AGR2 mislocalization to the plasma membrane periphery in EpCAM mutant cotransfections that mirrors the mutant EpCAM mislocalization to the nuclear membrane in the same cells.

**Fig. 10.**
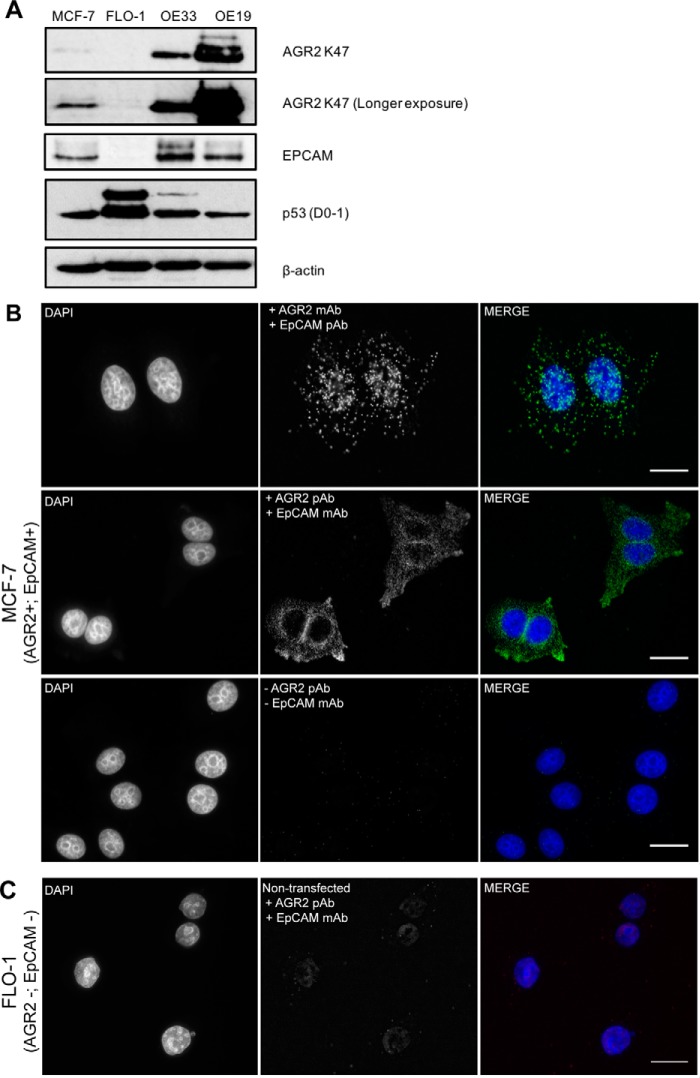
**Developing Proximity Ligation Models to define AGR2-EpCAM localization in cells.** *A*, Expression of AGR2 and its binding partner EpCAM in a panel of cancer cell lines. Breast cancer cell line (MCF-7) and esophageal cancer cell line (FLO-1, OE33, and OE19) was analyzed by Western blot using AGR2 polyclonal antibody K47 and EpCAM monoclonal antibody. Tumor suppressor protein p53 status was also analyzed p53 monoclonal antibody. β-actin was used as loading control. *B*, Representative image of a proximity ligation assay performed with antibody pair of AGR2 mouse monoclonal antibody and EpCAM rabbit polyclonal antibody (upper panel) or AGR2 rabbit polyclonal antibody and EpCAM mouse monoclonal antibody Ab (middle panel) in MCF-7 cells. PLA probes (Anti-rabbit PLUS probe and anti-mouse MINUS probe) were then added to the samples. Following ligation and amplification, protein-protein interaction complex was detected with green fluorescent probes (Duolink). Green fluorescence foci indicate the interaction between the two proteins. As a control, MCF-7 was not incubated with the antibody pair but incubated with proximity ligation assay probes (lower panel) that showed no or few foci. Scale bar 25 μm. *C*, As a negative control, proximity ligation assay also was also performed in cells that do not express AGR2 and EpCAM (FLO-1). AGR2 mouse monoclonal antibody and EpCAM rabbit polyclonal antibody was used to show that non-transfected FLO-1 demonstrated no significant amount of foci. Scale bar 10 μm. Nuclei were counterstained with DAPI and cells were visualized with an epifluorescence microscope.

Prior to continued validation of the AGR2-EpCAM protein-protein interaction, we next evaluated whether the TLIYY motif in EpCAM plays a role in its localization and/or binding to AGR2 in MCF7 cells. If so, this would place more significance on its' physiological relevance for further study. This MCF7 cell line expresses endogenous AGR2 and EpCAM (Fig. 10*A*) and we thought it important to use such a cell that has a physiological and active AGR2 and EpCAM expression system. Transfection of mCHERRY-AGR2 (Fig. 9*A* and 9*B*) and EGFP-EpCAM (Fig. 9*A* and 9*C*) proteins into cells confirmed that AGR2 localization is cytosolic (Fig. 9*D*) whereas EpCAM is largely plasma membrane bound (Fig. 9*E*). The mCHERRY controls and EGFP controls are shown in Fig. 9*G* and 9*H*, respectively, and exhibit distribution throughout the cell.

On cotransfection of mCHERRY-AGR2 and EGFP-EpCAM, a degree of colocalization of the two proteins is observed in the cytosol (representative widefield fluorescent images in Fig. 9*I*), suggesting that the two proteins can partially form stable interactions *in vivo*. The three types of distributions patterns observed (Fig. 9*I*) include: those with largely membrane bound EGFP-EpCAM and cytosolic mCHERRY-AGR2 without regions of apparent colocalization (top panel), a high degree of colocalization in the cytosol (middle panel), and a mixture of colocalization with some membrane bound EGFP-EpCAM (bottom panel). By contrast to wt-EGFP-EpCAM, the mutant EGFP-EpCAM^Y251A^ mutant was not expressed at the plasma membrane when expressed in MCF7 cells (Fig. 9*F*). On mCHERRY-AGR2 cotransfection, the EGFP-EpCAM^Y251A^ mutant remained localized predominantly to the cytosol and/or nuclear membrane and did not exhibit the same distribution as wt-EGFP-EpCAM (Fig. 9*J*). However, mCHERRY-AGR2 was, interestingly, mislocalized in EGFP-EpCAM^Y251A^ mutant cotransfected cells and was distributed largely to the periphery of the cell (Fig. 9*I*). These data suggest that this protein-protein interaction pair has impacts on each other in cell systems. Thus, we conclude from the cell-based validation that, although that there is not a strikingly “stable” colocalization of mCHERRY-AGR2 and EGFP-EpCAM in every cotransfected cell, the key results are: (1) a degree of “transient” colocalization of mCHERRY-AGR2 and EGFP-EpCAM and (2) that the EGFP-EpCAM^Y251A^ mutant is mislocalized suggesting that the AGR2 docking site is important, in cells, for the appropriate trafficking of EpCAM to its destination.

The cell-based experiment evaluating the binding of AGR2 to EpCAM (Fig. 9) used GFP or mCHERRY-tagged proteins. Using this methodology, EGFP-EpCAM can localize to the plasma membrane, where it has been published to reside (41). These data indicate that the GFP-tag does not preclude membrane localization of EpCAM. The advantage of this GFP-tag expression assay was that we could also mutate EpCAM at Y251 to create an attenuated AGR2-interacting mutant that in turn demonstrated its' mislocalization in cells. The disadvantage of the GFP-tag expression methodology is that it relies on using tagged proteins that might alter protein functions. As such we also aimed to evaluate whether endogenous, authentically expressed AGR2 and EpCAM can form protein-protein interactions *in situ* using Proximity Ligation assays (Fig. 10). Proximity ligations are emerging methodologies that have been shown to demonstrate the “association” of two proteins in cells (42, 43). Proximity ligation assays can identify a protein-protein association with a distance of 10–30 nm that is in the upper range of that observed using FRET (5–20 nm) and this methodology can detect authentic endogenous proteins *in situ* and does not rely on transfected or artificially GFP-tagged protein vectors (35, 44). It is important to highlight that the proximity ligation method does not prove a direct protein-protein interaction between two proteins, but it identifies an “association” within the fixed radius of two antibodies binding to two different epitopes in proximity of a distance constrained by the length of the oligonucleotides conjugated to two different secondary antibodies.

To study whether the method can detect an association of AGR2 and EpCAM, we required the use of cancer cell lines where AGR2 and EpCAM show mutual expression and where their assembly pathways are presumably intact. In screening for cell lines that contain AGR2 and EpCAM (Fig. 10*A*), we focused on the use of MCF7 cells because they express both proteins and have a wt-p53 pathway allowing for future impacts of AGR2 on p53 activity (27). Proximity Ligation assays were performed in MCF7 cells (AGR2+/EpCAM+) where the cells were incubated with different antibodies to the two proteins. We tested two sets of AGR2 and EpCAM antibodies. The first pair was an AGR2 mouse monoclonal antibody and an EpCAM rabbit polyclonal monoclonal (Fig. 10*B*, top panel). The merged data reveal cytosolic foci for the two proteins indicative of a protein-protein interaction. The second antibody set included an AGR2 rabbit polyclonal antibody and an EpCAM mouse monoclonal antibody (Fig. 10*B*, middle panel). The merged data also revealed significant cytosolic protein-protein interaction foci. As a control, we also incubated MCF-7 cells with PLA probes without antibodies, and these showed no or few foci (Fig. 10*B*, lower panel). These data provide evidence that endogenously expressed AGR2 and EpCAM can interact in cells and is consistent with the fluorescently tagged expression assays (Fig. 9). Proximity Ligation assays were also performed in FLO-1 cells that do not express AGR2 and EpCAM (Fig. 10*A*). This was done as another control for the Proximity Ligation assay to determine whether the antibody signals observed are dependent on the presence of AGR2 and EpCAM. When non-transfected FLO-1 cells were incubated with AGR2 and EpCAM antibodies there was no significant foci formation (Fig. 10*C*). There are no significant AGR2-EpCAM protein-protein association foci lining the plasma membrane in either MCF-7 cells, suggesting that AGR2 interactions are confined to prior events in the maturation of the EpCAM receptor, within the cytoplasm. This is consistent with the confocal microscopy using GFP and mCHERRY tagged proteins that also suggested a non-plasma membrane colocalization (Fig. 9). These data together highlight that authentic AGR2 and EpCAM form protein-protein associations in cells and further affirms that the “linear motif” screen using the TTIYY peptide (Fig. 7) could be used to identify a novel, physiologically relevant protein-protein interaction for AGR2.

##### Mutation of the Peptide-docking Site On AGR2 Induced a Gain-Of-Function Activity in EpCAM Protein Binding

Given the data highlighting a cell-based protein-protein interaction in cells suggests a physiologically relevant protein interaction pair, we set out to further fine map the impact of mutagenesis on the AGR2 and EpCAM protein interactions. The peptide-binding AGR2 mutants (D132A, P133A, and S134A) that exhibit neutral or attenuated binding to the synthetic peptide A4 (containing the TTIYY motif) were evaluated in EpCAM protein binding to determine the effects of these mutations on the AGR2: EpCAM protein-protein interaction.

**Fig. 11.**
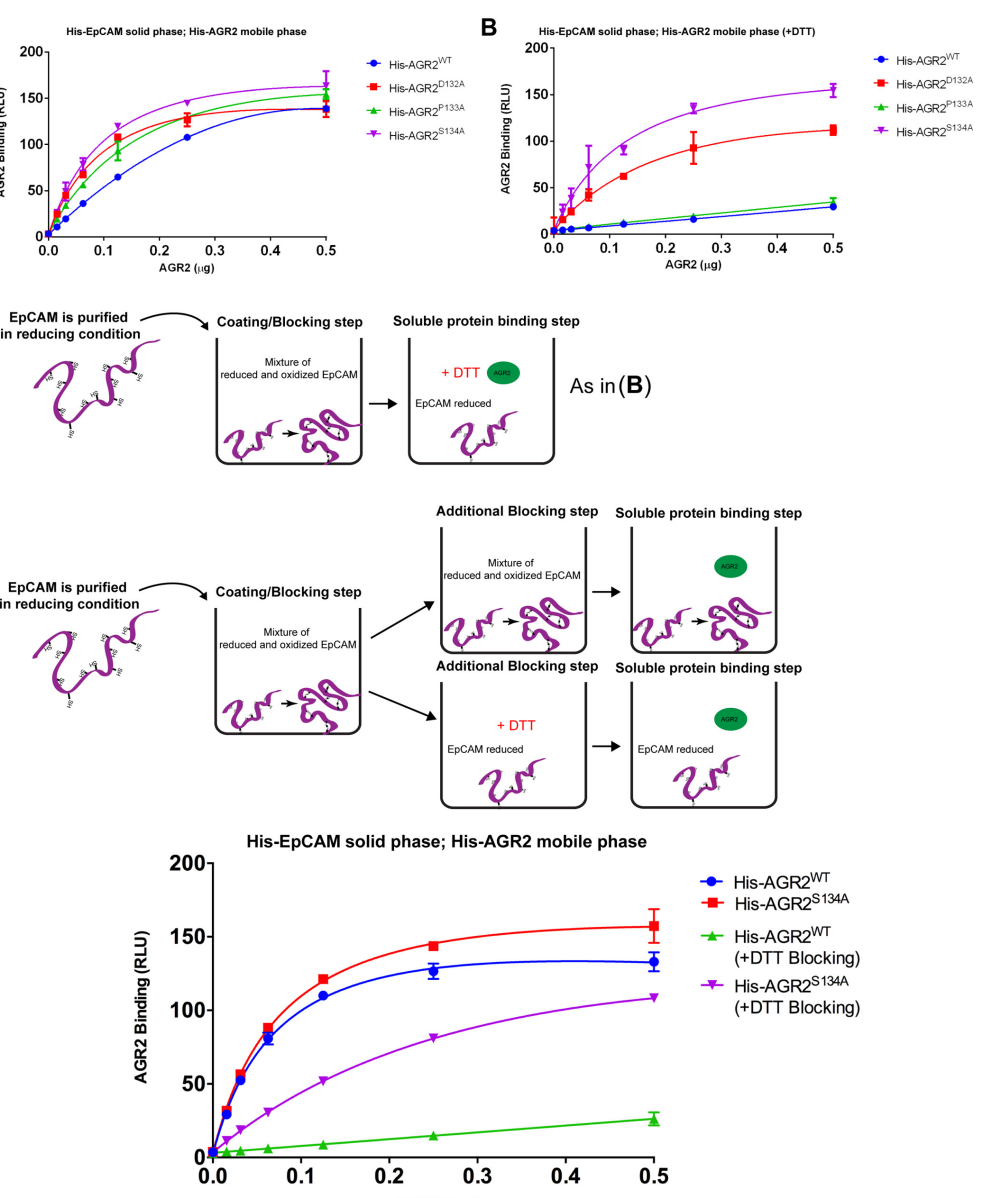
**Effect of AGR2 peptide docking mutants binding to EpCAM.** *A*, His-EpCAM was immobilized onto the well surface of a microtiter plate. Titration of AGR2 WT and mutants (0–0.5 μg) were added in mobile phase and AGR2 binding without (*A*) and with 1 mm DTT (*B*) to immobilized EpCAM was quantified using a specific AGR2 antibody. The binding is plotted as the extent of protein-protein complex formation in RLU as a function of increasing protein in the mobile phase. Reactions in (A) have no DTT included in the AGR2-binding reactions and (*B*) includes DTT at the stage of addition of AGR2 binding to reveal any effects of potential cysteine oxidation on protein-protein interactions (as highlighted in *C* where both AGR2 and EpCAM should be in the reduced state). *D,* Staging the effects of DTT by including reductant in the blocking step to determine whether the DTT effect (from *B*) on the reaction is because of EpCAM substrate and not AGR2 itself. *E*, A titration of wt-AGR2 and AGR2^S134A^ in the presence or absence of reductant in the blocking stage. As in (*A* and *B*), the binding is plotted as the extent of protein-protein complex formation in RLU as a function of increasing protein in the mobile phase.

The AGR2:EpCAM binding reaction revealed a gain-of-function of all three mutants, relative to wt-AGR2. AGR2^S134A^ exhibited the most elevated gain-of-function activity (Fig. 11*A*). As AGR2 has a thioredoxin domain that can form covalent bonds with client receptors such as mucins (15), it can be expected that we evaluate the impact of reductant on its biochemical function. For example, the AGR2^C81S^ mutant is as active as wt-AGR2 in stimulating *ex vivo* cell growth (12). The inclusion of DTT into reactions at the AGR2-binding stage (Fig. 11*B* and 11*C*), surprisingly, exacerbated the differences between the mutants and wt AGR2, with the S134A followed by the D132A mutants exhibiting the most pronounced gain-of-function activity (Fig. 11*B* and 11*C*).

We next staged this effect of reductant by including DTT only in the blocking stage, after EpCAM absorption, but before AGR2 binding (Fig. 11*D*). This would address whether this effect is because of the altered redox-imposed conformation of the substrate, EpCAM or the chaperone, AGR2. The incubation of DTT in the blocking step (Fig. 11*D*, upper panel) is the stage at which the effect of the S134A mutant of AGR2 shows the most dramatic difference compared with wt-AGR2 (Fig. 11*D*, lower panel). These data suggest that oxidized EpCAM, rather than AGR2, attenuates the impact of peptide docking site mutations on AGR2 functions. These data also suggest that the conformation of AGR2^S134A^ is altered to drive it toward an oxidation-sensitive binding site on EpCAM; *e.g.* this AGR2-binding site on EpCAM is apparently sensitive to EpCAM redox state. Lastly, these data suggest that the conformation of EpCAM can also have distinct impacts on the stable binding of AGR2 protein.

**Fig. 12.**
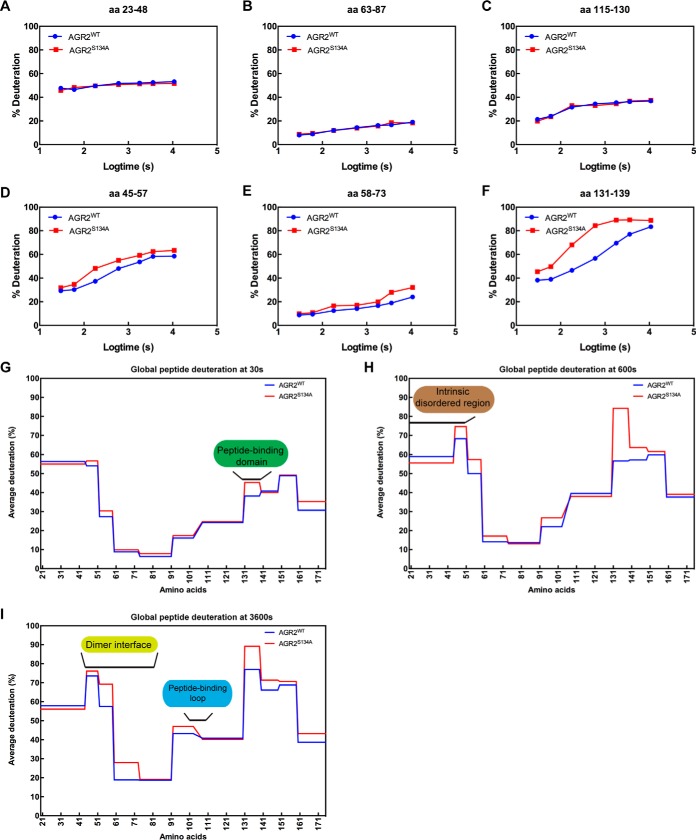
**Measuring conformational changes in the gain-of-function mutant AGR2S134A using hydrogen-deuterium mass spectrometry.** Wt-AGR2 or AGR2^S134A^ was deuterated over a 7-point time course from 30 s to 10,800 s followed by acidification, pepsinization, and separation of fragments using mass spectrometry (supplemental Fig. S3) *A–C*, Representative peptic ions of AGR2 protein that do not exhibit significant changes in deuteration between wt and mutant AGR2^S134A^ *D–F*, Representative peptic ions of AGR2 protein that do exhibit significant changes in deuteration between wt and mutant AGR2^S134A^. The data are plotted as % of deuteron exchange as a function of time (log10 in seconds; from 30, 60, 180, 600, 1800, 3600, and 10800). The deuterium exchange rates of individual peptides are summarized using the HDX exchange plots for (*G*) 30 s (*H*) 600 s, and (*I*) 3,600 s time course. In (*G*), we highlight that the most noticeable difference is enhanced deuteration at the “peptide binding domain”, including amino acids 131–135. However, at elevated times of deuteration (*H–I*), there is apparent exposure of the dimerization domain and the peptide-binding loop to solvent, suggestive of global conformational changes induced by the S134A mutation.

**Fig. 13.**
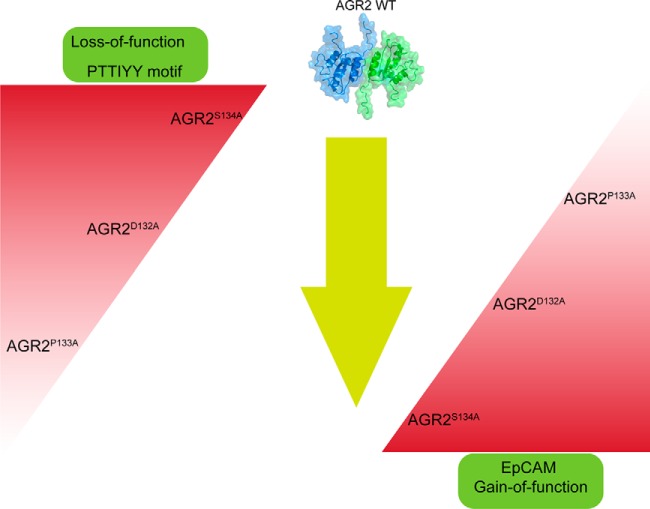
**Summary of the biochemical properties of the peptide-docking site mutations in AGR2 produced based on hydrogen-deuterium exchange mapping.** Based on the hydrogen-deuterium exchange mapping data (Fig. 4 and 5), we focused on creating three alanine substitutions mutations in the VDPSL loop motif, from amino acids 131–135, residing between two β-sheets (Fig. 6). The mutant proteins exhibit inverse trends in their specific activity. Consensus peptide binding reactions demonstrate that wt-AGR2 = S134A>D132A>P133A with two mutants showing a loss-of-function. Although in EpCAM binding, S134A>D132A>P133A = wt-AGR2 with two mutants showing a gain-of-function. These data suggest that although mutating some amino acids in the VDPSL motif can impact on specific peptide binding, the global conformation changed induced by loop mutation (for example in the gain-of-function S134A mutation (Fig. 11) might result in binding to a distinct site on the EpCAM molecule. Thus, one interpretation of such data is that one purpose of the VDPSL motif is to not only drive specific peptide binding by AGR2 but to constrain the conformational dynamics (or monomer-dimer equilibrium) of AGR2 so as to minimize its “binding to other sites” on its client proteins.

To evaluate whether AGR2^S134A^ does, in fact, have an altered conformation which is inferred from its-gain-of-function activity, hydrogen-deuterium exchange mass spectrometry was used to probe whether amide hydrogen-bonding deuterium exchange rates differ. Alterations in deuteration rates at amide bonds would infer an altered conformation of the mutated protein and/or its interaction with solvent. The wild-type and AGR2^S134A^ mutant proteins were diluted with D_2_O buffer and quenched from 30 s to 10,800 s post dilution (supplemental Figs. S5 and S6). Relative to wt-AGR2, there was enhanced deuteration at several regions throughout the mutated AGR2^S134A^ protein (Fig. 12*A*–12*F* for representative peptic fragments). The most pronounced changes were observed at the dimer interface (amino acids 51–71) and at the peptide-docking site (amino acids 131–141) (Fig. 12*G*–12*I*). For instance, the relatively selective and enhanced deuteration of the peptide-binding domain at the 30-s site point (Fig. 12*G*) suggests the region is intrinsically more solvent exposed, although this does not prevent binding of the AGR2^S134A^ mutant protein to the TTIYY peptide (Fig. 6). However, the increased deuteration at the dimer interface (minimal amino acids 60–64) at later time points (Fig. 12*H* and 12*I*) suggests that the AGR2^S134A^ mutation can impact on monomer-dimer equilibrium. Indeed, at early time points after deuteration, when the protein is diluted 10-fold into D_2_O, there is little difference in deuteration at the dimer interface. Thus, we would suggest that allosteric effects in the AGR2^S134A^ peptide-docking site could impact on the distal dimeric interface. For example, the 10-fold dilution of AGR2 (at a starting concentration of 1 μm before dilution with D_2_O and 0.1 μm after dilution) will take its final concentration lower than its published *K_d_* of 8.8 μm (7). Thus, such a dilution that is intrinsic to the deuterium exchange methodology will shift AGR2 into its monomeric state. As a consequence, in the ELISA reaction measuring EpCAM binding, the wt-AGR2 and AGR2^S134A^ proteins are diluted to a final concentration from 0.01 to 0.2 μm. Under these conditions, we would expect AGR2^S134A^ to be more conformationally altered and this might explain in part its gain-of-function activity toward EpCAM. Together, our biochemical data define a dominant peptide-binding pocket on AGR2 and highlight that mutation of this motif can impact on heterologous protein-protein interaction such as EpCAM protein (Fig. 13).

##### Mapping the Stable AGR2-binding Site on EpCAM

The key advances in this study included mapping the specific-peptide docking site on AGR2 *in vitro* and determining whether this specific peptide motif can be identified on putative AGR2-interacting proteins. We finally asked whether we can identify a role for the TLIYY motif in EpCAM on its specific interaction with AGR2 *in vitro*. Hydrogen-deuterium exchange mass spectrometry was performed on wt-EpCAM and the EpCAM^Y251A^ mutant to determine; (1) if AGR2 binds specifically to a particular site on EpCAM or whether it interacts non-specifically as could be generally expected for a molecular chaperone; (2) if AGR2 binds specifically, where this stable docking site might be located especially in relation to the TLIYY motif in EpCAM; and (3) how the Y251A mutation in EpCAM impacts on the specific or nonspecific interaction with AGR2.

**Fig. 14.**
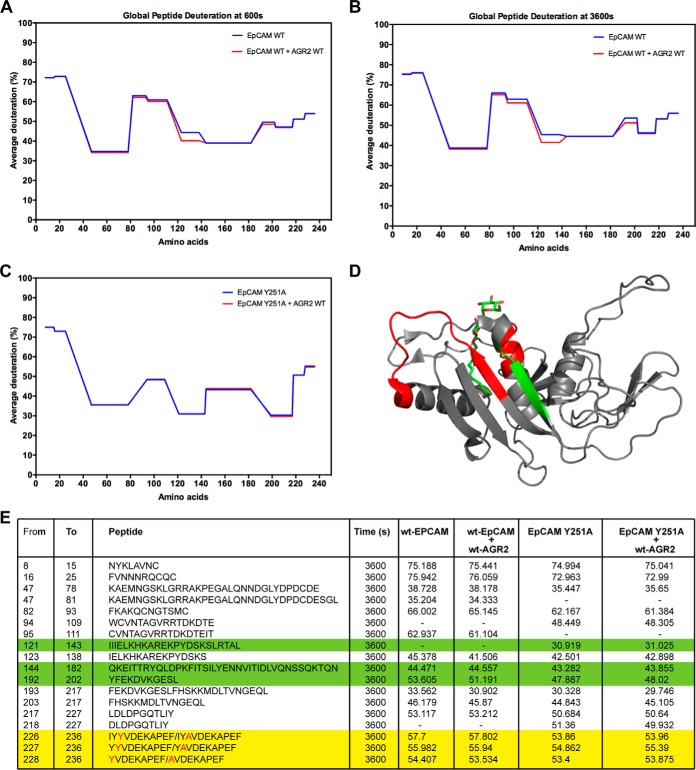
**Mapping the AGR2-binding site on EpCAM using hydrogen-deuterium exchange mass spectrometry.** The indicated wt or mutated (Y251A) versions of EpCAM were incubated for 60 min at room temperature with buffer only or AGR2 protein at a molar ratio of AGR2/EpCAM of 4:1. The proteins were then deuterated by dilution into deuterated buffer then incubated over a time course of up to 3600 s followed by reduction, acidification, pepsinization, and separation of fragments using mass spectrometry as indicated in the methods. The deuterium exchange rates of individual EpCAM peptides (supplemental Fig. S7) is summarized using the HDX exchange plots highlighting % deuteration as a function of amino acid position. The numbering of amino acids in the deuteration plots ranges from 1–241, in which the EpCAM used was from amino acids 24–265 creating a 24-amino acid stagger. *A*, wt-EpCAM deuteration alone or with AGR2 after 600 s; *B*, wt-EpCAM deuteration alone or with AGR2 after 3600 s; *C*, EpCAM^Y251A^ deuteration alone or with AGR2 after 3600 s; *D*, a summary of the key regions in EpCAM whose deuteration is suppressed by AGR2 (in red) based on EpCAM PDB (4MZV), and *E*, a table summarizing the key peptic peptides derived from wt or mutated EpCAM and how their deuteration changes as a function of Y251A mutation without or after stable AGR2 binding. In *D*, the detergent decyl-beta-d-maltopyranoside decylmaltoside (PDB 4MZV) is included in the image to highlight the proximity of the detergent binding domain to the stable AGR2-binding site. The green highlights the location of the TLIYY motif in the β-strand residing at the extreme C terminus of the recombinant EpCAM protein. In *E*, the yellow highlights the raw deuteration data of the overlapping TLIYY motif containing peptide that reveal no changes in deuteration after the 60 min preincubation allowing the complex to form between AGR2 and EpCAM. The pale green highlights the wt or mutated EpCAM peptic peptides identified that exhibit suppressed deuteration in the presence of AGR2 protein.

AGR2 protein or buffer control was preincubated with wt-EpCAM in a molar ratio of 4:1 for 60 min to allow stable protein-protein complex formation. Subsequently, the samples were slowly diluted 10x using deuterated buffer and incubated for various time points at which point reactions were acidified and processed for analysis by mass spectrometry (Supplemental Figs. 7–9). At the earlier time point of 600 s of incubation in deuterated buffer, there was selective suppression of deuteration of peptide fragments from amino acids 147–206 (Fig. 14*A*). Increasing time of deuteration to 3600 s resulted in maintenance of deuterium suppression from amino acids 147–206 with additional suppression of deuteration from aa 217–241 (Fig. 14*B*). These two regions form a discontinuous epitope in 2-dimensions but are proximal in 3-dimensions (Fig. 14*D*, in red). These two regions also map adjacent to the TLIYY motif (Fig. 14*D*, in green) and overlap the detergent (decyl-beta-d-maltopyranoside decylmaltoside) binding pocket (Fig. 14*D*, molecule imbedded from PDB code 4MZV). The mutant EpCAM^Y251A^ protein (verified for Y-A mutation in Supplemental Fig. 10) did not show stable interaction with AGR2 under these conditions (Fig. 14*C*). Higher AGR2/EpCAM protein ratios (greater than 4:1) resulted in general deuterium suppression over the majority of the EpCAM peptic fragments (data not shown), thus the ratio we used is optimized to capture the most dominant and specific AGR2 interaction sites on EpCAM protein.

Because we preincubate the AGR2 and EpCAM proteins for 60 min prior to processing, these data suggest that stable (*i.e.* equilibrium) binding of AGR2 occurs not at the TLIYY motif, but adjacent to the TLIYY motif and overlapping the hydrophobic pocket capable of crystallizing with the detergent. This also suggests the detergent binding pocket might also represent a protein-interaction site in EpCAM. Another interpretation of this data is that AGR2 “chaperonin” functions might alter the conformation of EpCAM near the TLIYY motif and then dissociate from EpCAM. Accordingly, the deuterium suppression observed (Fig. 14*B*) might relate to a conformational change in EpCAM and not reflect the direct stable binding site of AGR2 protein. Nevertheless, there appears to be a role for the TLIYY motif in the deuterium suppression on EpCAM, because AGR2 does not induce any detectable change in EpCAM^Y251A^ protein under these same conditions (Fig. 14*C*). This is consistent with the attenuated binding of AGR2 to EpCAM^Y251A^ protein using ELISA (Fig. 8). The data together suggest that the “weak” TLIYY docking site in EpCAM might form a “landing pad” that directs AGR2 into a more stable fit at adjacent motifs. This is reminiscent of previous mapping of a novel, “weak” or transient Nutlin-3 binding site on the rim of the hydrophobic pocket of MDM2 using hydrogen-deuterium exchange mass spectrometry. This latter data suggested that the weak Nutlin-3 nucleation site in MDM2 directs the molecule into the deep MDM2 pocket for a more stable fit at equilibrium (32).

## DISCUSSION

Discovering protein-protein interactions for a target is a key aim in life sciences(45) and is especially important where genetic screens are not available to define pathway functions. AGR2 exists only in vertebrate lineage thus limiting the possibility for developing genetic screens to define function. Thus, we need to rely on interactomics to expand on our knowledge of AGR2 function. Yeast-two hybrid methods have identified potentially important AGR2-binding proteins. The first yeast-two hybrid identified the membrane receptor and pro-metastatic protein Dystroglycan/C4.4a as an AGR2 interactor (18) and the newt homolog of AGR2 was shown to bind to the receptor Prod1 (8). These data are consistent with the emerging paradigm that AGR2 stimulates receptor maturation linked to growth or adhesion (46). Another yeast-two hybrid screen revealed that AGR2 could bind not only to membrane receptors but many nuclear proteins such as RIP140 and Reptin, which might reflect a role for the form of AGR2 that escapes the ER through its non-canonical KDEL retention sequence (47). The most well characterized AGR2 binding interactor is the AAA+ superfamily chaperone protein Reptin (20) (48) (23). However, perhaps the most intriguing biochemical function for AGR2 is in its intrinsic sequence-specific peptide-binding activity (24). In this report, we exploit this specific “peptide-binding” function of AGR2 to (1) propose a consensus-binding motif for its client proteins; (2) highlight that this motif is enriched in transmembrane proteins; and (3) characterize one such novel client protein, the oncogenic receptor EpCAM.

The majority of protein sequence information in higher eukaryotes is encoded by the “linear motif”; perhaps millions of such motifs are comprised of small stretches of amino acids that drive relatively weak but specific protein-protein interactions (49). A powerful tool to define the linear motif repertoire for a protein is the peptide-phage combinatorial library (50). This methodology has been used to define the peptide consensus motif for the peptide binding groove of the oncoprotein MDM2 (25). This information stimulated the development of peptide mimetics that inhibit MDM2 *in vivo* (37) (51). This methodology has also been used to define PxxP consensus binding motifs for the transcriptional coactivator p300 (52). When AGR2 was subjected to a peptide-phage library screen, a pentapeptide docking motif emerged as TTIYY (24). There were two distinct peptides selected in this latter study, but we had only focused on the one with the TTIYY motif. The specificity of the TTIYY peptide in binding to its target in crude lysates was evaluated using affinity purification (Fig. 1). The TTIYY-containing peptide could be used to affinity purify AGR2 from cell lines (Fig. 1) or human tissue biopsies with a high specificity (24). This suggests that the peptide could act as a highly specific recognition motif for AGR2 target proteins. However, our data using fluorescence polarization assays indicate that the *K_d_* for AGR2 binding to free consensus site containing peptides in solution is in the range of 15–45 μm (data not shown), suggesting the interaction, though very specific, is also very weak. These data suggest that although AGR2 might bind specifically to such motifs, the affinity is weak enough to allow rapid association-dissociation events that would presumably be important for its chaperone cycle in cells.

The human proteome was scanned for proteins containing this motif to produce a set of potential AGR2-client proteins and there was a relative enrichment in transmembrane proteins (Fig. 7). EpCAM was chosen as the target of choice and its paralog also shows identity in this region (Fig. 8*A*). The consensus motif within EpCAM forms a β-strand within the main α-β fold in the EpCAM structure and is adjacent to a detergent binding pocket, defined by PDB (4MZV). EpCAM mutation at Y251 prevents the receptor from reaching its normal destination at the plasma membrane (Fig. 9) and this mutation also attenuates stable AGR2-binding as defined using hydrogen-deuterium exchange mass spectrometry (Fig. 14). It has been noted that linear motifs can reside within intrinsically disordered domains or they can reside within structural domains (53). In the case of the linear motif within disordered regions, it is known that they can acquire distinct secondary structures depending on the nature of the protein-interaction. If the linear motif resides within a structural domain, the protein-protein interaction presumably requires an alternate conformation at the target site. Using EpCAM as a model, we can speculate that AGR2 might interact with an unfolded version of EpCAM as the receptor is being assembled in the ER. Alternatively, AGR2 might interaction with the receptor as its conformation is altered especially as the peptide-docking site is near the single-pass transmembrane domain.

By defining a peptide consensus for AGR2 (Fig. 7*B*), we were able to search for additional human proteins that might harbor this motif. Two different search engines were used for this. Transmembrane proteins predominated using *ScanProsite* (Fig. 7*C*) whereas proteins within the Golgi/ER or exosomes particles predominant using *SLiMSEARCH4* (Fig. 7*G*). There is a degree of specificity in this peptide motif, as a prior selection of peptides from phage libraries to p300 identified PxxP motif proteins that predominate in transcription factors like SMAD4 and p53 (52). The implication of this data is that the specific docking site of AGR2 is used to interact with client proteins that enter the ER or through trafficking. We had also characterized another receptor that has the AGR2 consensus linear motif site; the transmembrane protein Meckelin-3 (TMEM67; Fig. 7*D*). Mutating one of the several AGR2 consensus motifs on MKS3 impaired its ability to be assembled into the plasma membrane (M. Lawrence, PhD thesis (University of Edinburgh), manuscript in preparation) providing the proof-of-concept that receptors other than EpCAM might exploit an AGR2 docking motif for polypeptide folding or maturation.

The EpCAM receptor was validated as a putative, novel AGR2 client protein. We can speculate that because the docking site is in the C terminus of EpCAM adjacent to the single pass transmembrane domain if AGR2 binds to this inside the ER, the motif could impact on how the protein folds as it enters the ER. Because stable binding exists adjacent to this TLIYY motif within a detergent binding site (Fig. 14), this might reflect a chaperonin binding domain for AGR2 as it directs EpCAM folding in cells. The docking site might direct AGR2 to EpCAM to allow the correct formation of key disulfide bonds. Alternatively, the docking site might facilitate AGR2-mediated transport of EpCAM cargo as it is transported in a partially folded state to the plasma membrane. A key observation is that in cells, the EpCAM-Y251A mutant protein fails to reach its normal plasma membrane destination but is present in the cytosol or nuclear membrane (Fig. 9). There is a redox component to this docking site *in vitro* (Fig. 11), suggesting that disulfide bridge formation in the EpCAM protein can impact on how AGR2 binds in cells, although cell-based di-sulfide shuttling assays might be more difficult to reconstitute in cell systems. Future cell-based assay developments can impact on understanding how the “detergent binding site” (Fig. 14) and/or the EpCAM docking site (amino acids 247–251) facilitates its maturation or trafficking. For example, it would be interesting to generate a gain-of-function AGR2-S134, cysteine-mutated EpCAM, or “detergent-binding domain” mutant EpCAM derived cell models using CRISPR gene editing to measure changes in the flux of EpCAM receptor maturation as a result of such mutations. A second key observation in cellular based assays is that untagged endogenously expressed EpCAM and AGR2 can colocalize in cells, as defined by Proximity Ligation assays. This method is a tool that improves on the immunoprecipitation method for validating or identifying interacting proteins because the proteins are analyzed *in situ* and the interaction is limited by distance constraints (61). The cytosolic association between AGR2 and EpCAM using proximity ligation (Fig. 10) is consistent with cytosolic interaction observed using GFP and mCHERRY tagged ectopically expressed proteins (Fig. 9).

Hydrogen-deuterium exchange mass spectrometry was applied to determine whether a specific binding site of the TTIYY containing peptide could be identified on AGR2. Hydrogen-deuterium exchange mass spectrometry is a powerful method to evaluate ligand binding to a target protein (54), protein-protein interaction sites (55) and effects of mutations on conformational dynamics (56). The methodology has been validated using proteins for nearly 20 years (57). Although the translation from mass spectrometric laboratories to cell biology fields has not been so widespread, new software might accelerate translation to the life sciences. The technical methodology uses certain assumptions that can impact on data interpretation. For example, the use of pepsin at low pH to fragment a “denatured,” deuterated protein into “random” overlapping polypeptides has limitations. As highlighted in Fig. 3, pepsin treatment of AGR2 does not produce a theoretically perfect overlapping peptic series, with “hotspots” of cleavage that reside proximal to particular functional domains. This suggests that there are significant secondary structural elements at low pH that can hinder pepsin cleavage, as the target protein is not fully denatured. Such an imperfect peptic product series reduces the extent of high-resolution fine-mapping of ligand binding effects (Fig. 5) and/or conformational effects of a missense mutation (Fig. 12). In our report, the most likely peptide A4-binding site on AGR2 was localized to amino acids 131–139 (Fig. 5) and on Nutlin-3 binding to MDM2 to broad regions within two distinct functional domains (Fig. 2). Under these conditions, hydrogen-deuterium exchange can be considered a low-resolution structural tool that identifies functional motifs. The method also has the advantage of requiring relatively small amounts of protein, although another limitation is that the target protein requires significant dilution with D_2_O that might impact on conformational dynamics. Nevertheless, the hydrogen-deuterium exchange data allowed for orthogonal approaches that validated using mutagenesis the role of amino acids 131–135 in AGR2-peptide binding (Fig. 11 and Fig. 12).

A small region from amino acids 131–135 was identified in AGR2 as a likely, primary binding site for TTIYY motif-containing peptides, because it is here that the most significant deuterium suppression was observed after peptide binding (Fig. 5). Additional residues C-terminal to the 131–135 amino acids motif, including RADITGRYSNRL, also show deuterium suppression by the consensus site peptide A4, however, this region was not evaluated by mutagenesis. We focused on amino acids 131–135 because this represents an exposed surface with an unstructured turn that might form a binding finger into hydrophobic TTIYY docking sites. To further validate this data, a set of mutants were created that exhibit a loss-of-function on synthetic peptides (Fig. 6), suggesting that this motif is the major peptide-docking site. By contrast, the same series of AGR2 mutant proteins exhibited an inverse gain-of-function activity on the client protein EpCAM (Fig. 11 and 12), whose interpretation is complicated because of the oxidation-dependent resilience of EpCAM to this AGR2 mutant series (Fig. 11). Nevertheless, the ability to create a loss or gain-of-function mutations in this region suggests that it not only provides a primary peptide- interaction site, but that the conformation of this region can allosterically affect distal regions that impact on monomer-dimer equilibrium. The enhanced binding of EpCAM to this S134A mutant also suggests that AGR2 might have a secondary binding site on EpCAM that is stabilized by the altered conformation of the S134A mutant (Fig. 12). This potential secondary site appears to play only a minor role on wt-AGR2 protein because the EpCAM^Y251A^ mutation attenuates wt-AGR2 binding (Fig. 8).

Another feature of the deuterated peptide data (for example, observed in supplemental Fig. S1, S3, S5, and S7) and in other studies using HDX Workbench (56) or HDExaminer (55) is that larger peptide fragments can have a different number of deuteron exchanges than predicted within smaller or partially overlapping fragments. For example, in AGR2 (supplemental Fig. S3), the peptic peptide 17–56 shows a 30% deuteration exchange (corresponding to 10.8 deuterons), the embedded peptide 17–30 shows 55% deuteration (corresponding to 7.1 deuterons), and the overlapping peptide 31–52 shows 50% deuteration (corresponding to 9.5 deuterons). These inconsistencies in the number of deuterons exchanged relative to the expected levels, within overlapping peptides of different lengths or within smaller peptides embedded within larger peptides highlights, putative, could be due many factors such as secondary structural constraints under the low pH of the peptic reactions (as suggested by Fig. 3) or by peptide length/charge state (58). In addition, it is also possible that high levels of deuterium suppression in a small stretch of the polypeptide are compensated by elevated deuteration in adjacent regions (because of conformational changes) so that the net deuteration in larger peptides is different than the smaller peptide. This is relevant to AGR2 because it is diluted 10-fold in D_2_O during the deuteration reaction, during which the AGR2 concentration is far lower than the *K_d_* (of dimerization) that could in turn impact on its monomer-dimer equilibrium. Alternatively, there could also be variability in peptide back exchange where it has been reported that higher-order structures in a polypeptide might reduce or elevate the rates of hydrogen-deuterium exchange depending on the peptide length and/or sequence. The degree of secondary structural effects through peptide-column interactions can also impact on exchange rates (59). Systematic studies on factors that can impact on back-exchange have identified an unexpected dependence on ionic strength (60). Considering these properties of the methodology, we can speculate that the linear gradient used in the separation of AGR2 peptic peptides might increase back-exchange on the column because they are eluted much later in the gradient than then their corresponding nested fragments. Despite these quantitative differences in expected deuteration rates because of the complexities of the methodology, the software can accurately quantify deuterium exchange rates using peptic peptides of the same length (sequence) but under different conditions (ligand or protein mutation; *e.g.* example peptides include Fig. 4*C* or 5*B*). With these limitations of the methodology, useful data can be acquired on ligand binding (as in Figs. 2 and 5) or on heterologous protein-protein interaction motifs (55).

To conclude, a methodology has been developed to exploit the linear peptide motif as a tool to discover new protein-protein interactions for a molecular chaperone. This includes (1) validating the specificity of a peptide(s) acquired from combinatorial peptide libraries as an affinity purification tool from crude cellular lysates (Fig. 1); (2) using hydrogen-deuterium exchange to demonstrate a specific interaction site for the peptide that allowed the creation of mutants for validation (Fig. 2 – 6); (3) using alanine scan mutagenesis to derive a linear motif consensus site (Fig. 7); and (4) validating the EpCAM-AGR2 protein interaction *in vitro* and in cells (Fig. 8 – 14). These *in vitro* and cell-based methodologies provide a complementing approach to the yeast-two-hybrid and immunoprecipitation methodology to identify and validate new protein-protein interactions by exploiting the linear peptide motif as a common type of protein-protein interaction that is involved in dynamic protein-protein assembly reactions (49). Our data highlight that the EpCAM receptor has specific AGR2 binding regions. The recent identification that EpCAM and AGR2 proteins are coexpressed at a very high frequency in human esophageal adenocarcinoma cancer biopsies (27) provides a clinical rationale to further dissect the AGR2-EpCAM pathway control in relation to carcinogenesis and potential therapeutics in this cancer type.

## DATA AVAILABILITY

The mass spectrometry proteomics data have been deposited to the ProteomeXchange Consortium via the PRIDE repository with the data set identifier PXD005782.

## Supplementary Material

Supplemental Data

## Footnotes

^1^ The abbreviations used are:

AGR2: anterior gradient 2
ER: endoplasmic reticulum
ECL: enhanced chemiluminescence.
