# Supplementary material for: The Sequence-specific Peptide-binding Activity of the Protein Sulfide Isomerase AGR2 Directs Its Stable Binding to the Oncogenic Receptor EpCAM
Source: Mol Cell Proteomics. 2018 Jan 16;17(4):737–63. doi: 10.1074/mcp.RA118.000573 (PMC5880107; doi:10.1074/mcp.RA118.000573)
Supplement: Supplemental Data [file supp_17_4_737__index.html]

The sequence-specific peptide-binding activity of the protein sulfide isomerase AGR2 directs its’ stable binding to the oncogenic receptor EpCAM — sequence-specific peptide docking by the AGR2 chaperone — The Sequence-specific Peptide-binding Activity of the Protein Sulfide Isomerase AGR2 Directs Its Stable Binding to the Oncogenic Receptor EpCAM — Sequence-specific Peptide Docking by the AGR2 Chaperone — Supplemental Data 

# The Sequence-specific Peptide-binding Activity of the Protein Sulfide Isomerase AGR2 Directs Its Stable Binding to the Oncogenic Receptor EpCAM

## Supplemental Data

- 1. Datasets summarizing MDM2 ligand binding effects on MDM2 conformation analyzed by hydrogen-deuterium exchange mass spectrometry. - data highlighting extent of deuteration of individual peptic peptides
- 2. Butterfly plots showing the effect of Nutlin-3 ligand on overall MDM2 deuteration changes - kinetics of MDM2 deuteration as a function of ligand
- 3. Datasets summarizing AGR2 ligand binding effects on AGR2 conformation analyzed by hydrogen-deuterium exchange mass spectrometry. - effects of TTIYY containing peptides on AGR2 deuteration
- 4. Butterfly plots showing the effect of peptide ligand on overall AGR2 deuteration changes - kinetics of deuteration of individual peptic peptides as a function of ligand
- 5. Datasets summarizing effects of the S134A mutation on the overall conformation of AGR2 protein analyzed by hydrogen-deuterium exchange mass spectrometry. - data highlighting extent of deuteration of individual peptic peptides
- 6. Butterfly plots showing the effect of S134A mutation on overall AGR2 deuteration changes - kinetics of agr2 deuteration as a function of mutation
- 7. Datasets summarizing the deuteration of EpCAM protein (wt and mutant) with buffer only or with AGR2 protein. - data highlighting extent of deuteration of individual peptic peptides
- 8. Butterfly plots showing the effect of Y251A EpCAM mutation on overall deuteration changes in the presence of AGR2 - kinetics of EpCAM deuteration as a function of mutation and AGR2 protein
- 9. Peptic coverage of both wt-EpCAM and Y251A-mutant EpCAM used for HDX analysis - details of peptide coverage after pepsinization
- 10. Sequence coverage and MS/MS spectrum of Y251A EpCAM mutant to demonstrate that the mutated EpCAM protein can be detected in the HDX reaction - Raw spectral data highlighting the Y-A mutation in EpCAM peptic fragments
- 1. Excel file of AGR2 linear peptide motif hits from ScanProsite database mining. - a list of potential AGR2 interacting proteins stratified by motif
- 2. Excel file of functional enrichment analysis using FunRich. - functional implications of target protein binding
- 3. Excel file of AGR2 linear peptide motif hits from SLIMSEARCH4 database mining - Using an independent database for identifying potential interacting proteins
- Supplementary figure legends - description of the individual supplementary files
