# Supplementary material for: The Sequence-specific Peptide-binding Activity of the Protein Sulfide Isomerase AGR2 Directs Its Stable Binding to the Oncogenic Receptor EpCAM
Source: Mol Cell Proteomics. 2018 Jan 16;17(4):737–63. doi: 10.1074/mcp.RA118.000573 (PMC5880107; doi:10.1074/mcp.RA118.000573)

2-17: CNTNMSVPTDGAVTTS (#1)

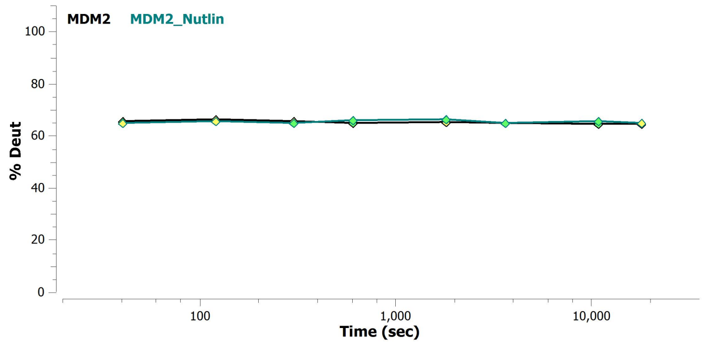

2-23: CNTNMSVPTDGAVTTSQIPASE (#2)

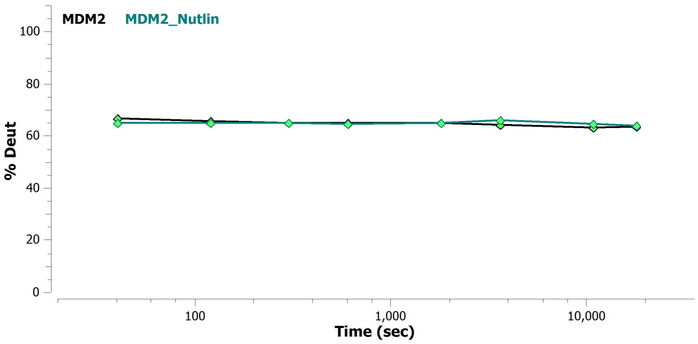

3-23: NTNMSVPTDGAVTTSQIPASE (#3)

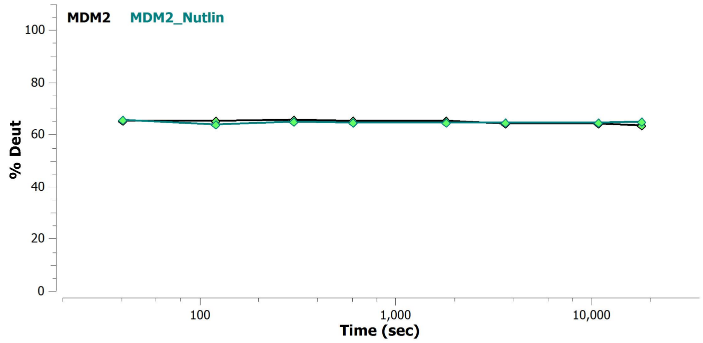

5-23: NMSVPTDGAVTTSQIPASE (#4)

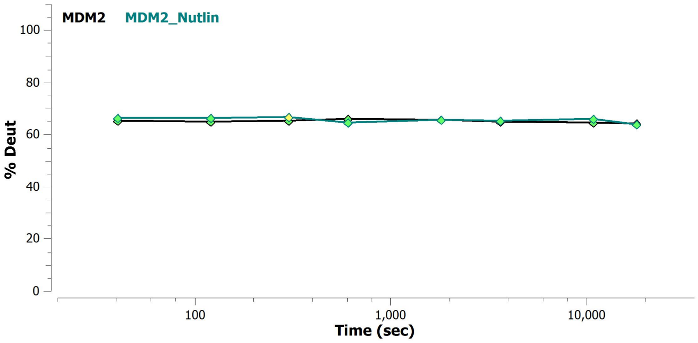

13-35: AVTTSQIPASEQETLVRPKPLLL (#5)

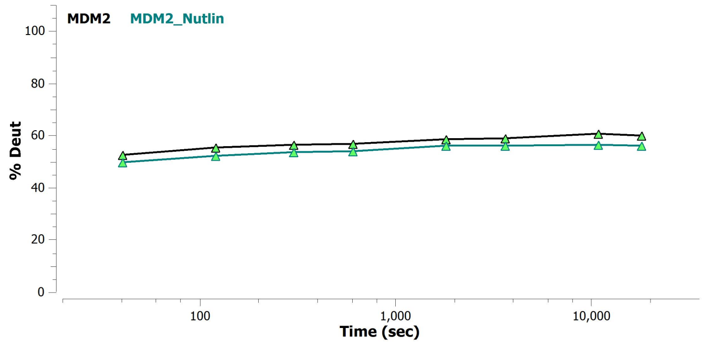

14-26: VTTSQIPASEQET (#6)

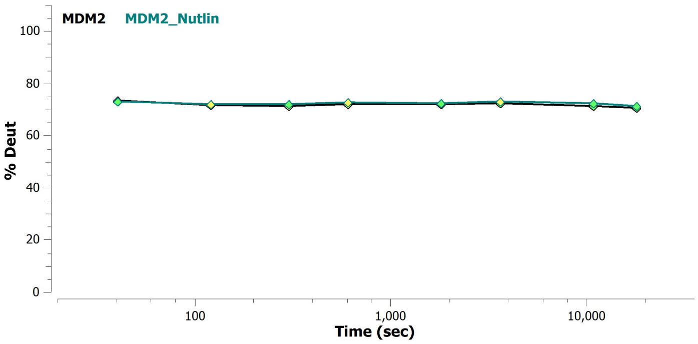

14-27: VTTSQIPASEQETL (#7)

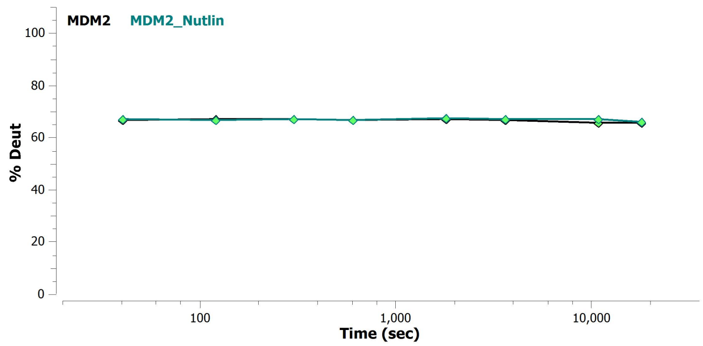

14-34: VTTSQIPASEQETLVRPKPLL (#8)

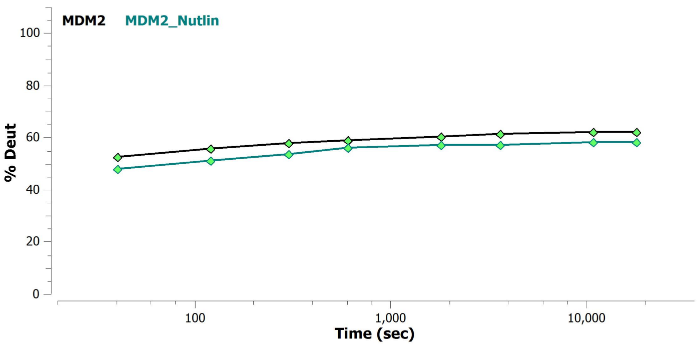

16-34: TSQIPASEQETLVRPKPLL (#9)

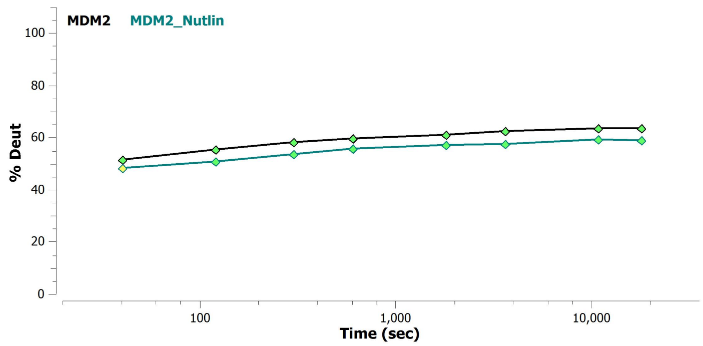

24-35: QETLVRPKPLLL (#10)

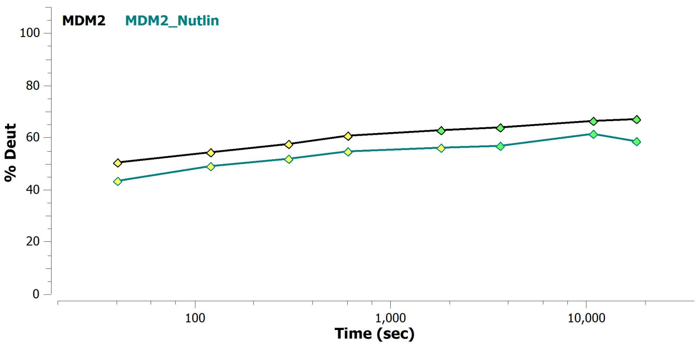

25-34: ETLVRPKPLL (#11)

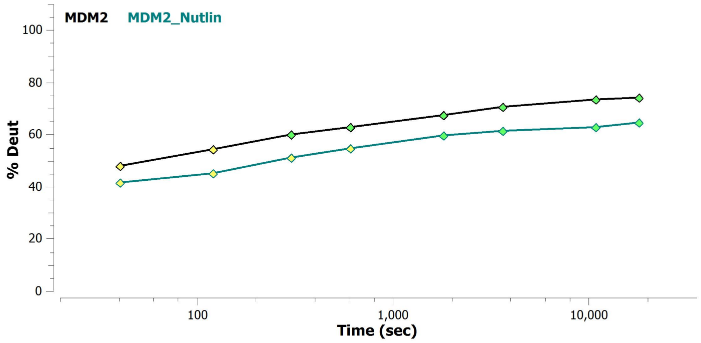

25-35: ETLVRPKPLLL (#12)

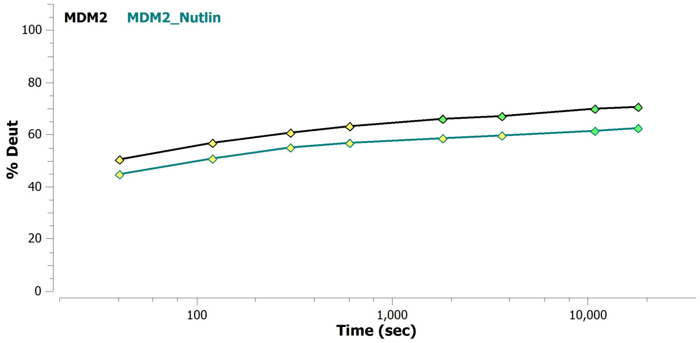

26-34: TLVRPKPLL (#13)

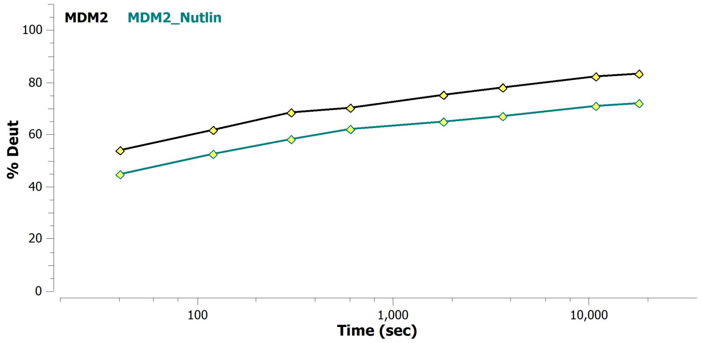

28-34: VRPKPLL (#14)

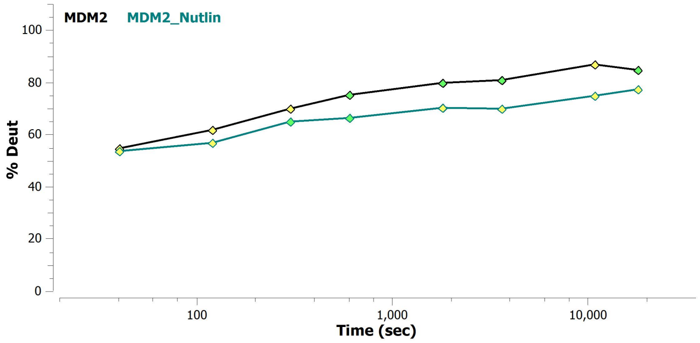

35-49: LKLLKSVGAQKDTYT (#15)

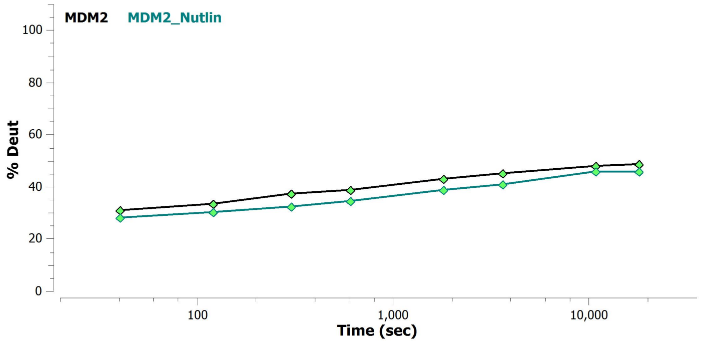

35-54: LKLLKSVGAQKDTYTMKEVL (#16)

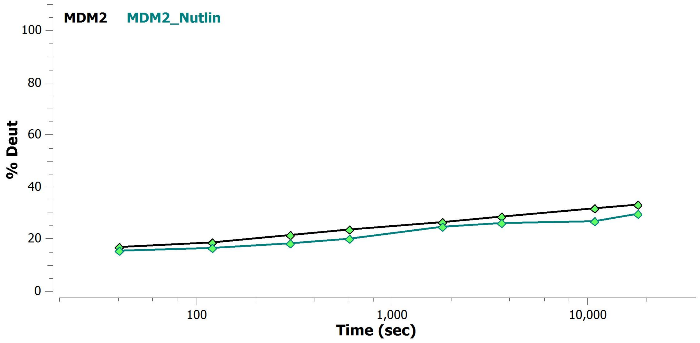

36-52: KLLKSVGAQKDTYTMKE (#17)

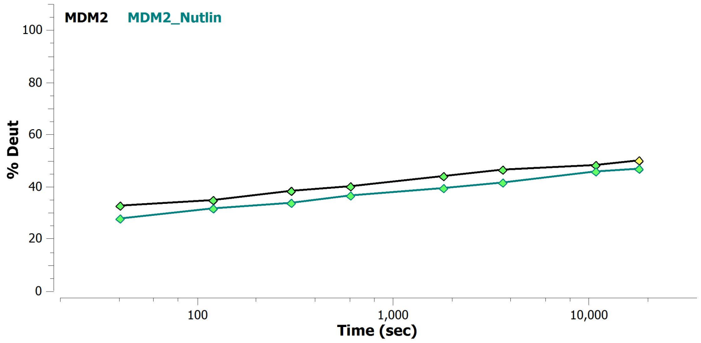

36-54: KLLKSVGAQKDTYTMKEVL (#18)

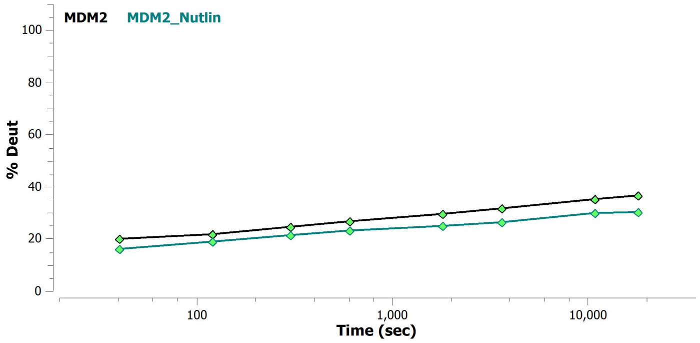

38-50: LKSVGAQKDTYTM (#19)

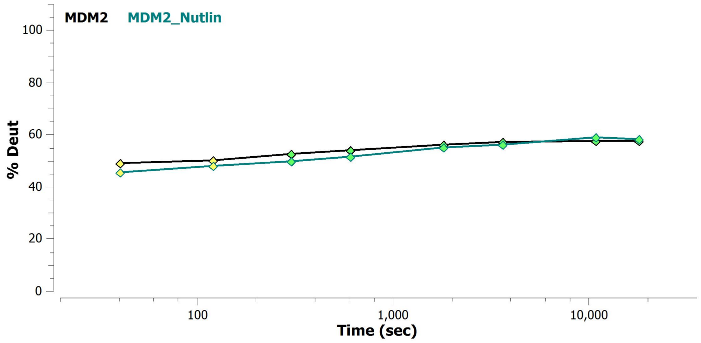

38-52: LKSVGAQKDTYTMKE (#20)

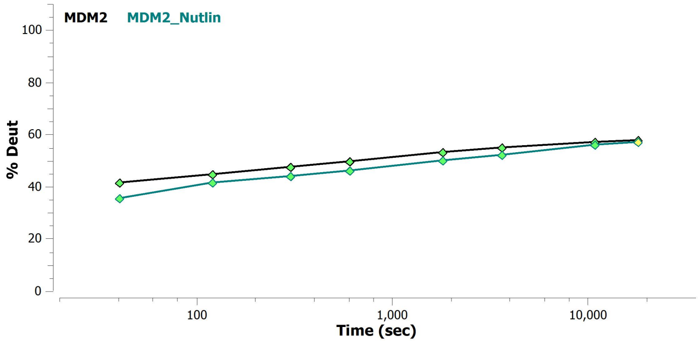

56-76: YLGQYIMTKRLYDEKQQHIVY (#21)

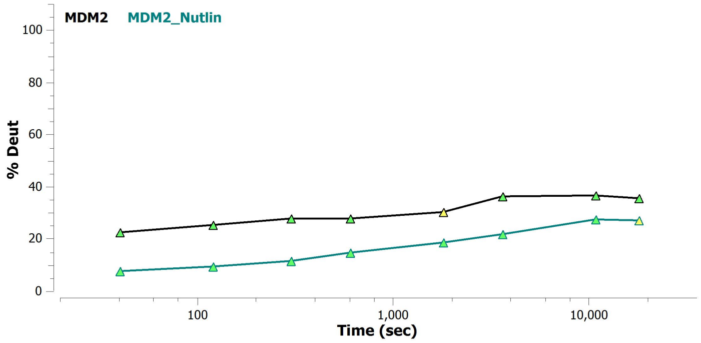

60-75: YIMTKRLYDEKQQHIV (#22)

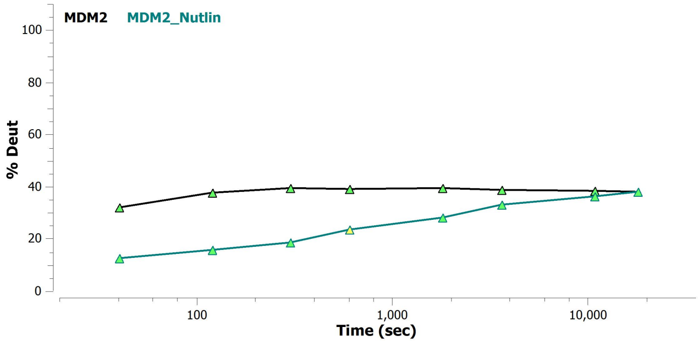

61-76: IMTKRLYDEKQQHIVY (#23)

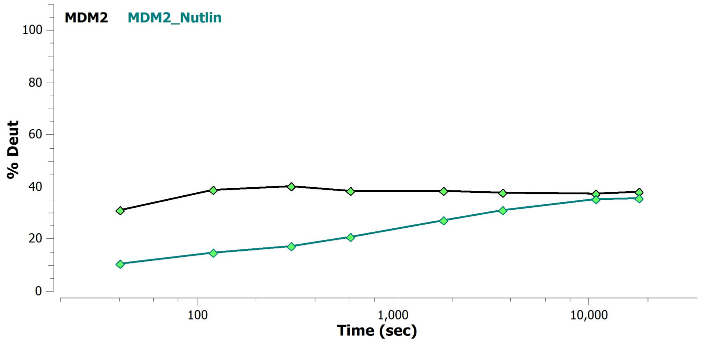

63-76: TKRLYDEKQQHIVY (#24)

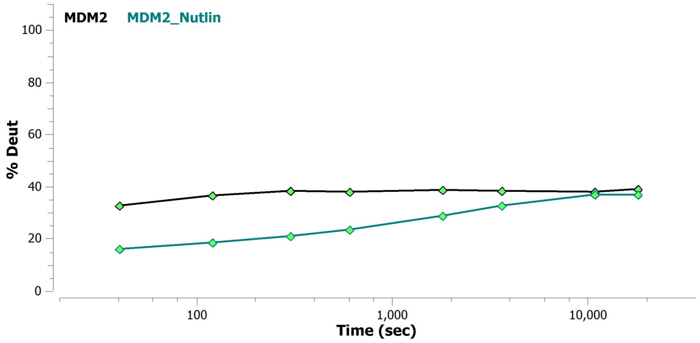

64-76: KRLYDEKQQHIVY (#25)

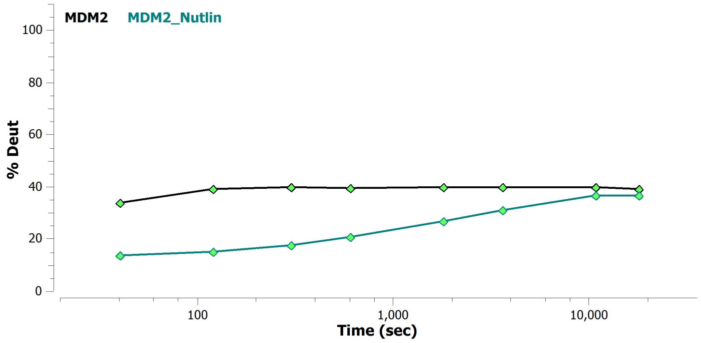

67-76: YDEKQQHIVY (#26)

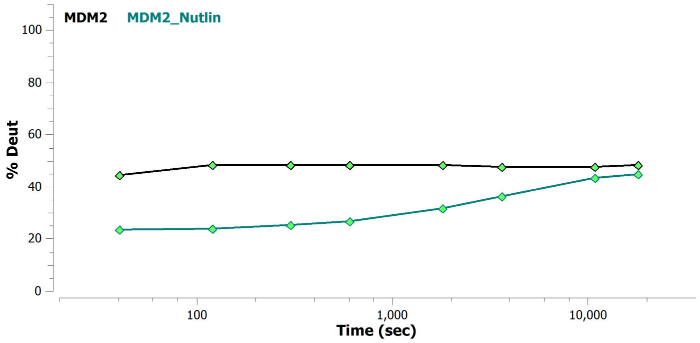

86-102: FGVPSFSVKEHRKIYTM (#27)

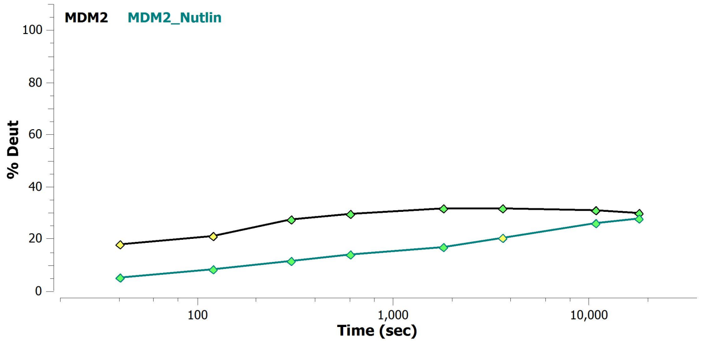

86-103: FGVPSFSVKEHRKIYTMI (#28)

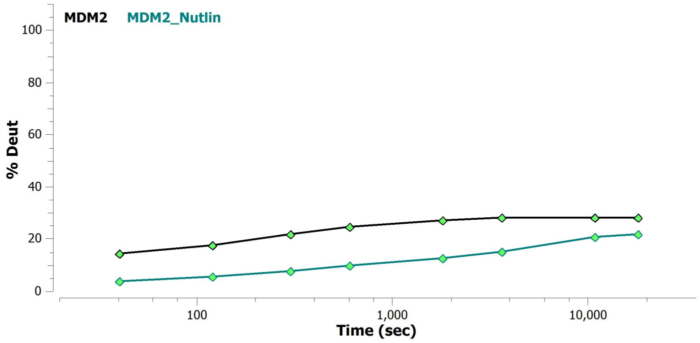

92-102: SVKEHRKIYTM (#29)

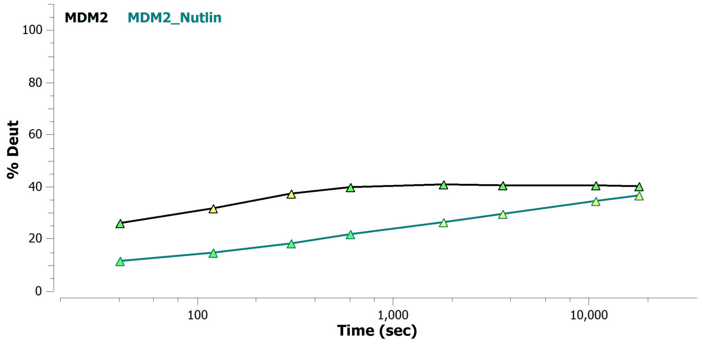

92-103: SVKEHRKIYTMI (#30)

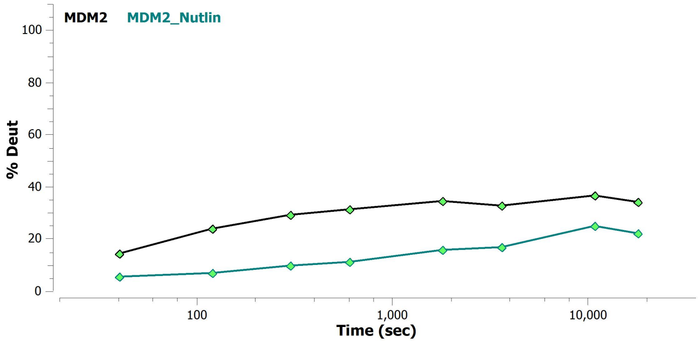

103-114: IYRNLVVVNQQE (#31)

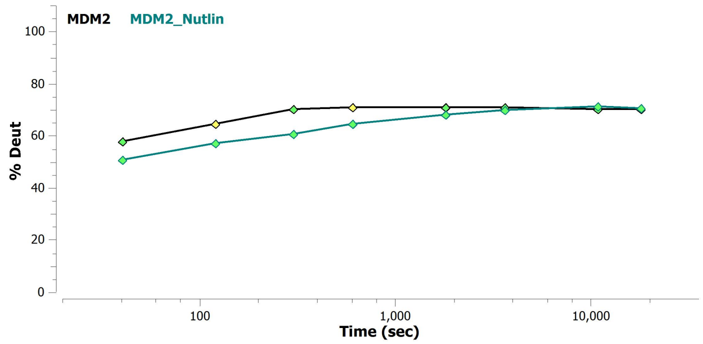

103-121: IYRNLVVVNQQESSDSGTS (#32)

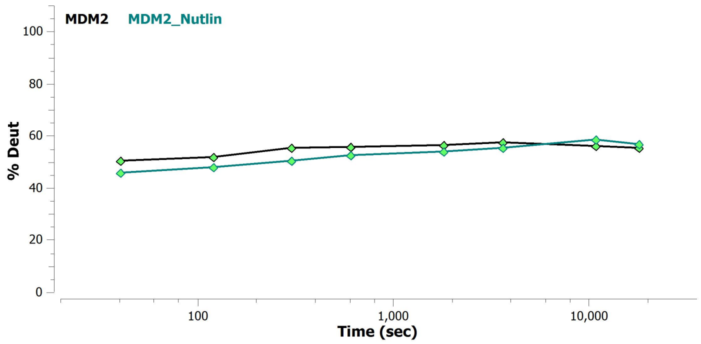

103-129: IYRNLVVVNQQESSDSGTSVSENRCHL (#33)

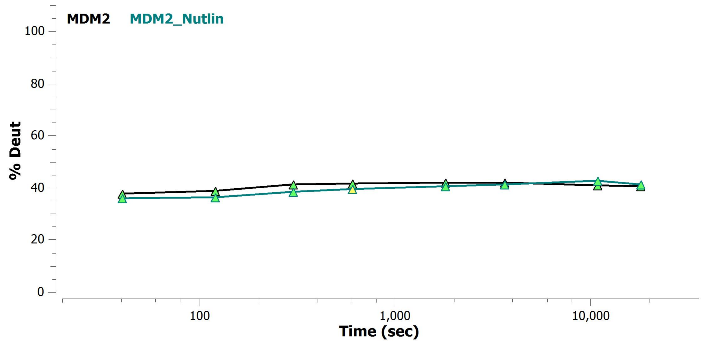

104-121: YRNLVVVNQQESSDSGTS (#34)

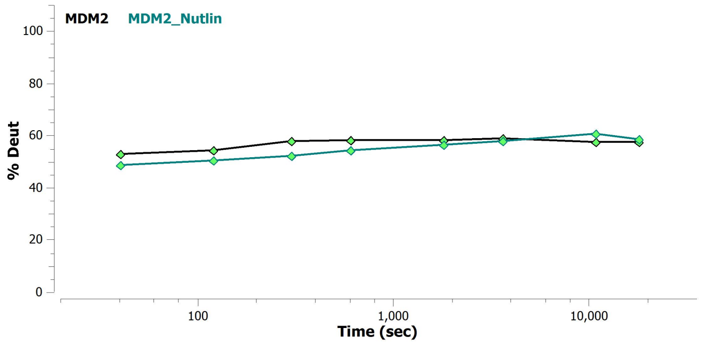

104-129: YRNLVVVNQQESSDSGTSVSENRCHL (#35)

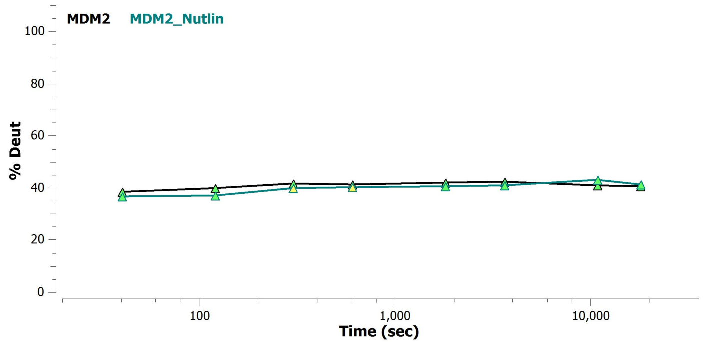

107-129: LVVVNQQESSDSGTSVSENRCHL (#36)

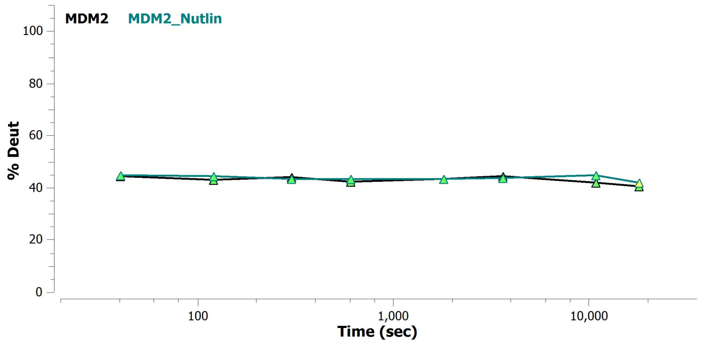

121-141: SVSENRCHLEGGSDQKDLVQE (#37)

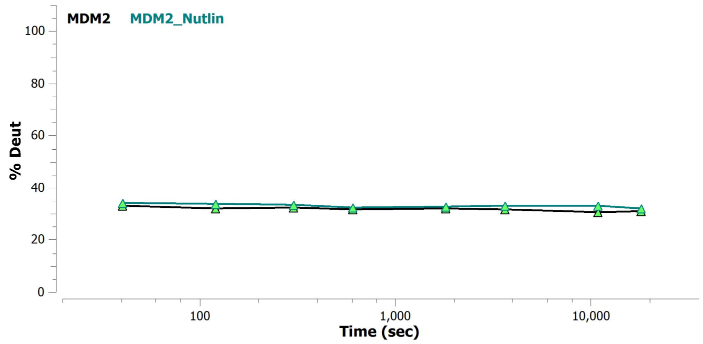

122-137: VSENRCHLEGGSDQKD (#38)

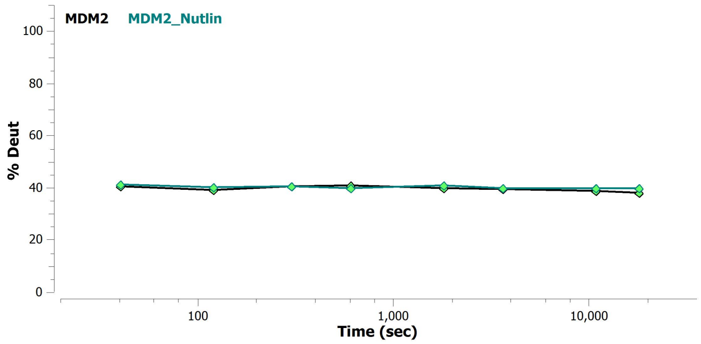

122-142: VSENRCHLEGGSDQKDLVQEL (#39)

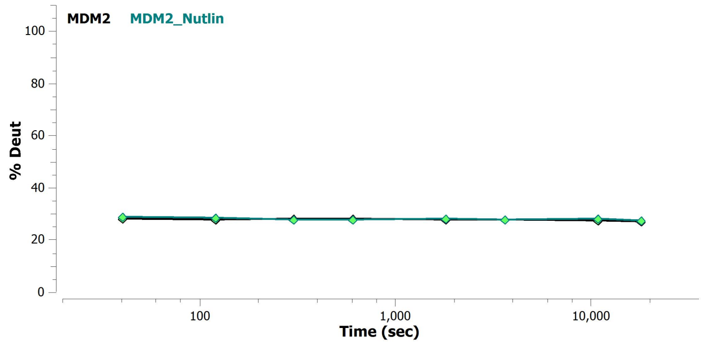

125-141: NRCHLEGGSDQKDLVQE (#40)

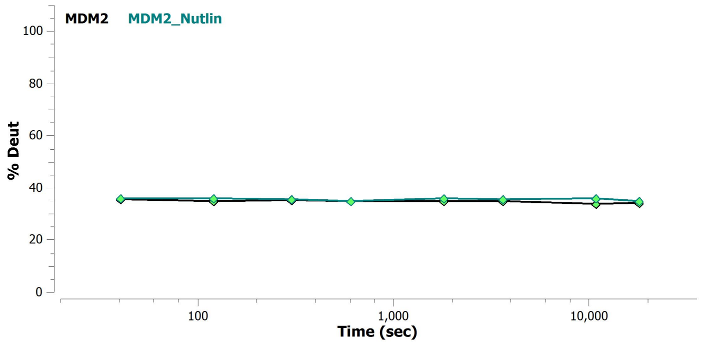

130-142: EGGSDQKDLVQEL (#41)

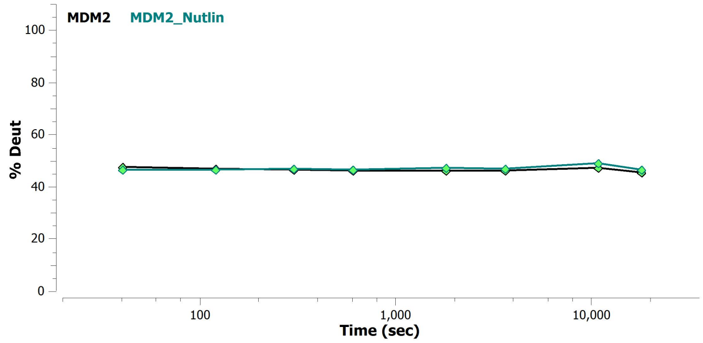

138-152: LVQELQEEKPSSSHL (#42)

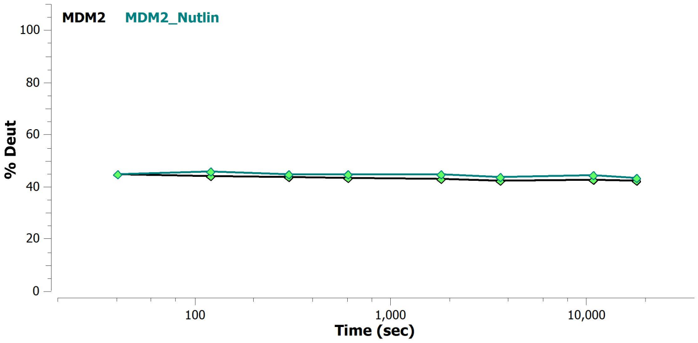

142-152: LQEEKPSSSHL (#43)

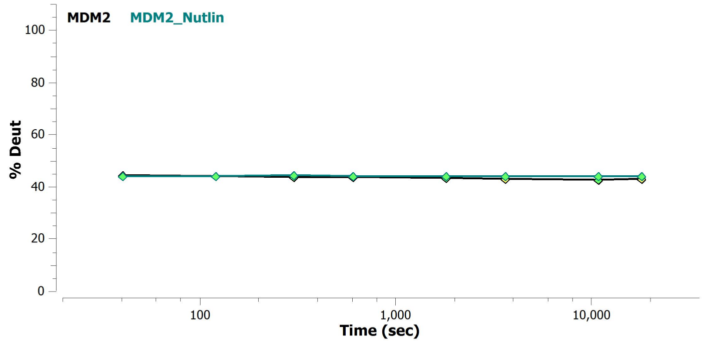

142-153: LQEEKPSSSHLV (#44)

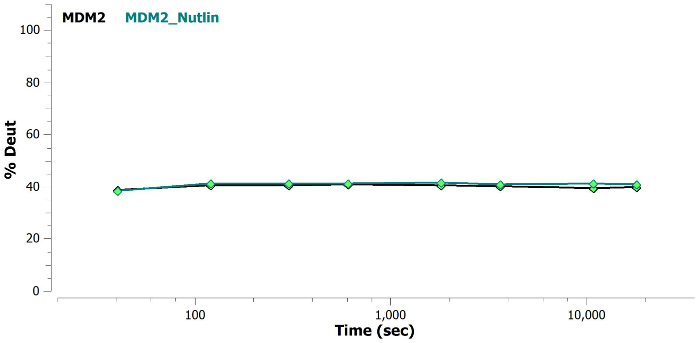

143-152: QEEKPSSSHL (#45)

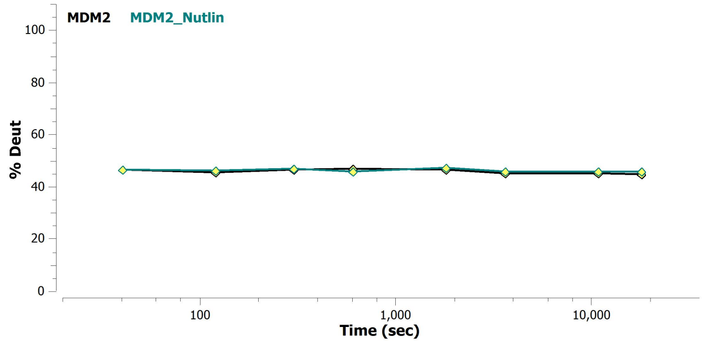

153-167: VSRPSTSSRRRAISE (#46)

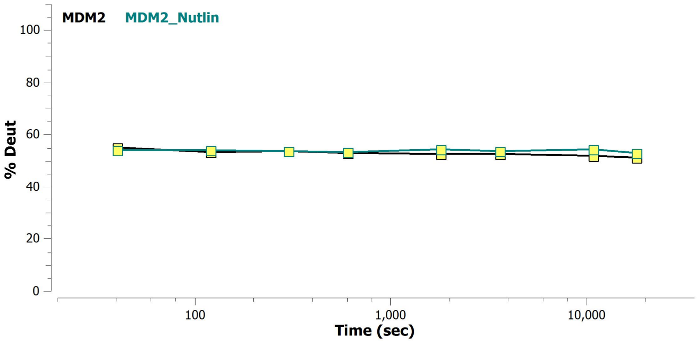

168-190: TEENSDELSGERQRKRHKSDSIS (#47)

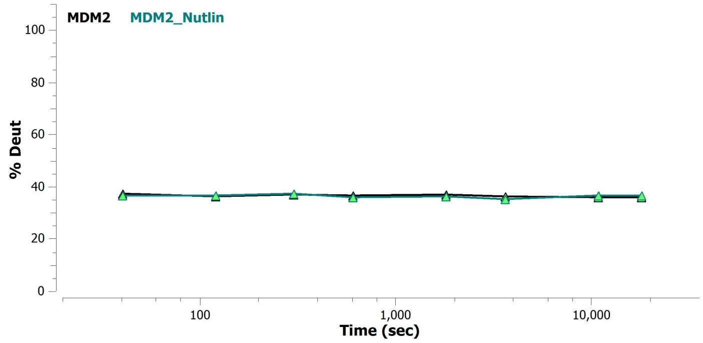

207-224: CERSSSSESTGTPSNPDL (#48)

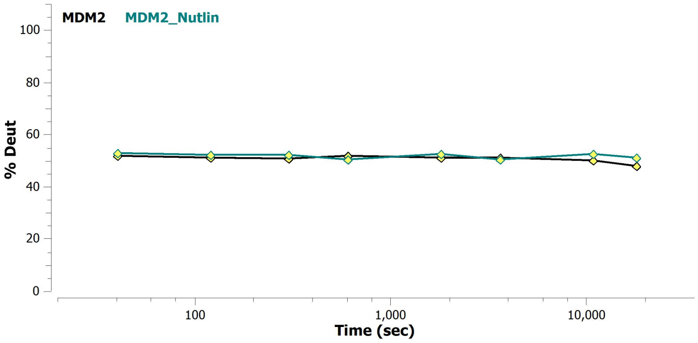

207-226: CERSSSSESTGTPSNPDLDA (#49)

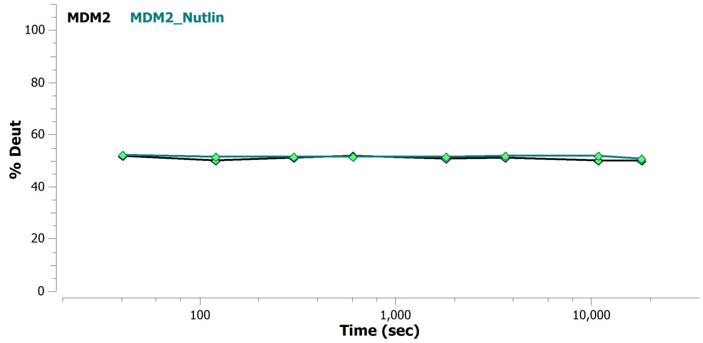

208-224: ERSSSSESTGTPSNPDL (#50)

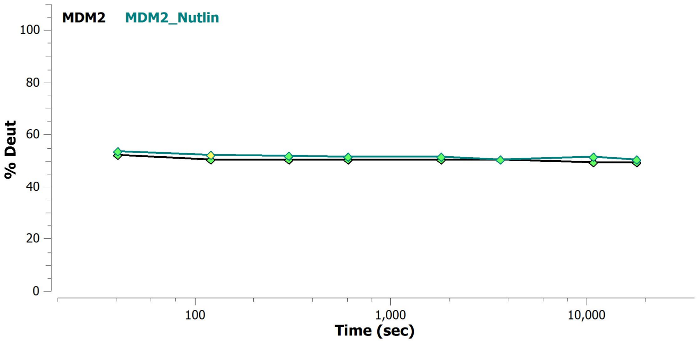

209-226: RSSSSESTGTPSNPDLDA (#51)

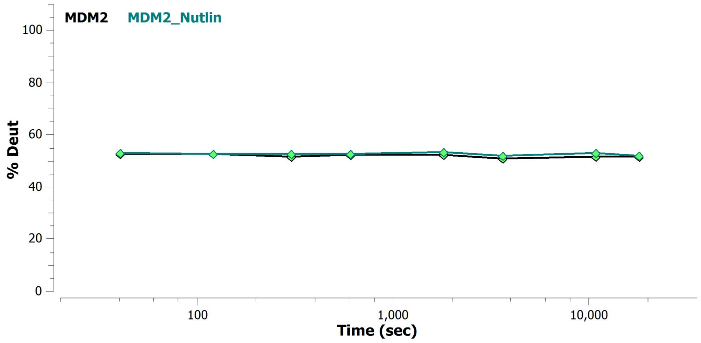

231-244: HSGDWLDQDSVSDQ (#52)

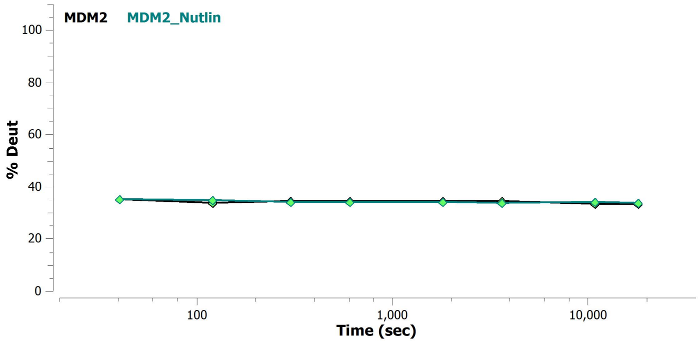

235-245: WLDQDSVSDQF (#53)

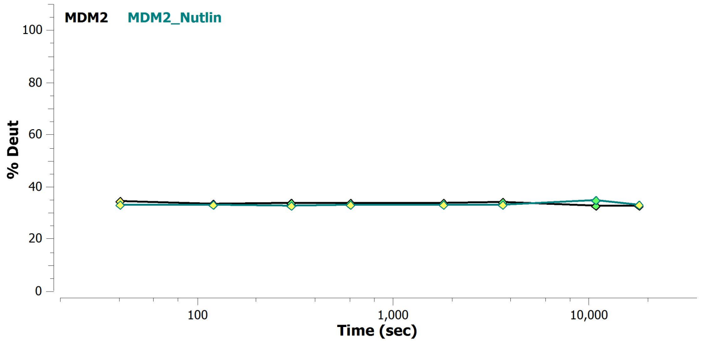

259-271: YSLSEEGQELSDE (#54)

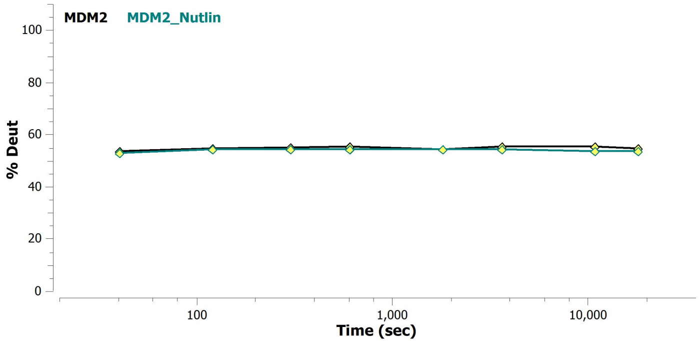

291-299: FEEDPEISL (#55)

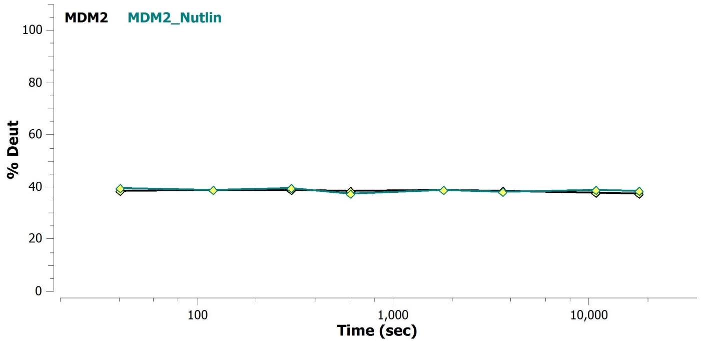

303-319: WKCTSCNEMNPPLPSHC (#56)

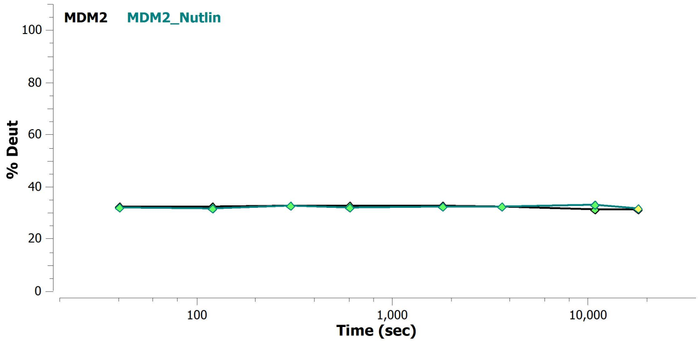

309-319: NEMNPPLPSHC (#57)

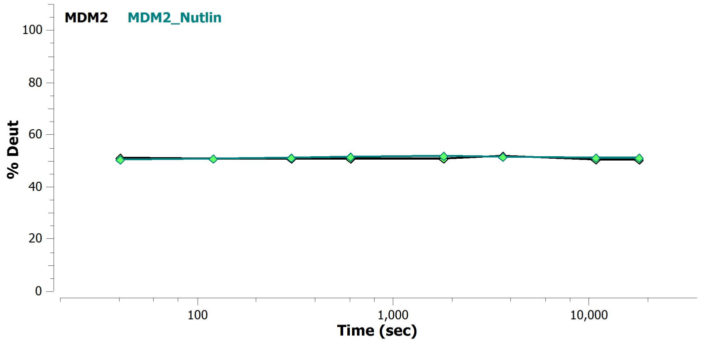

323-343: WALRENWLPEDKGKDKGEISE (#58)

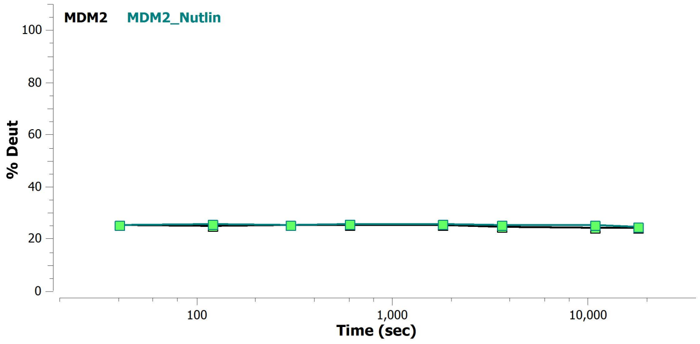

325-343: LRENWLPEDKGKDKGEISE (#59)

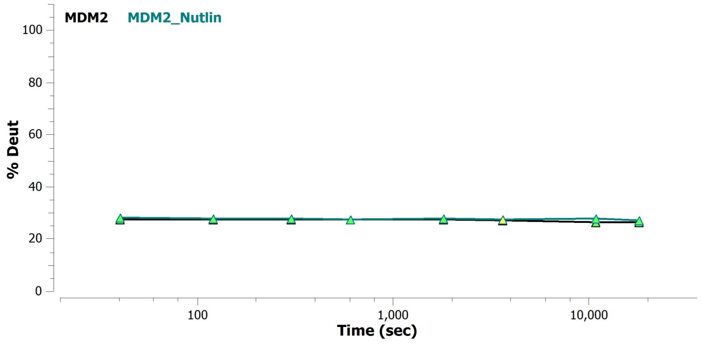

328-343: NWLPEDKGKDKGEISE (#60)

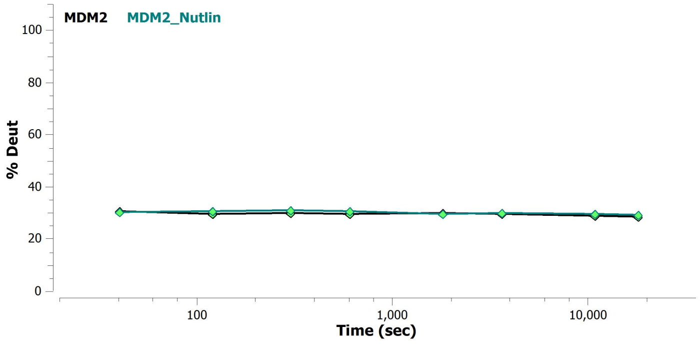

344-356: KAKLENSTQAEEG (#61)

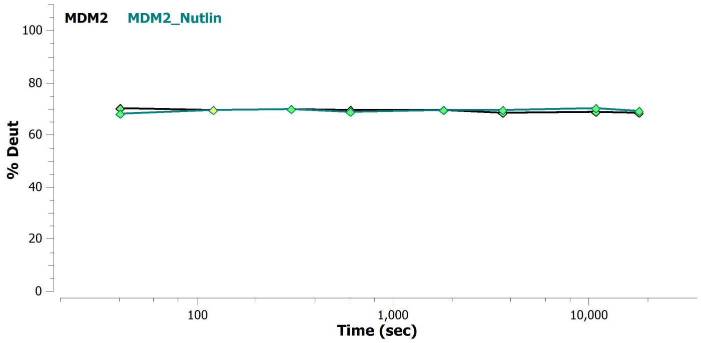

353-374: AEEGFDVPDCKKTIVNDSRESC (#62)

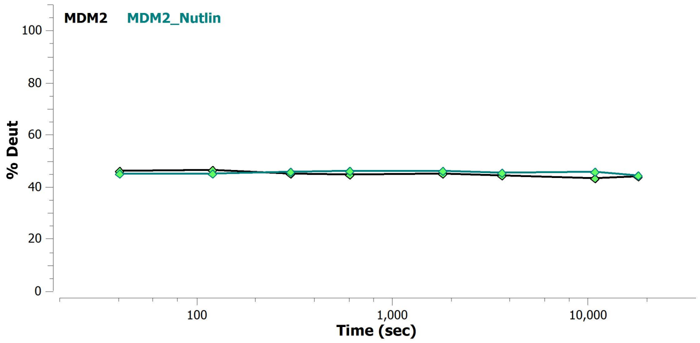

357-374: FDVPDCKKTIVNDSRESC (#63)

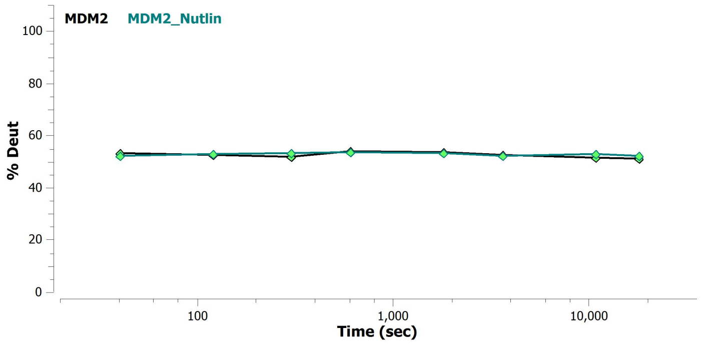

375-390: VEENDDKITQASQSQE (#64)

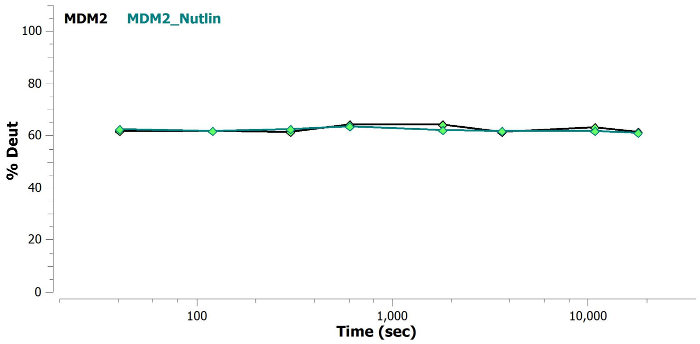

394-409: YSQPSTSSSIIYSSQE (#65)

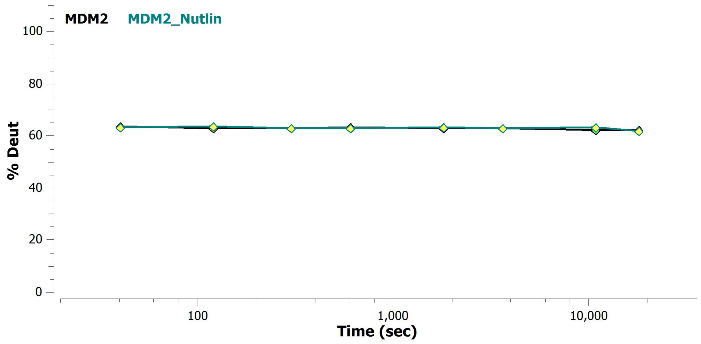

403-413: IIYSSQEDVKE (#66)

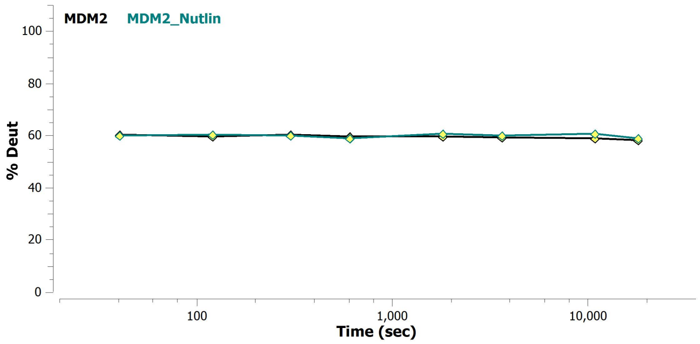

410-427: DVKEFEREETQDKEESVE (#67)

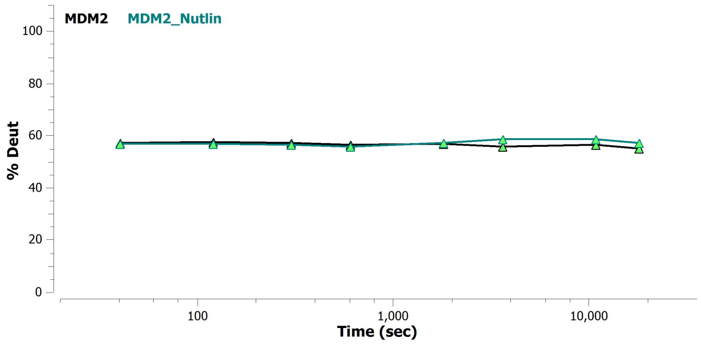

414-427: FEREETQDKEESVE (#68)

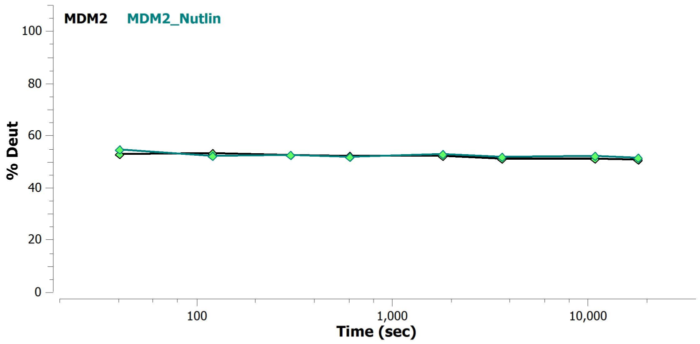

414-436: FEREETQDKEESVESSLPLNAIE (#69)

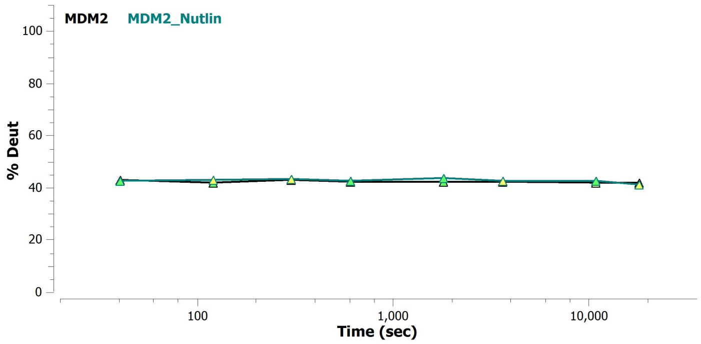

437-461: PCVICQGRPKNGCIVHGKTGHLMAC (#70)

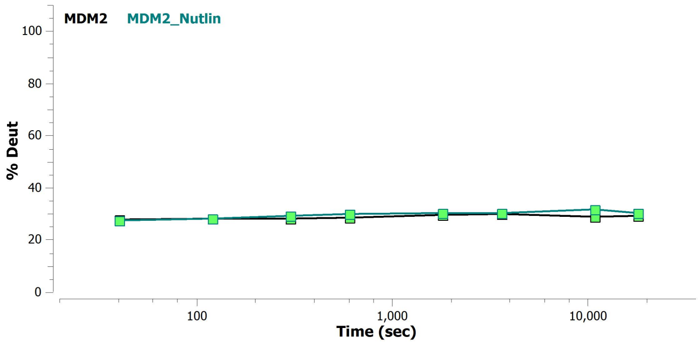

Supplement: Supplemental Data [file supp_RA118.000573_134890_0_supp_50211_p258m0.pdf]
