# Supplementary figures and images for: The Sequence-specific Peptide-binding Activity of the Protein Sulfide Isomerase AGR2 Directs Its Stable Binding to the Oncogenic Receptor EpCAM
Source: Mol Cell Proteomics. 2018 Jan 16;17(4):737–63. doi: 10.1074/mcp.RA118.000573 (PMC5880107; doi:10.1074/mcp.RA118.000573)

## Slide 1
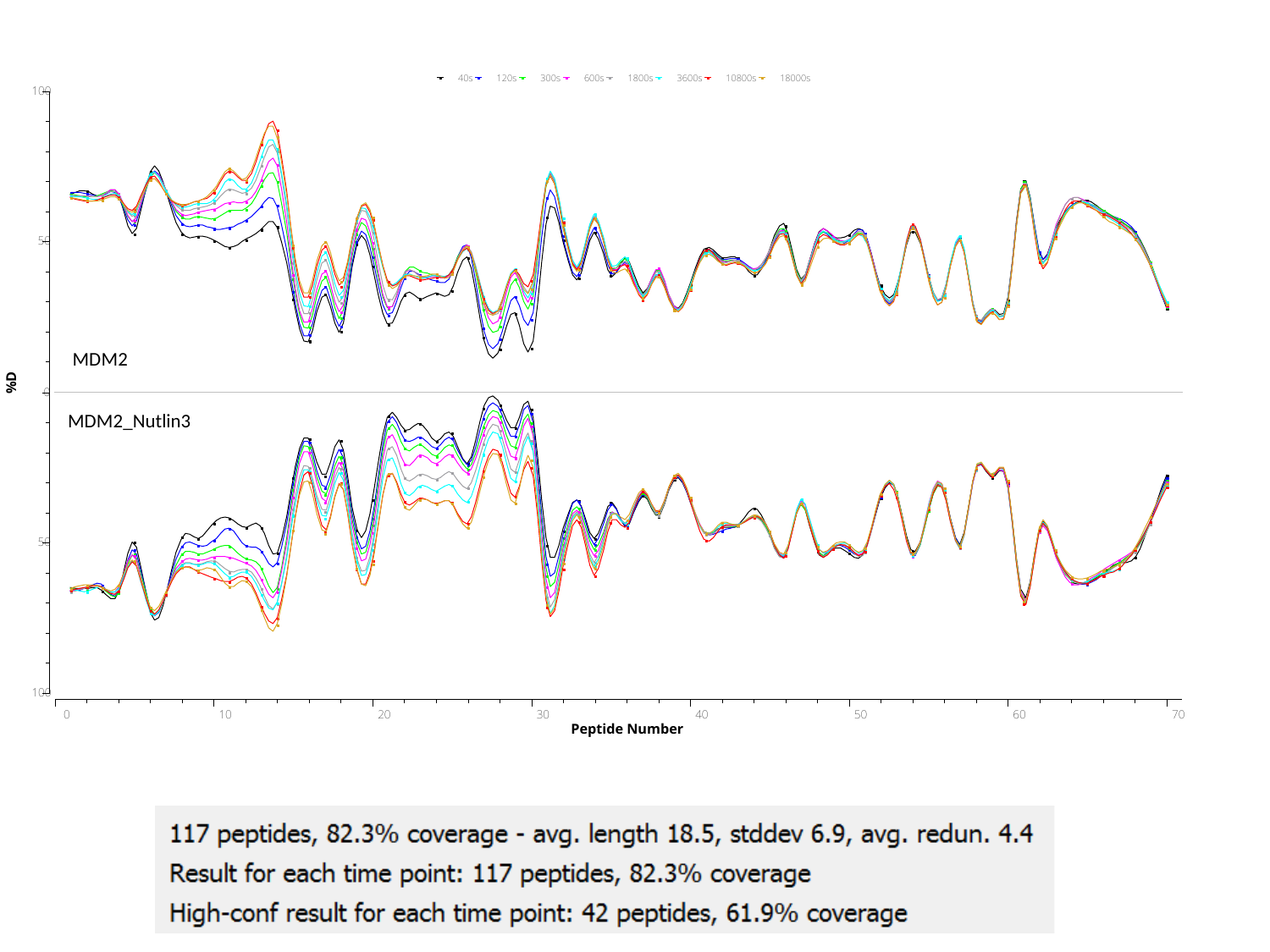

MDM2
MDM2_Nutlin3

Supplement: Supplemental Data [file supp_RA118.000573_134890_0_supp_50210_p25890.pptx]

## Slide 1
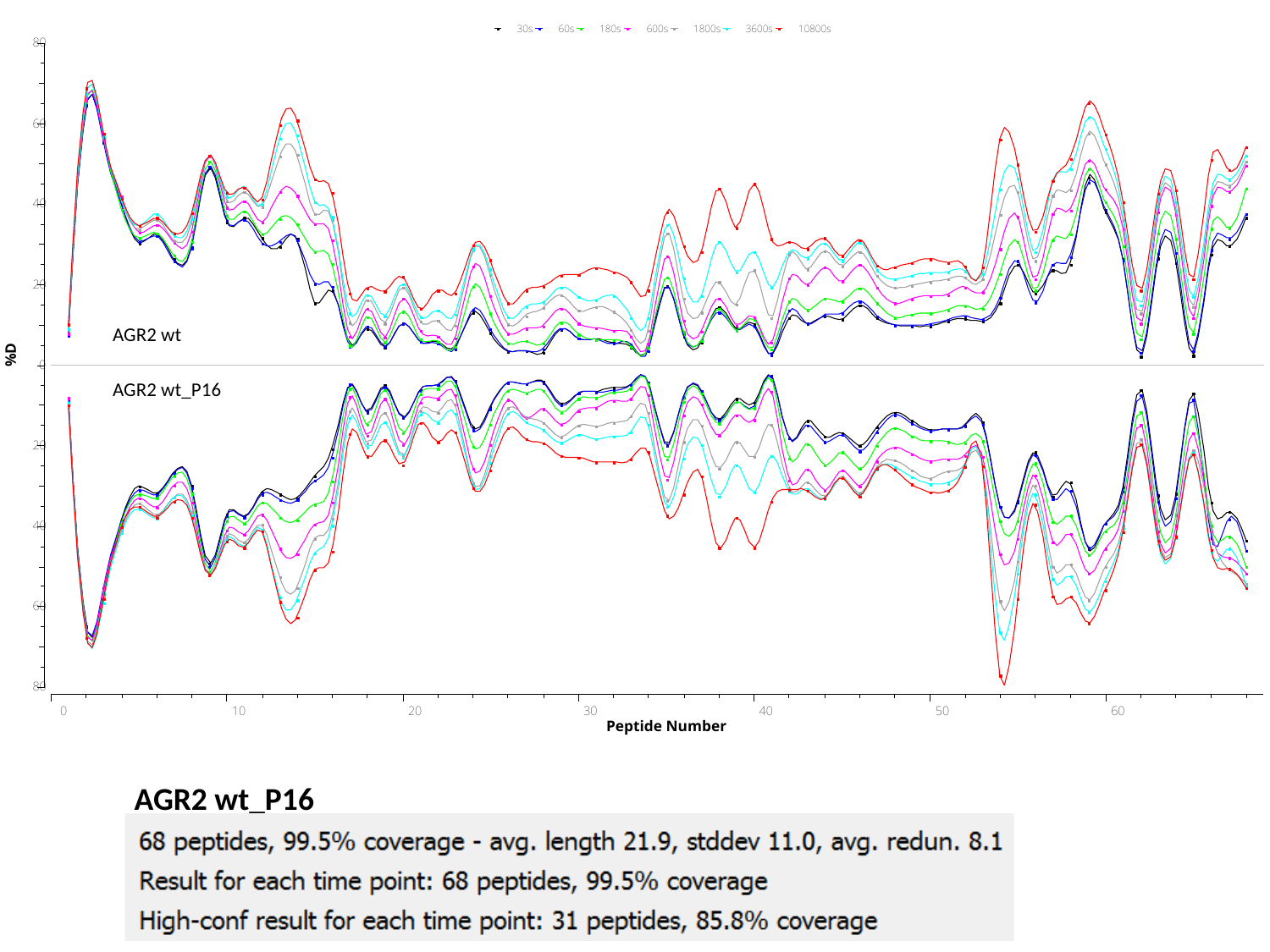

AGR2 wt
AGR2 wt_P16
AGR2 wt_P16

Supplement: Supplemental Data [file supp_RA118.000573_134890_0_supp_50208_p25850.pptx]

## Slide 1
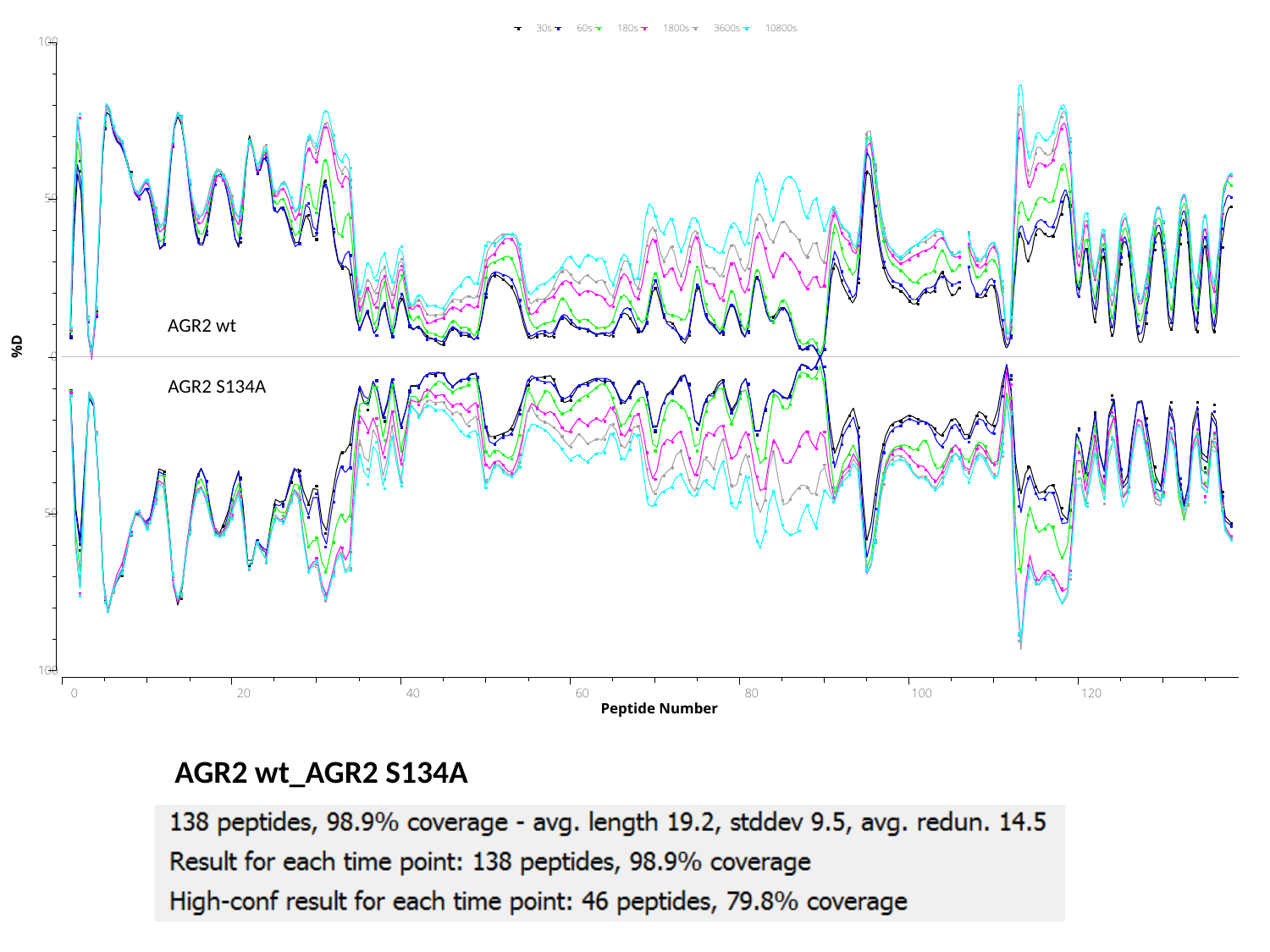

AGR2 wt
AGR2 S134A
AGR2 wt_AGR2 S134A

Supplement: Supplemental Data [file supp_RA118.000573_134890_0_supp_50206_p258tz.pptx]

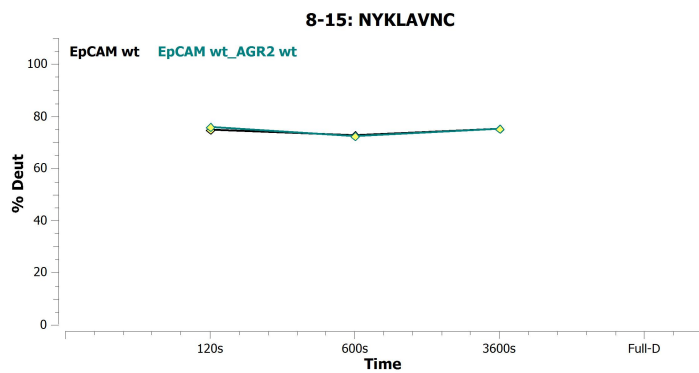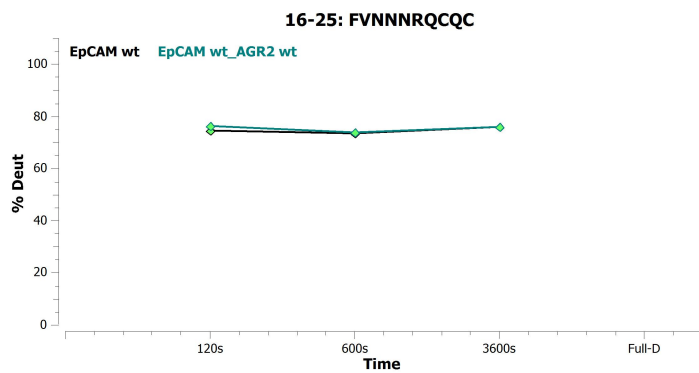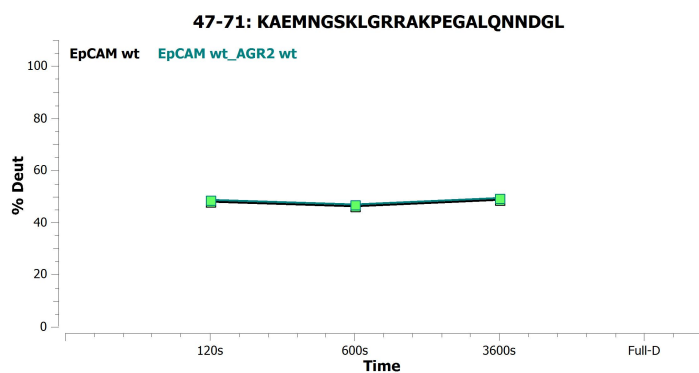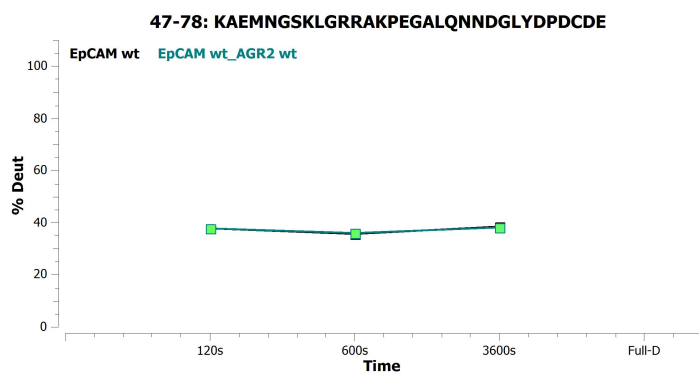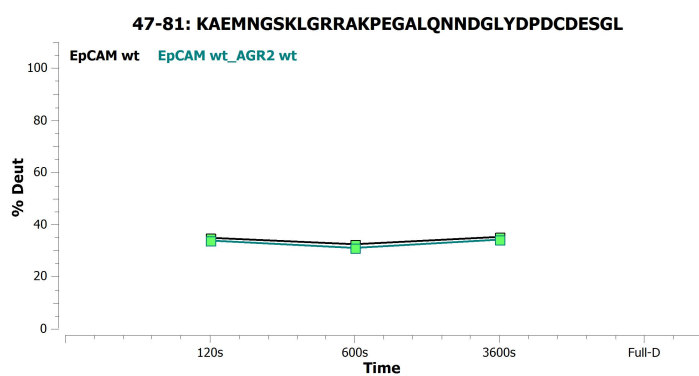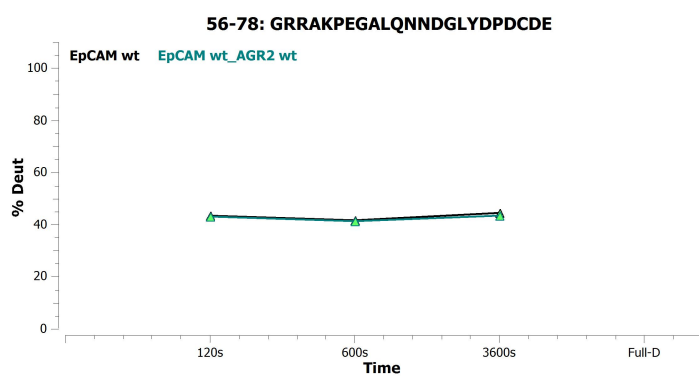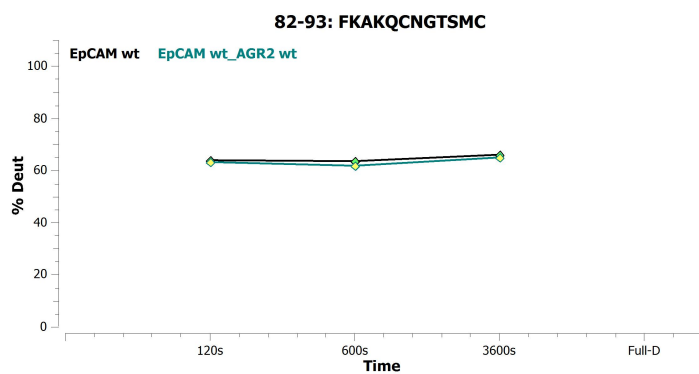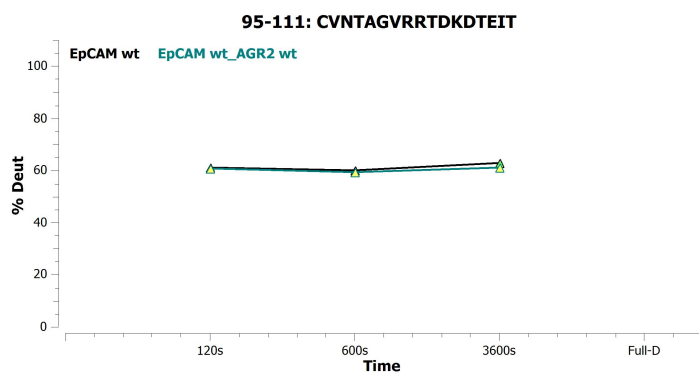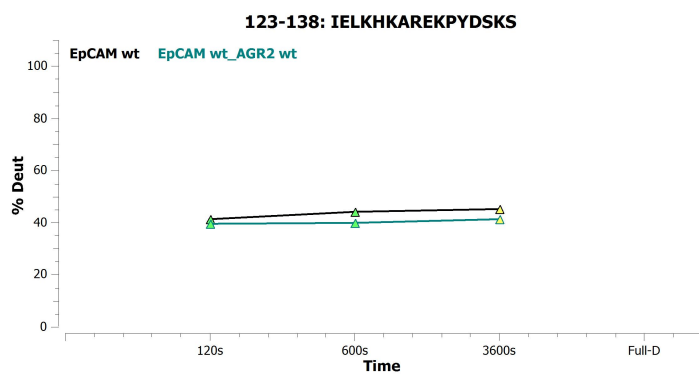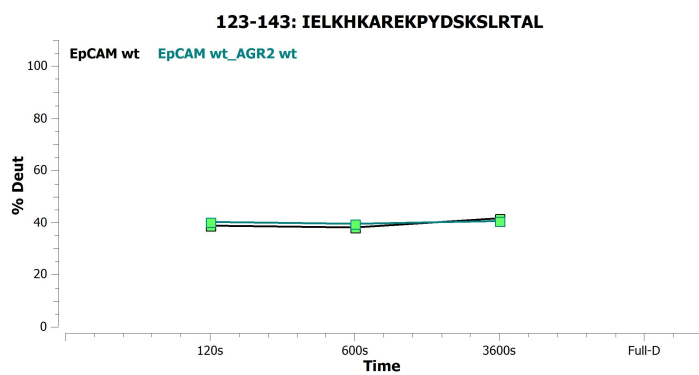

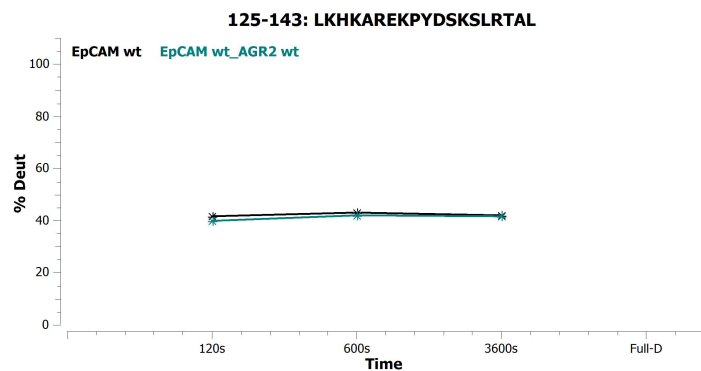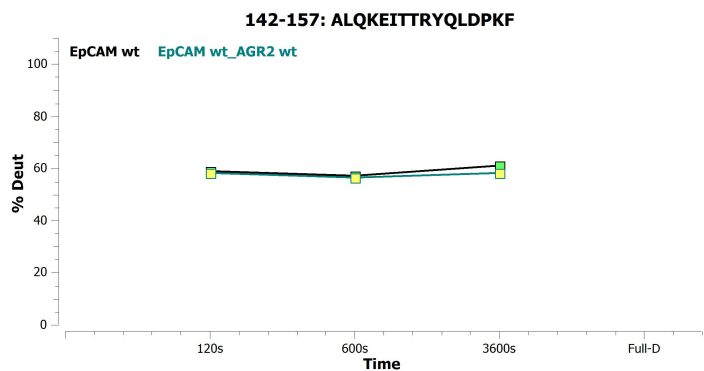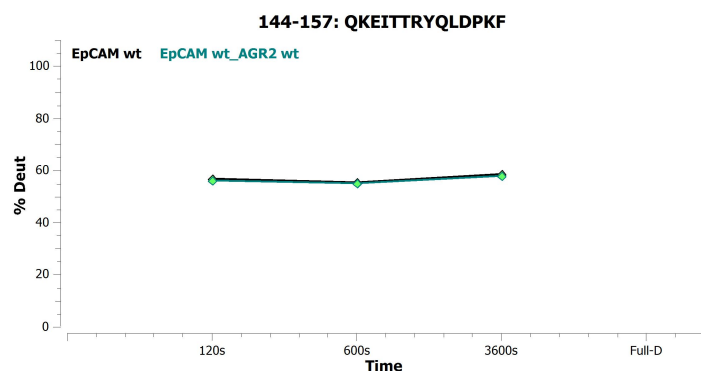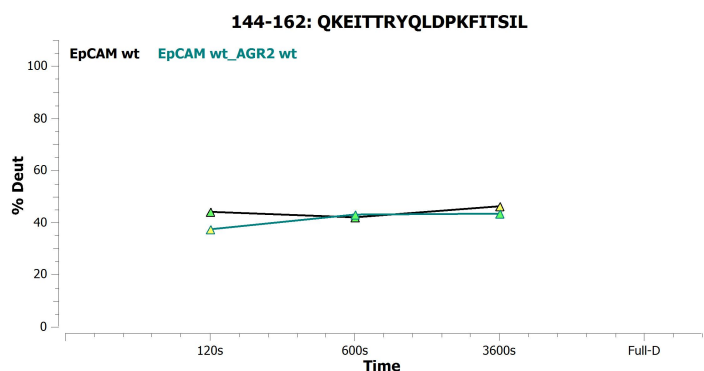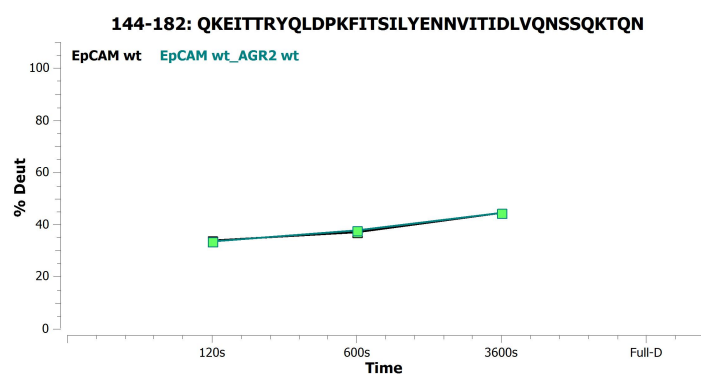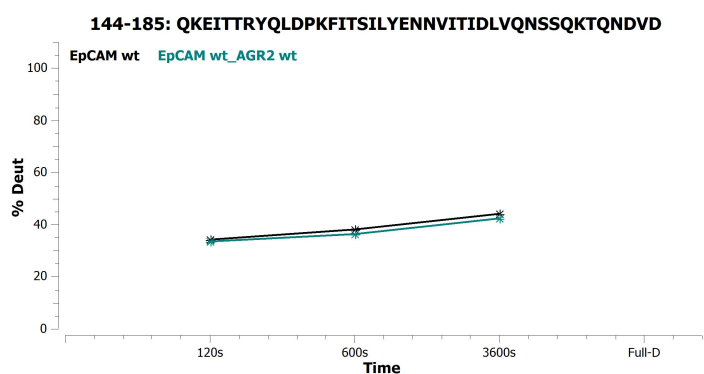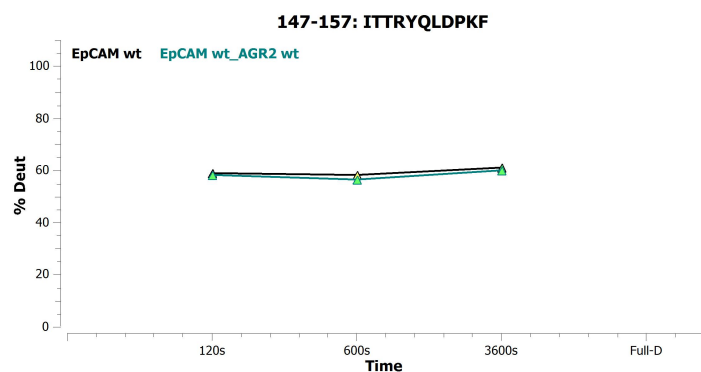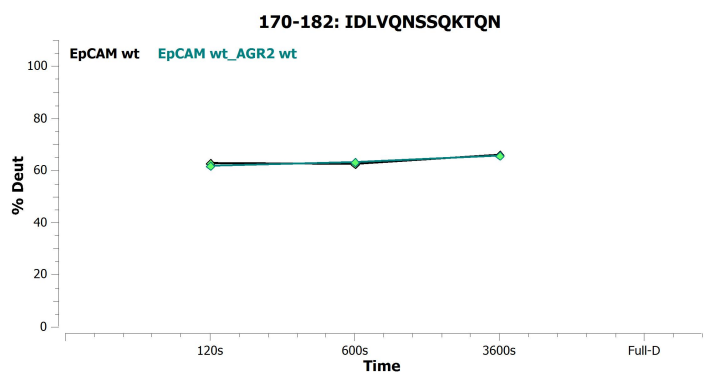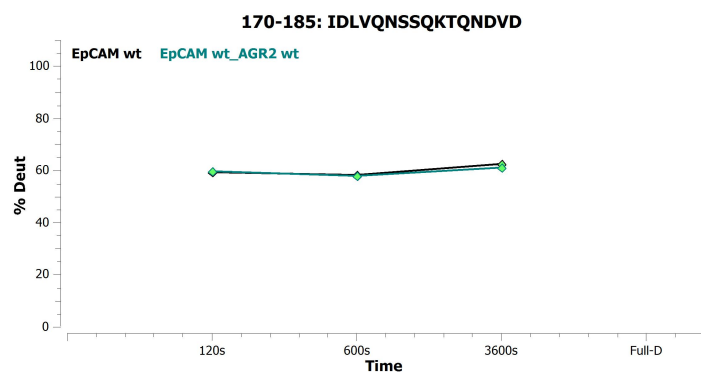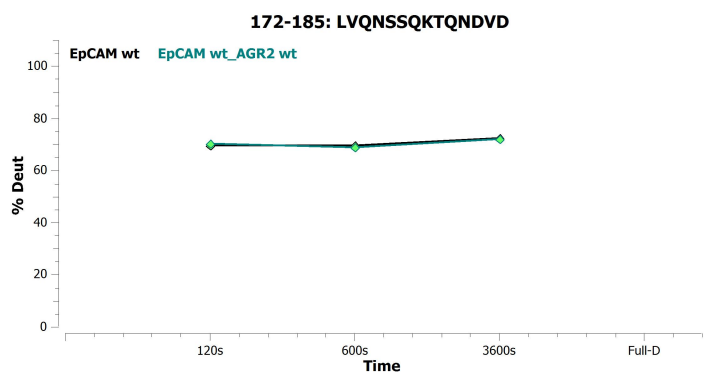

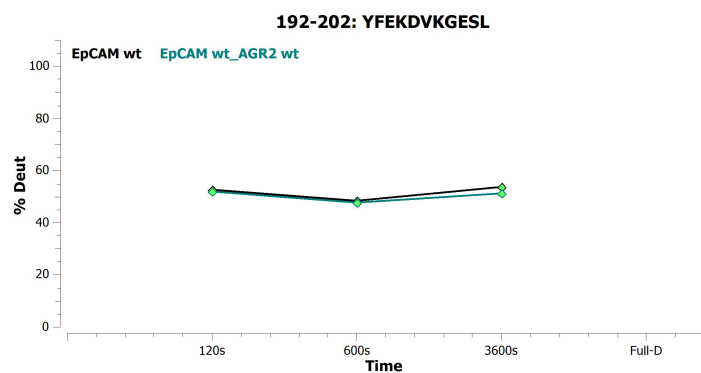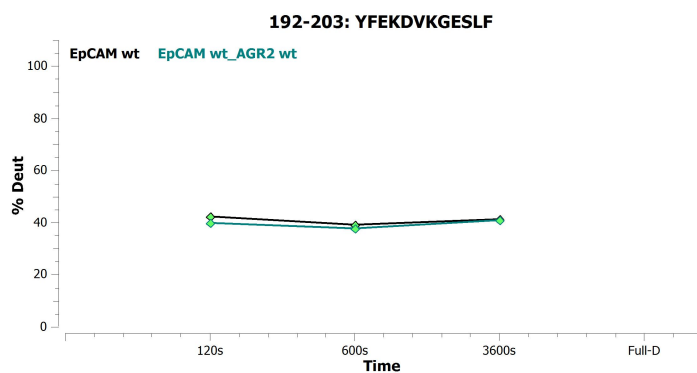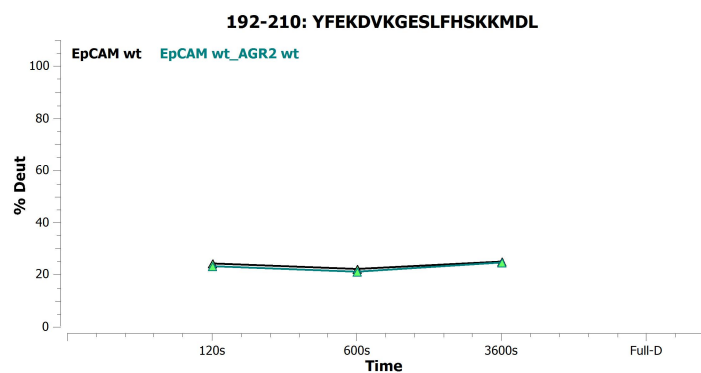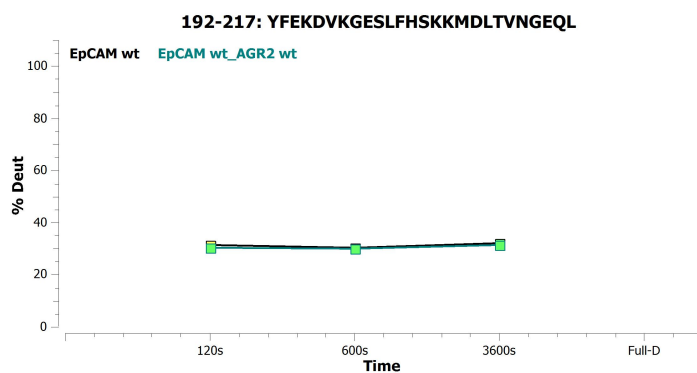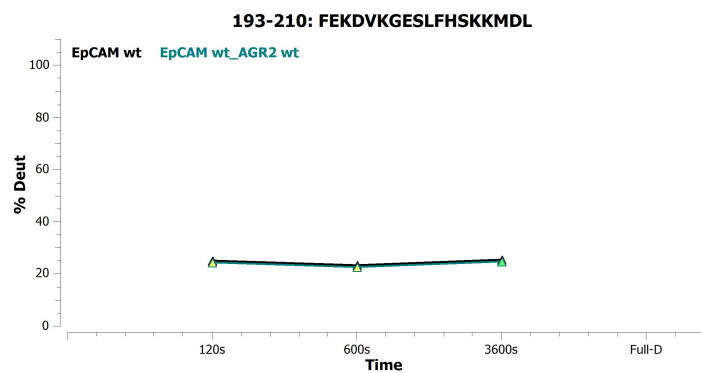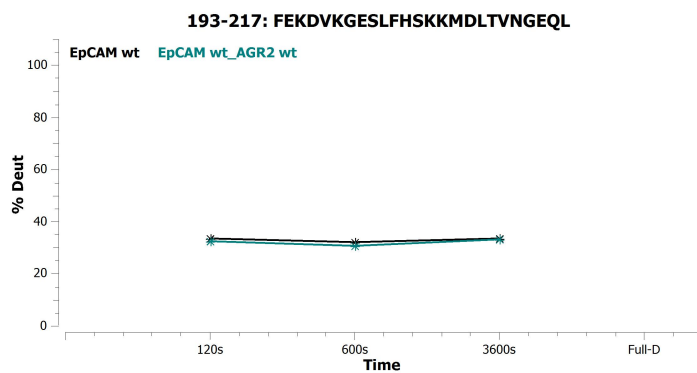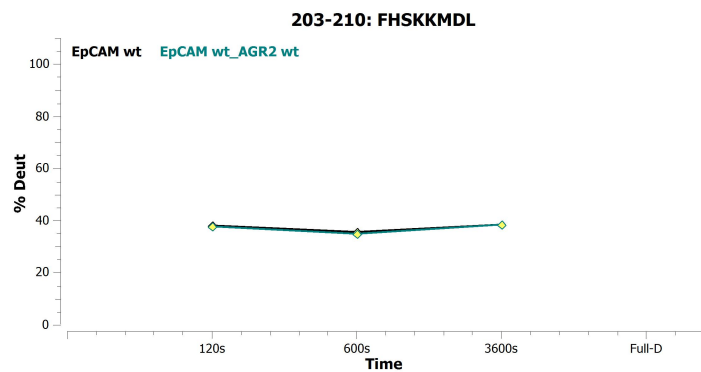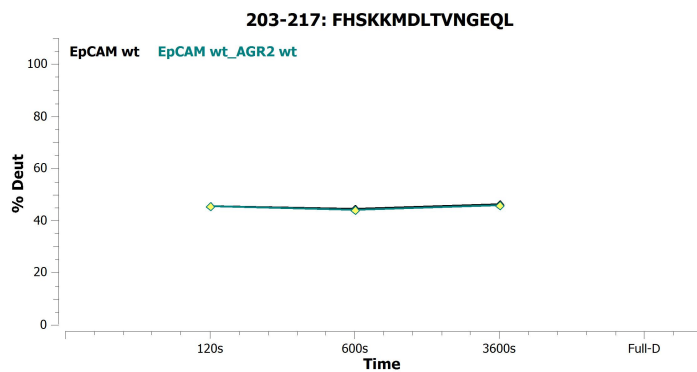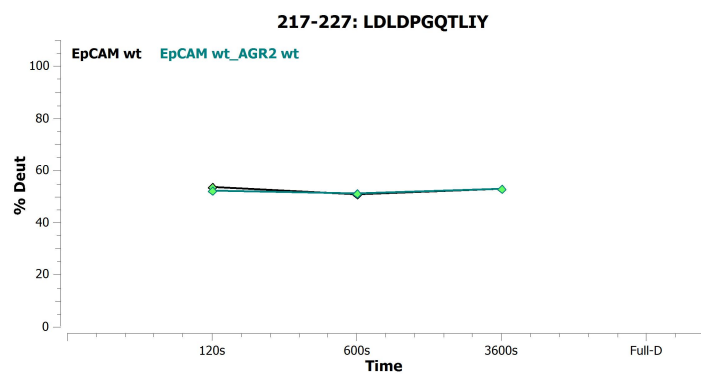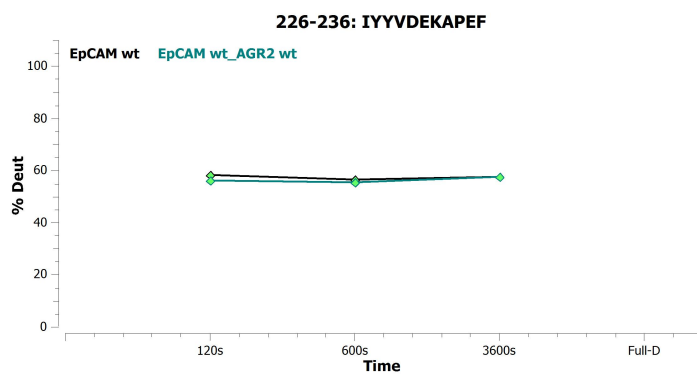

227-236: YYVDEKAPEF

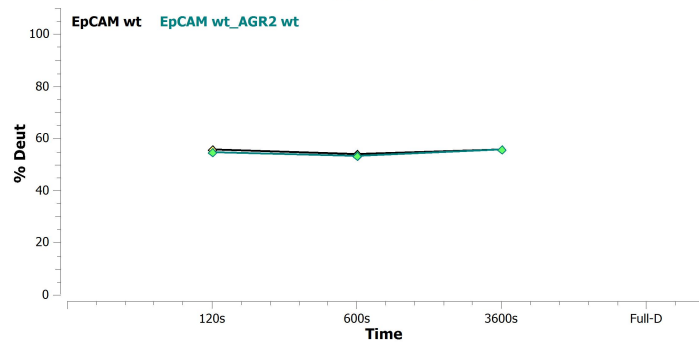

228-236: YVDEKAPEF

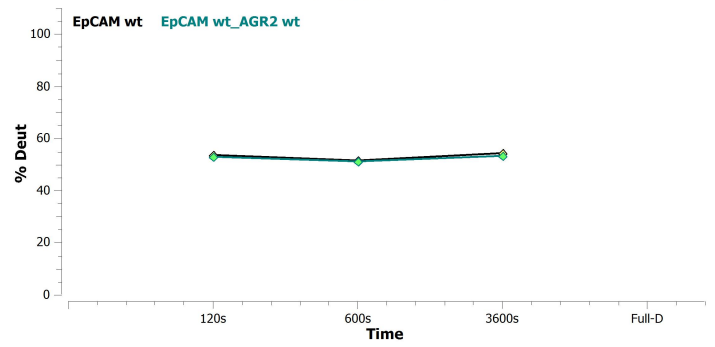

Supplement: Supplemental Data [file supp_RA118.000573_134890_0_supp_50205_p258tz.pdf]

## Slide 1
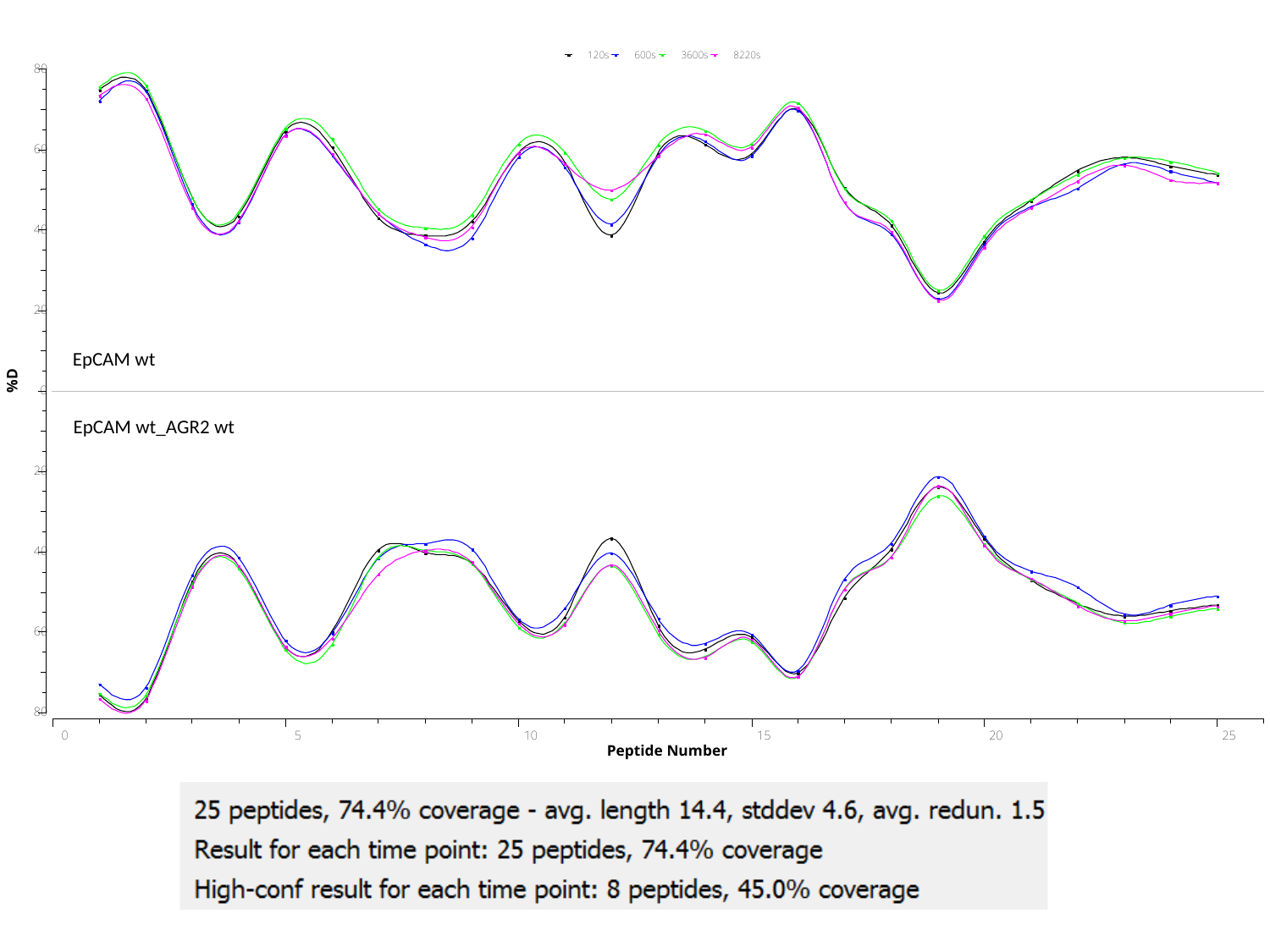

EpCAM wt
EpCAM wt_AGR2 wt

Supplement: Supplemental Data [file supp_RA118.000573_134890_0_supp_50204_p258tz.pptx]

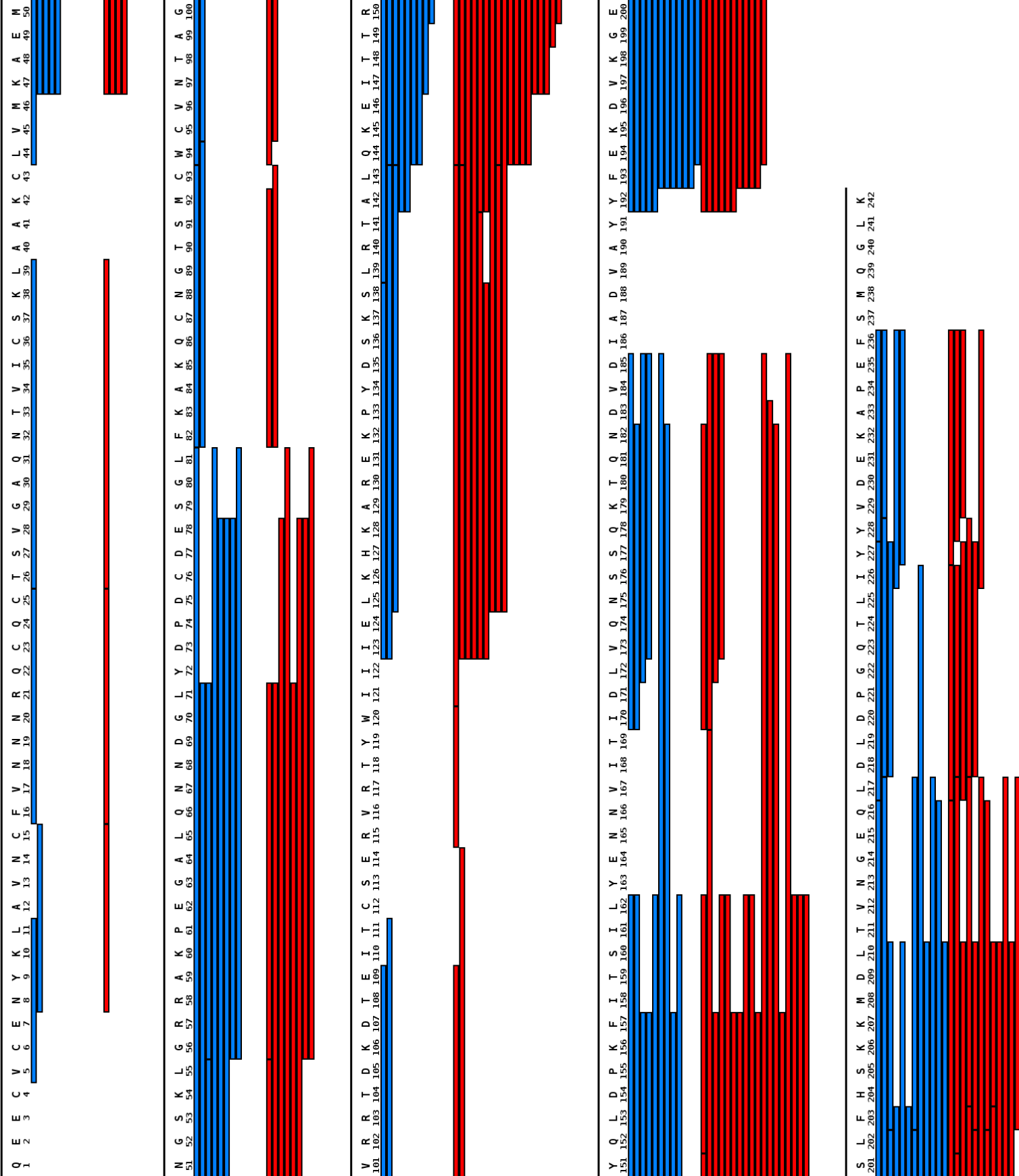

Supplement: Supplemental Data [file supp_RA118.000573_134890_0_supp_50202_p258tz.pdf]
