# Supplementary material for: The Sequence-specific Peptide-binding Activity of the Protein Sulfide Isomerase AGR2 Directs Its Stable Binding to the Oncogenic Receptor EpCAM
Source: Mol Cell Proteomics. 2018 Jan 16;17(4):737–63. doi: 10.1074/mcp.RA118.000573 (PMC5880107; doi:10.1074/mcp.RA118.000573)

2-16: SYYHHHHHHLESTSL

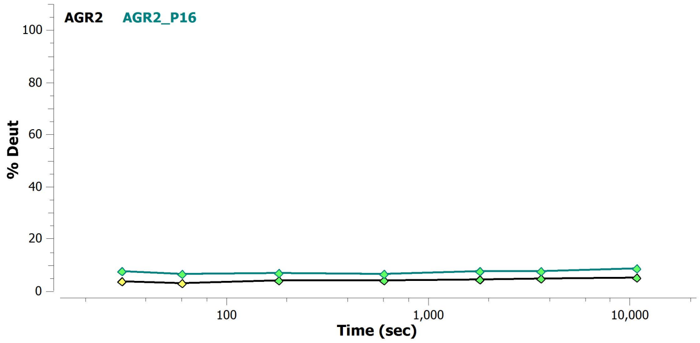

17-27: YKKAGFEGDRT

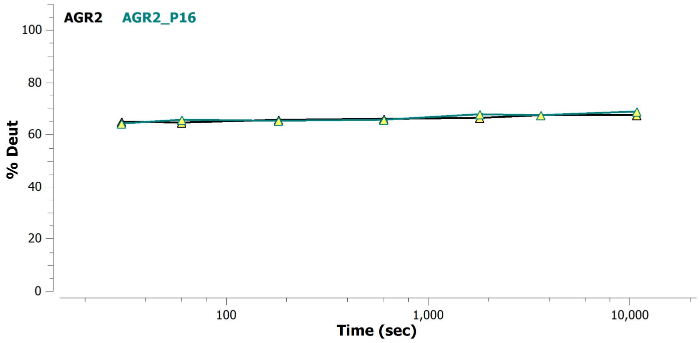

17-30: YKKAGFEGDRTMRD

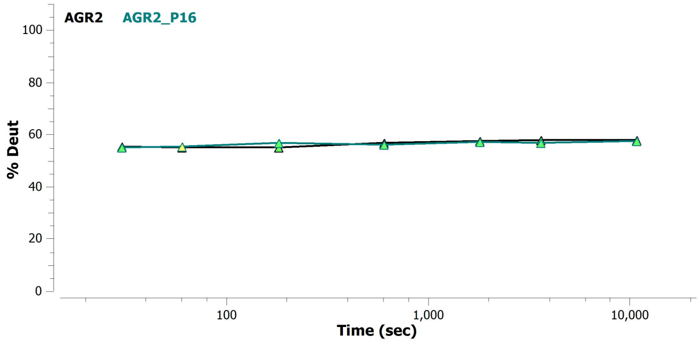

17-52: YKKAGFEGDRTMRDTTVKPGAKKDTKDSRPKLPQTL

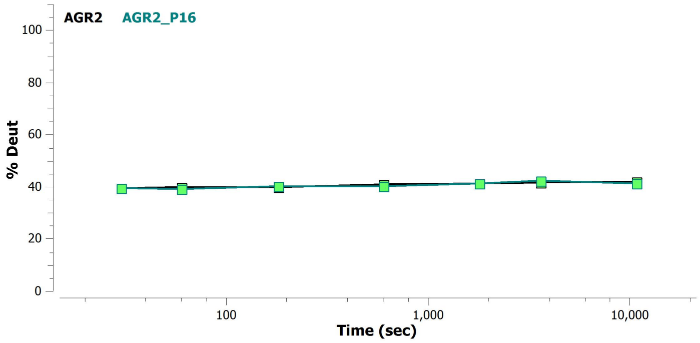

17-56: YKKAGFEGDRTMRDTTVKPGAKKDTKDSRPKLPQTLSRGW

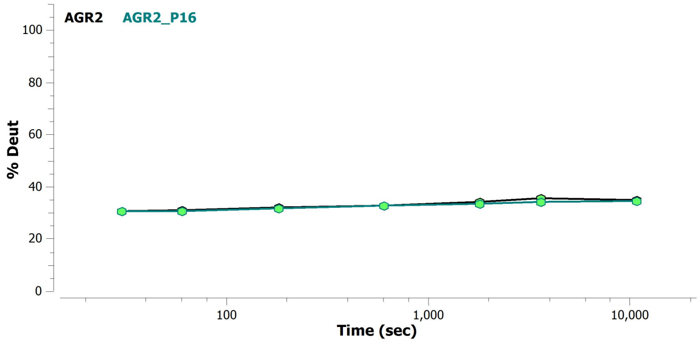

17-59: YKKAGFEGDRTMRDTTVKPGAKKDTKDSRPKLPQTLSRGWGDQ

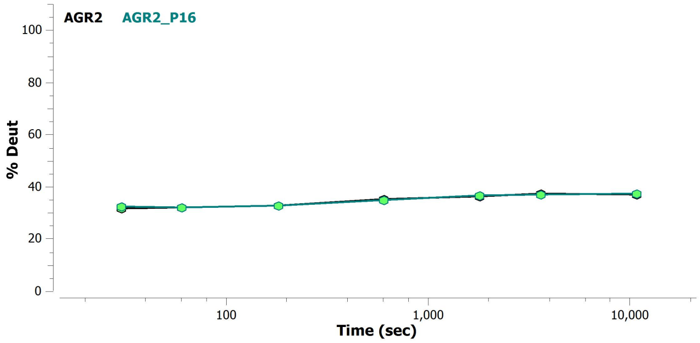

17-60: YKKAGFEGDRTMRDTTVKPGAKKDTKDSRPKLPQTLSRGWGDQL

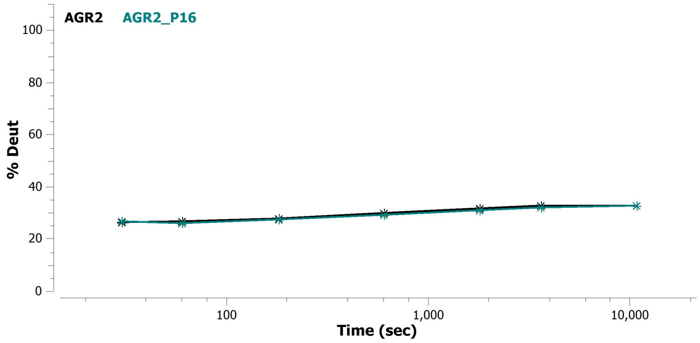

25-60: DRTMRDTTVKPGAKKDTKDSRPKLPQTLSRGWGDQL

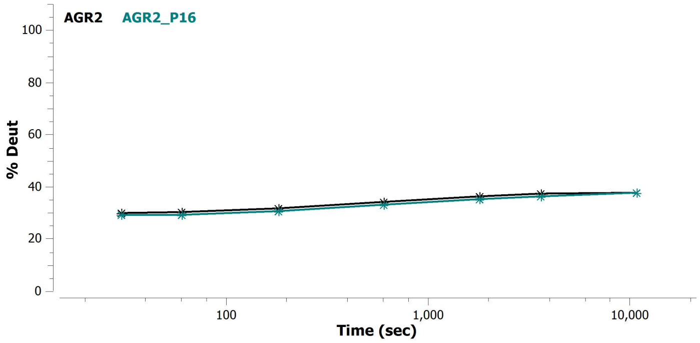

31-52: TTVKPGAKKDTKDSRPKLPQTL

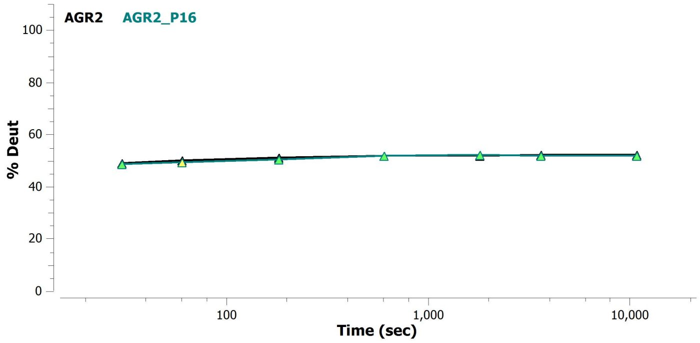

31-56: TTVKPGAKKDTKDSRPKLPQTLSRGW

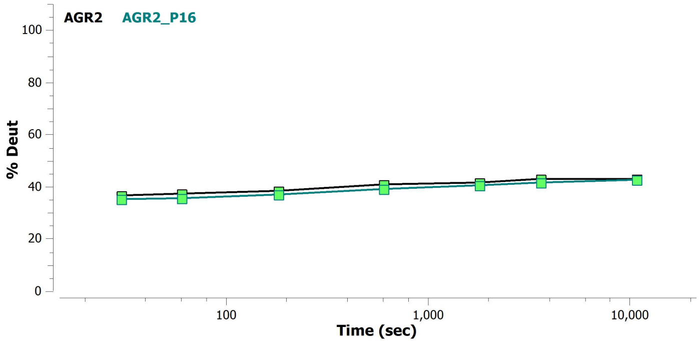

31-59: TTVKPGAKKDTKDSRPKLPQTLSRGWGDQ

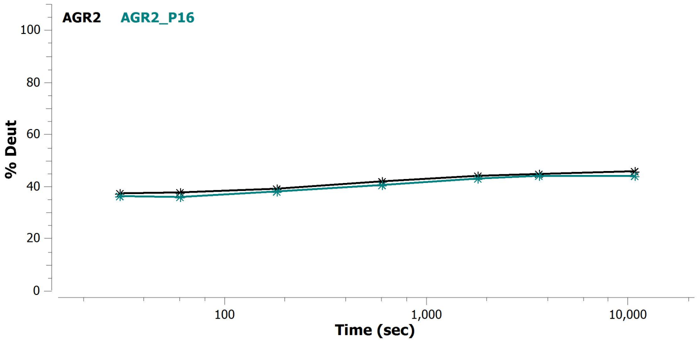

31-60: TTVKPGAKKDTKDSRPKLPQTLSRGWGDQL

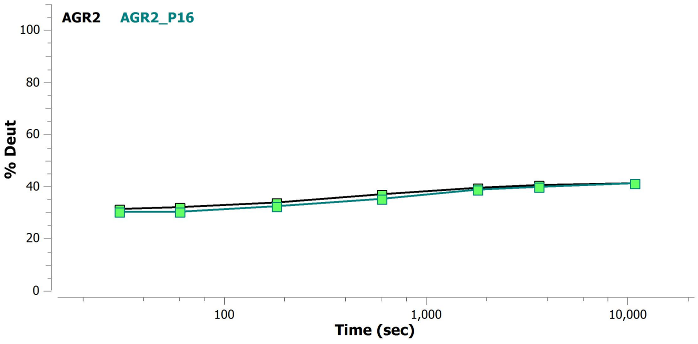

52-60: LSRGWGDQL

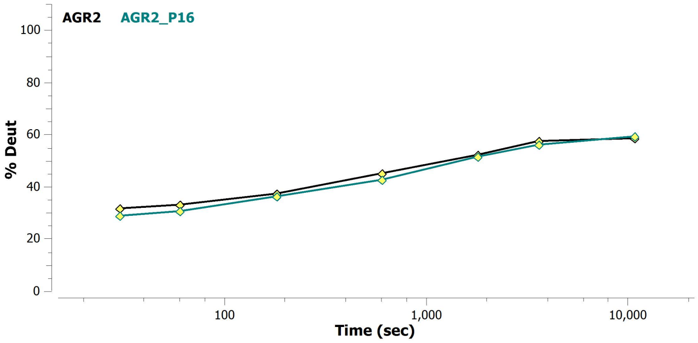

53-60: SRGWGDQL

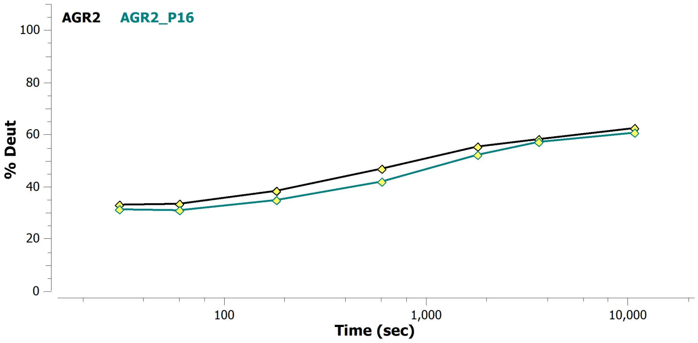

61-67: IWTQTYE

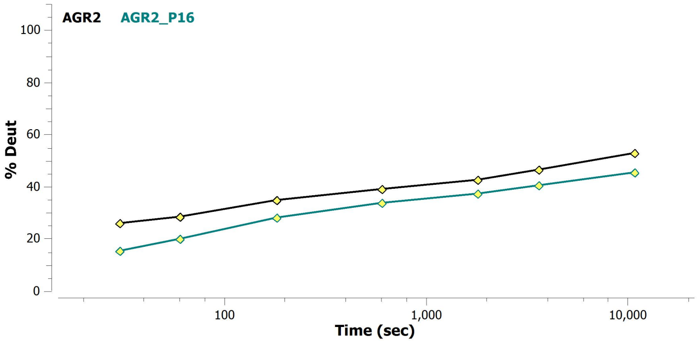

61-70: IWTQTYEEAL

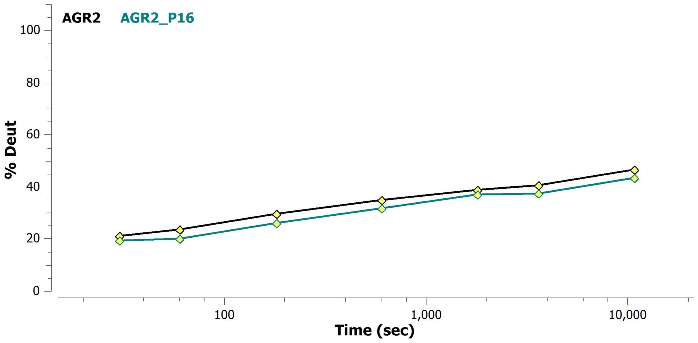

69-111: ALYKSKTSSKPLMIIHHLDECPHSQALKKVFAENKEIQKLAEQ

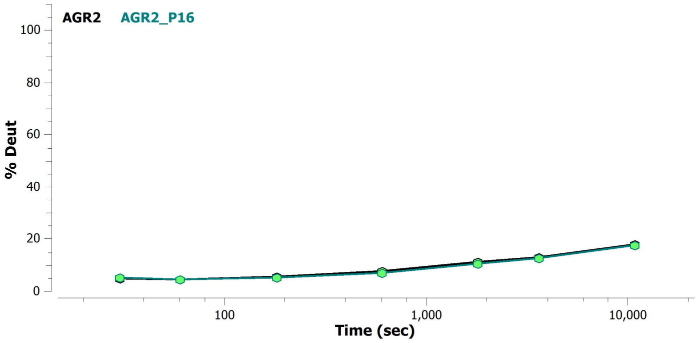

70-81: LYKSKTSSKPLM

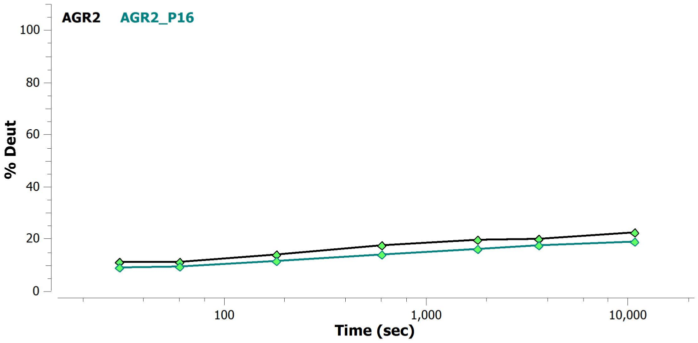

70-111: LYKSKTSSKPLMIIHHLDECPHSQALKKVFAENKEIQKLAEQ

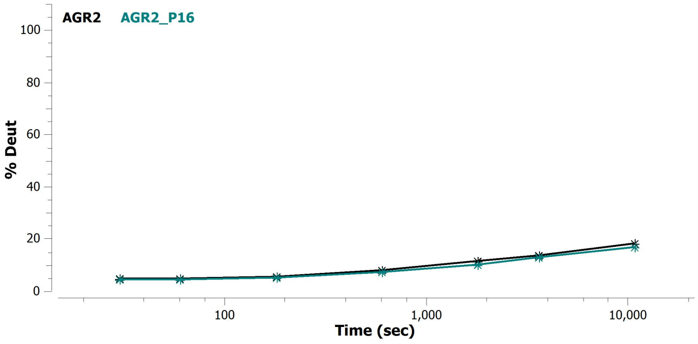

71-81: YKSKTSSKPLM

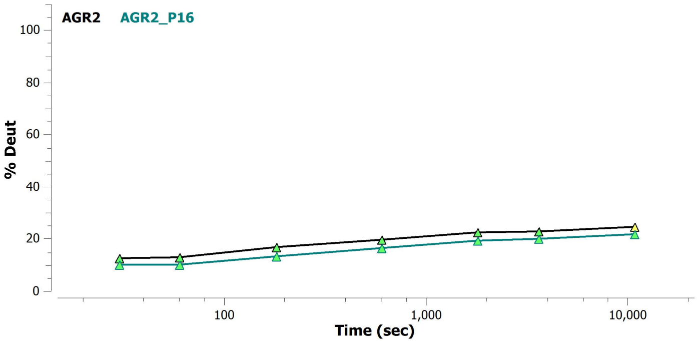

71-104: YKSKTSSKPLMIIHHLDECPHSQALKKVFAENKE

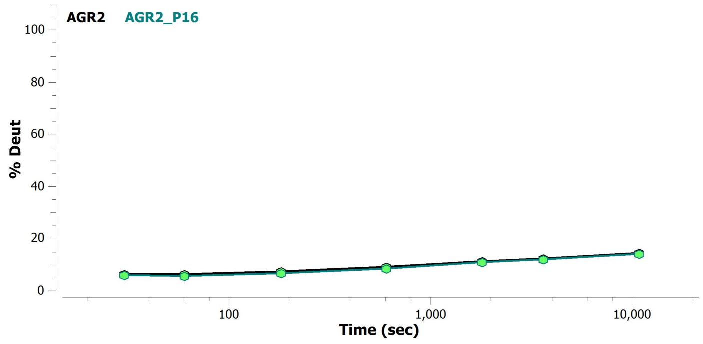

71-111: YKSKTSSKPLMIIHHLDECPHSQALKKVFAENKEIQKLAEQ

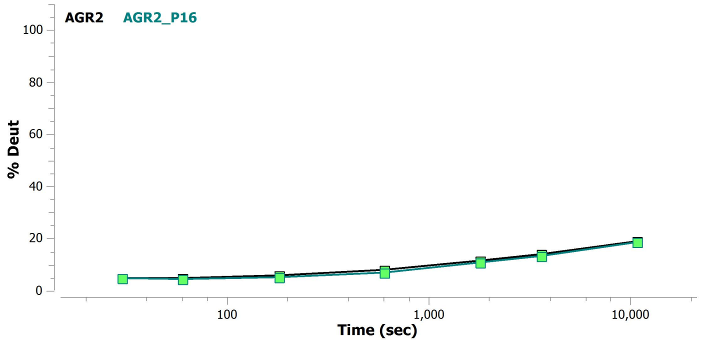

71-112: YKSKTSSKPLMIIHHLDECPHSQALKKVFAENKEIQKLAEQF

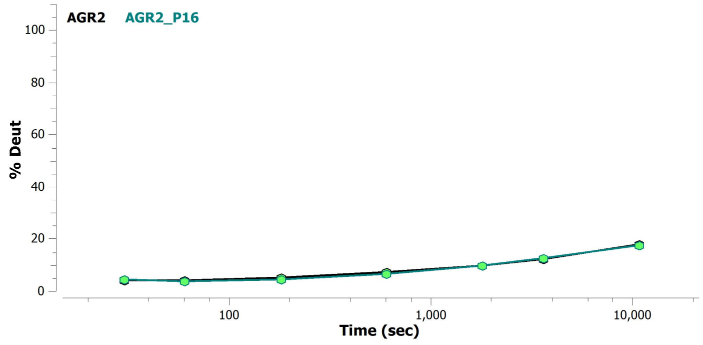

82-94: IIHHLDECPHSQA

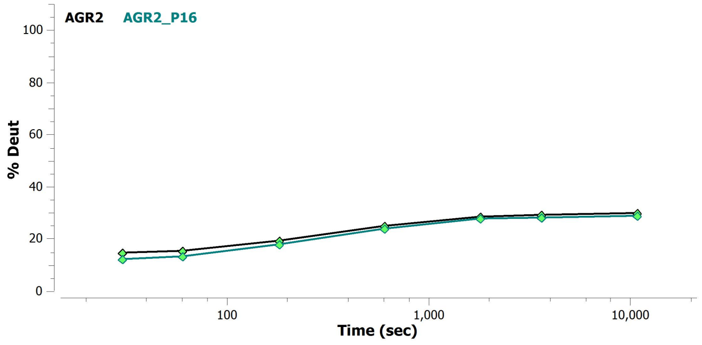

82-95: IIHHLDECPHSQAL

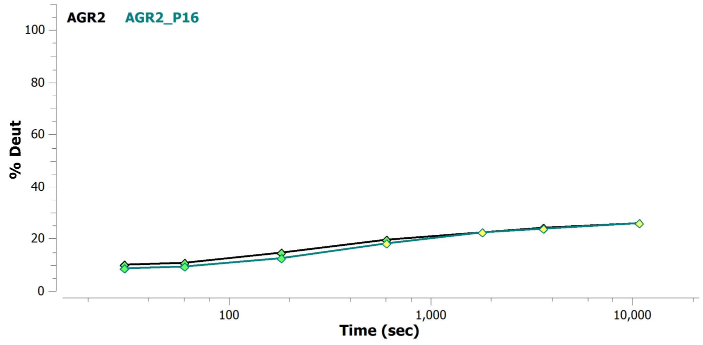

82-99: IIHHLDECPHSQALKKVF

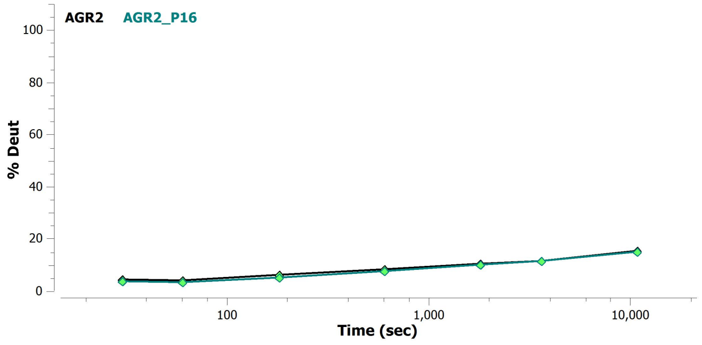

82-101: IIHHLDECPHSQALKKVFAE

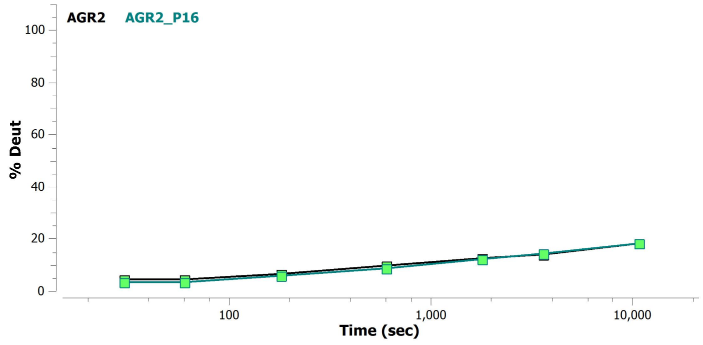

82-102: IIHHLDECPHSQALKKVFAEN

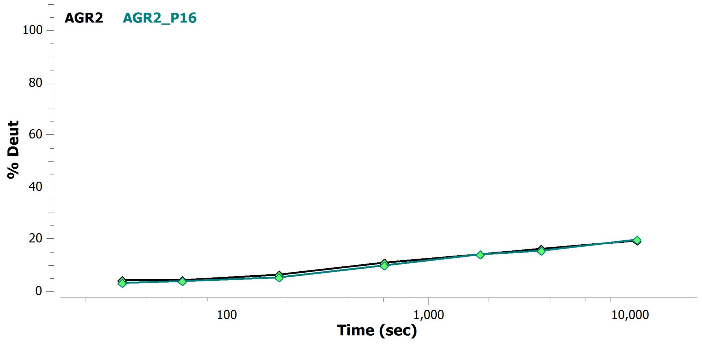

82-104: IIHHLDECPHSQALKKVFAENKE

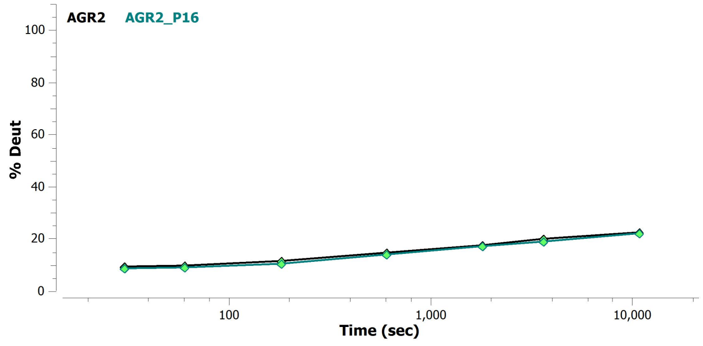

82-108: IIHHLDECPHSQALKKVFAENKEIQKL

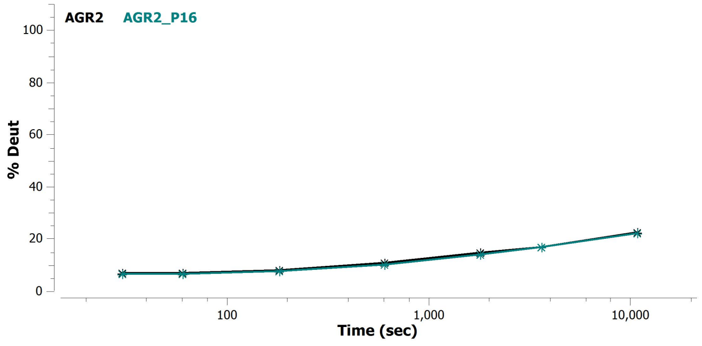

82-111: IIHHLDECPHSQALKKVFAENKEIQKLAEQ

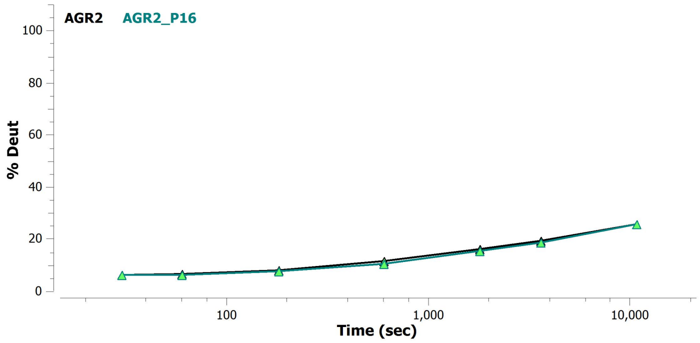

82-112: IIHHLDECPHSQALKKVFAENKEIQKLAEQF

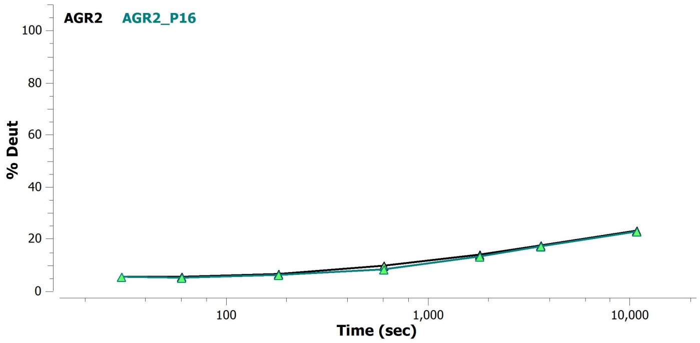

82-114: IIHHLDECPHSQALKKVFAENKEIQKLAEQFVL

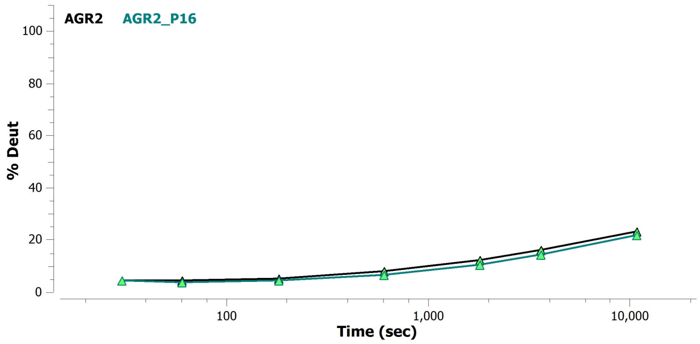

82-117: IIHHLDECPHSQALKKVFAENKEIQKLAEQFVLLNL

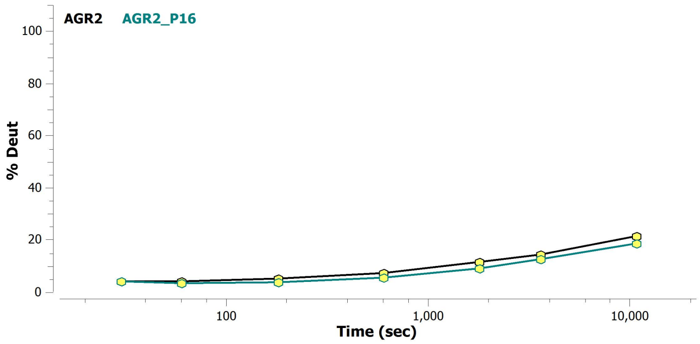

95-104: LKKVFAENKE

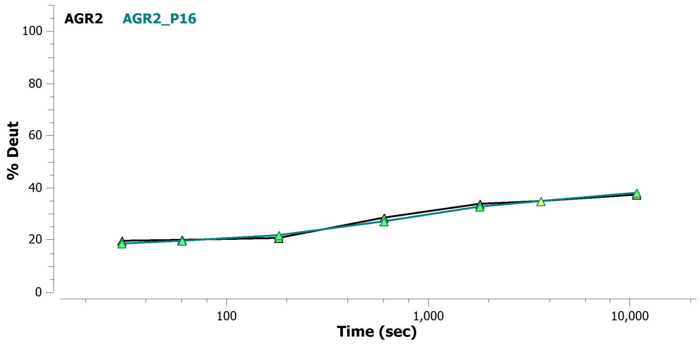

95-111: LKKVFAENKEIQKLAEQ

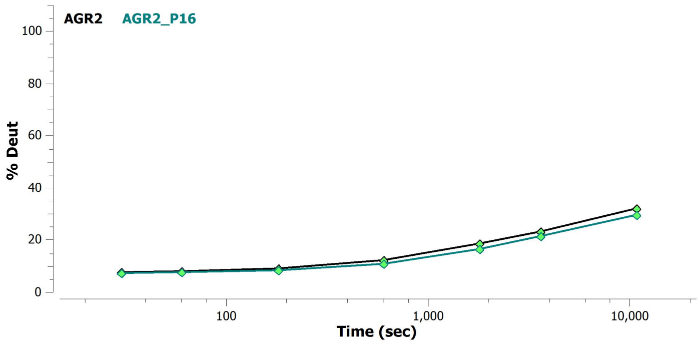

95-112: LKKVFAENKEIQKLAEQF

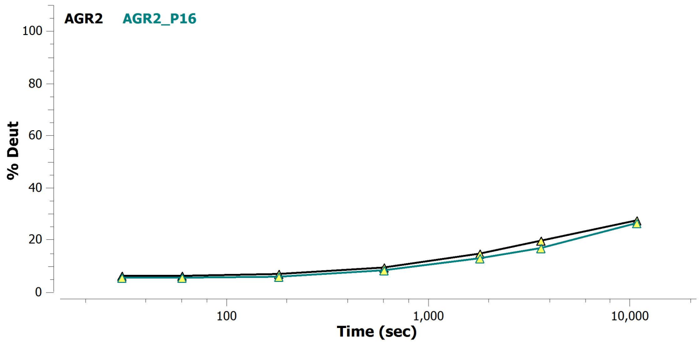

100-111: AENKEIQKLAEQ

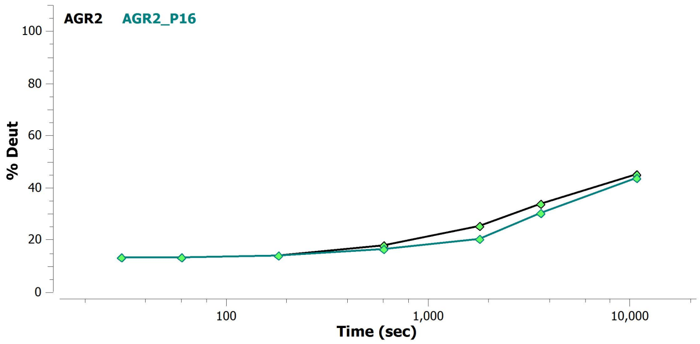

100-112: AENKEIQKLAEQF

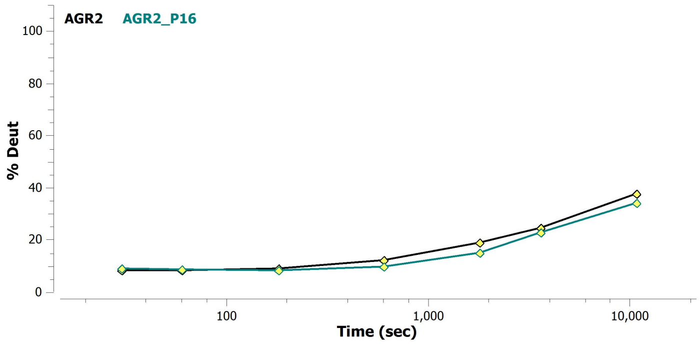

102-111: NKEIQKLAEQ

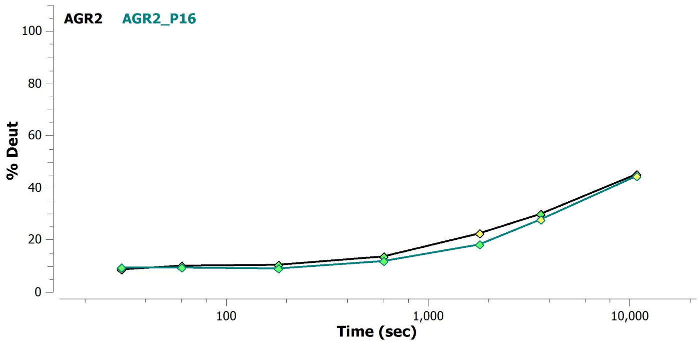

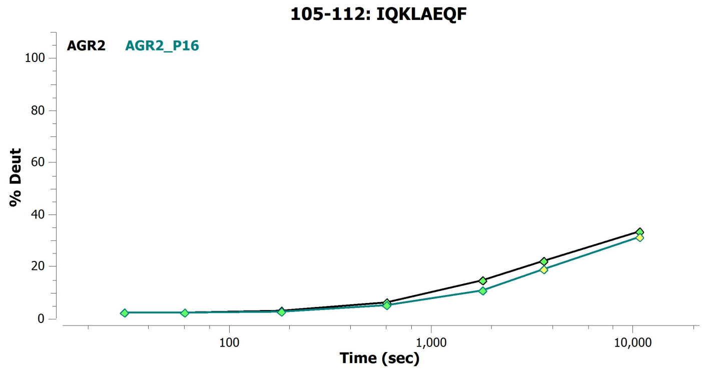
105-112: IQKLAEQF
AGR2
AGR2_P16
% Deut
0
20
40
60
80
100
100
1,000
10,000
Time (sec)

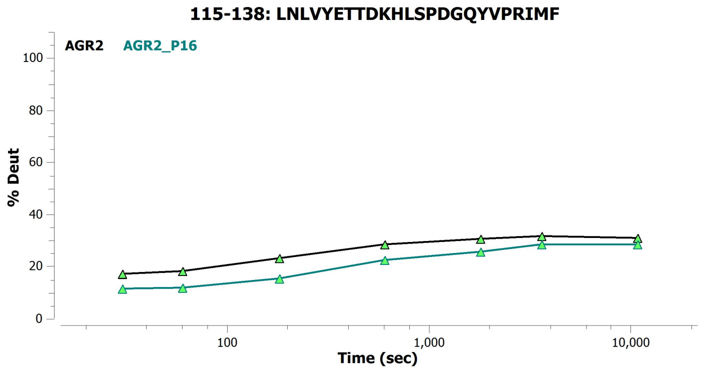
115-138: LNLVYETTDKHLSPDGQYVPRIMF
AGR2
AGR2_P16
% Deut
0
20
40
60
80
100
100
1,000
10,000
Time (sec)

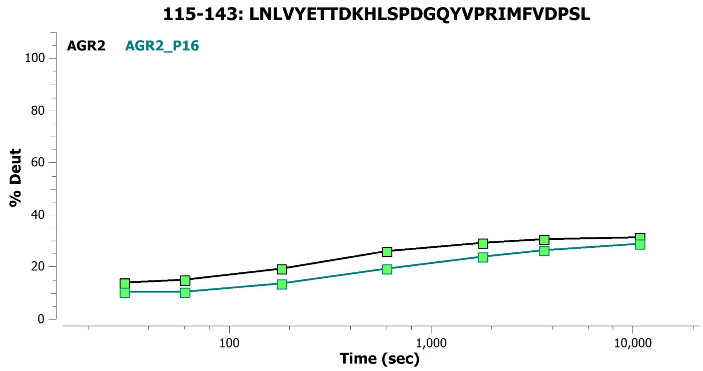
115-143: LNLVYETTDKHLSPDGQYVPRIMFVDPSL
AGR2
AGR2_P16
% Deut
0
20
40
60
80
100
100
1,000
10,000
Time (sec)

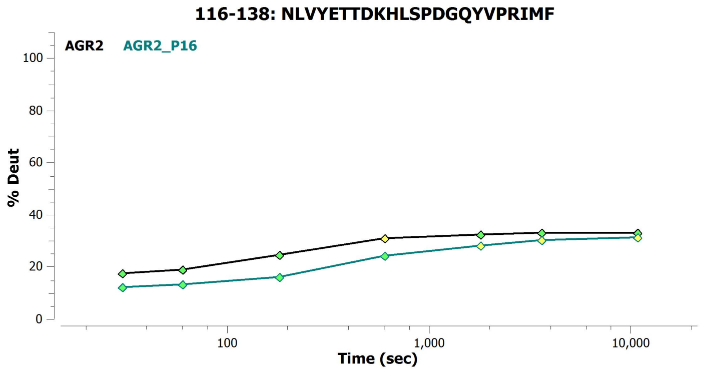
116-138: NLVYETTDKHLSPDGQYVPRIMF
AGR2
AGR2_P16
% Deut
0
20
40
60
80
100
100
1,000
10,000
Time (sec)

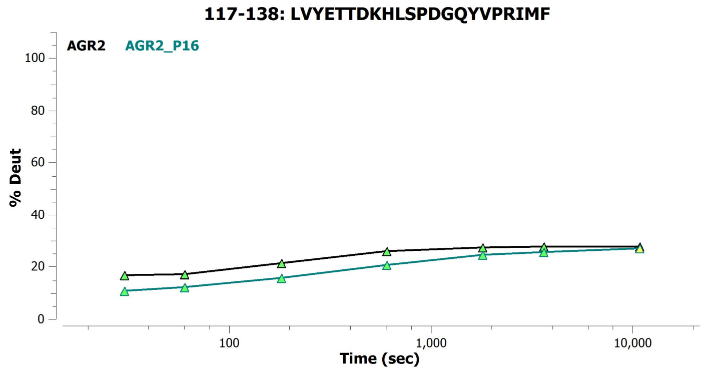
117-138: LVYETTDKHLSPDGQYVPRIMF
AGR2
AGR2_P16
% Deut
0
20
40
60
80
100
100
1,000
10,000
Time (sec)

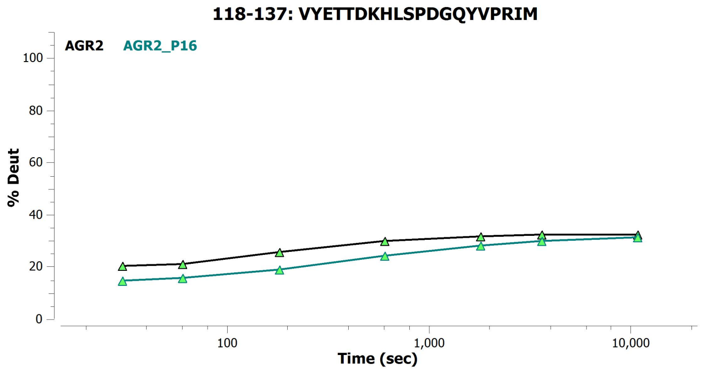
118-137: VYETTDKHLSPDGQYVPRIM
AGR2
AGR2_P16
% Deut
0
20
40
60
80
100
100
1,000
10,000
Time (sec)

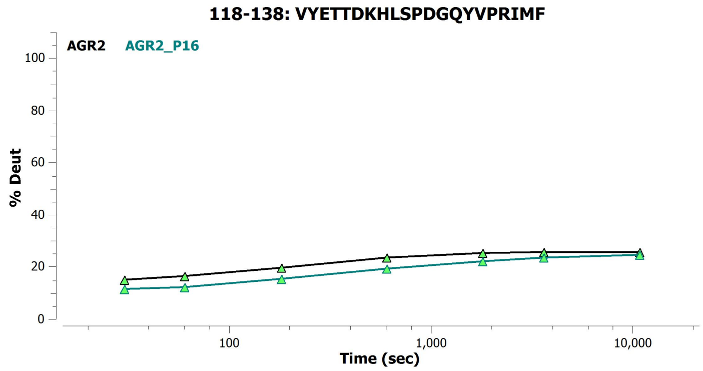
118-138: VYETTDKHLSPDGQYVPRIMF
AGR2
AGR2_P16
% Deut
0
20
40
60
80
100
100
1,000
10,000
Time (sec)

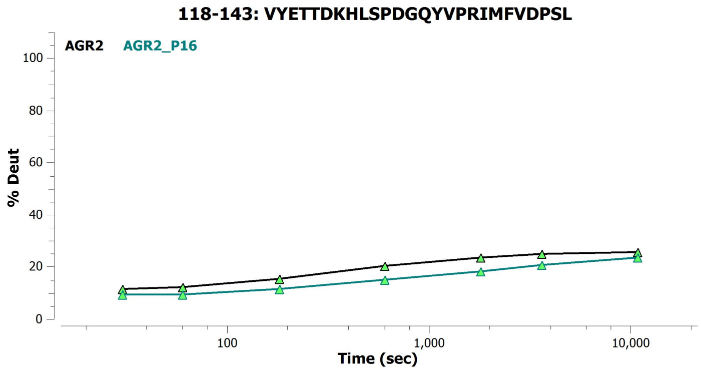
118-143: VYETTDKHLSPDGQYVPRIMFVDPSL
AGR2
AGR2_P16
% Deut
0
20
40
60
80
100
100
1,000
10,000
Time (sec)

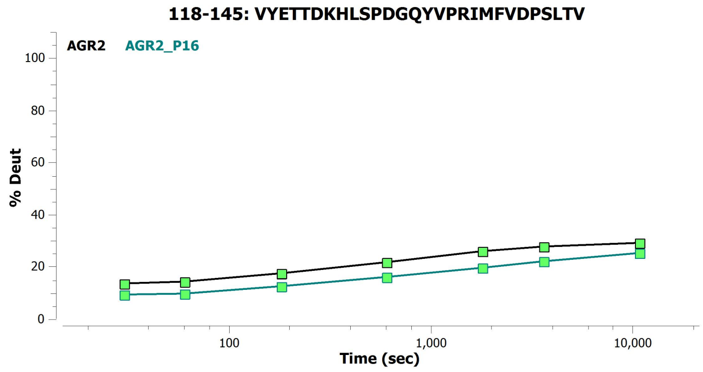
118-145: VYETTDKHLSPDGQYVPRIMFVDPSLTV
AGR2
AGR2_P16
% Deut
0
20
40
60
80
100
100
1,000
10,000
Time (sec)

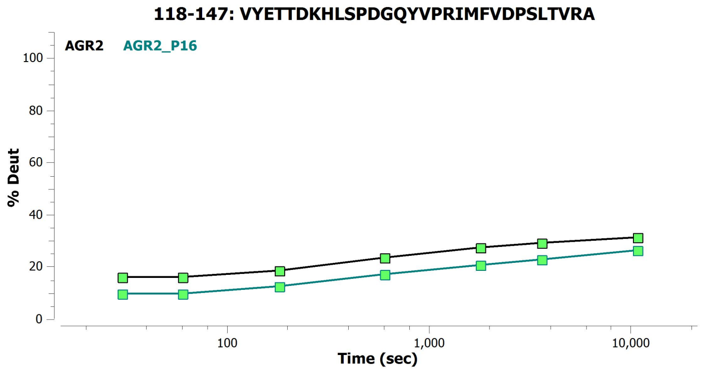
118-147: VYETTDKHLSPDGQYVPRIMFVDPSLTVRA
AGR2
AGR2_P16
% Deut
0
20
40
60
80
100
100
1,000
10,000
Time (sec)

118-157: VYETTDKHLSPDGQYVPRIMFVDPSLTVRADITGRYSNRL

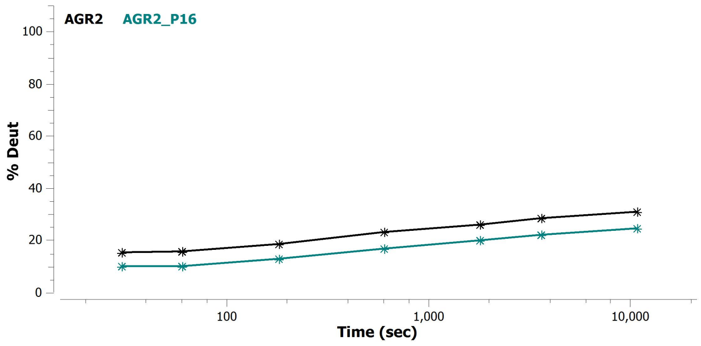

119-138: YETTDKHLSPDGQYVPRIMF

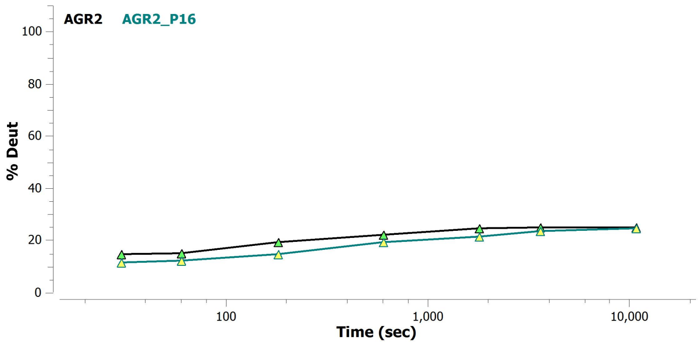

120-138: ETTDKHLSPDGQYVPRIMF

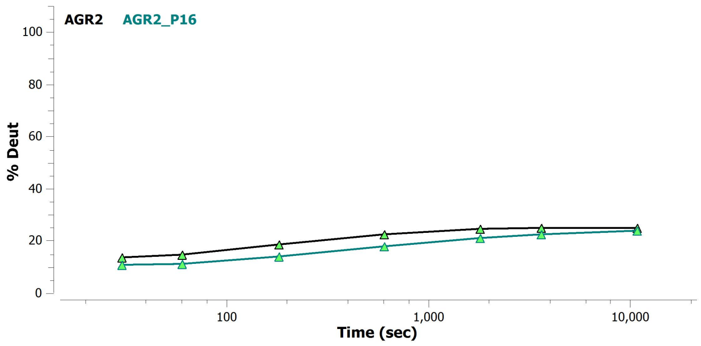

139-147: VDPSLTVRA

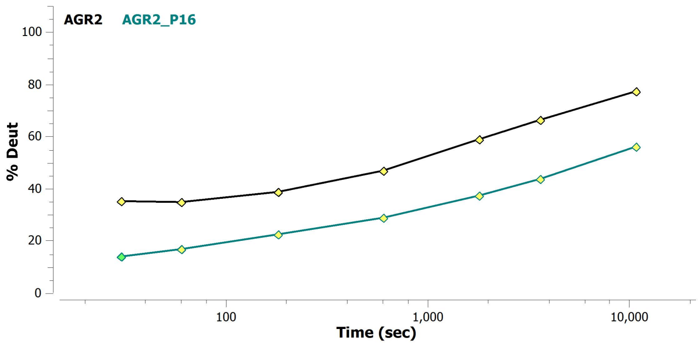

144-157: TVRADITGRYSNRL

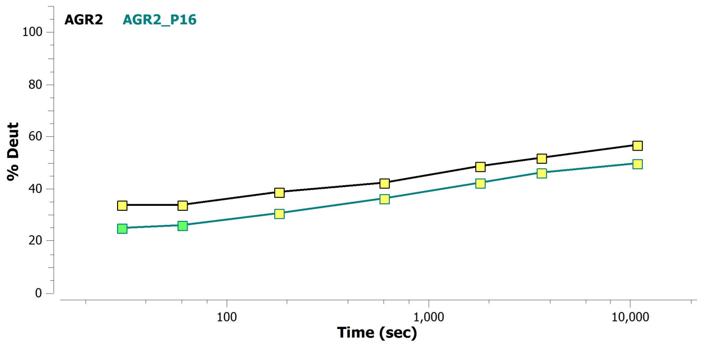

144-183: TVRADITGRYSNRLYAYEPADTALLLDNMKKALKLLKTEL

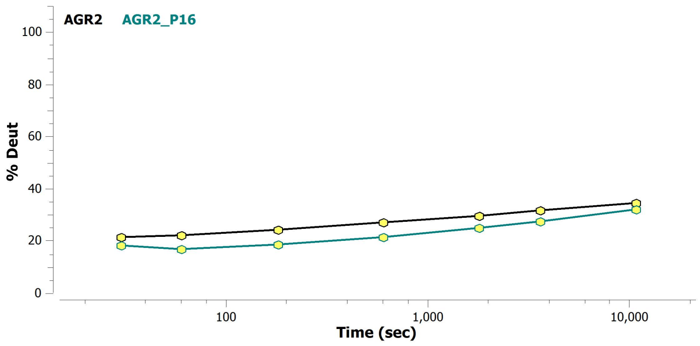

145-157: VRADITGRYSNRL

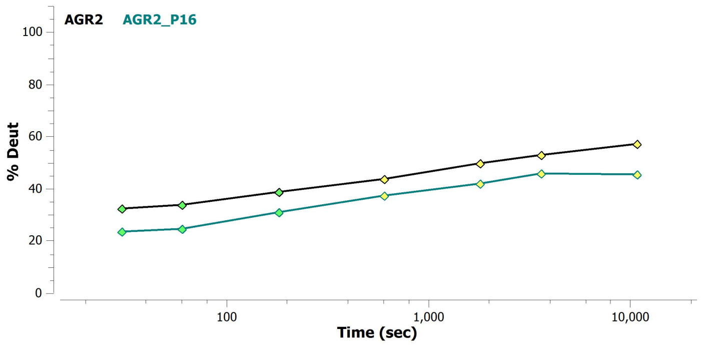

146-157: RADITGRYSNRL

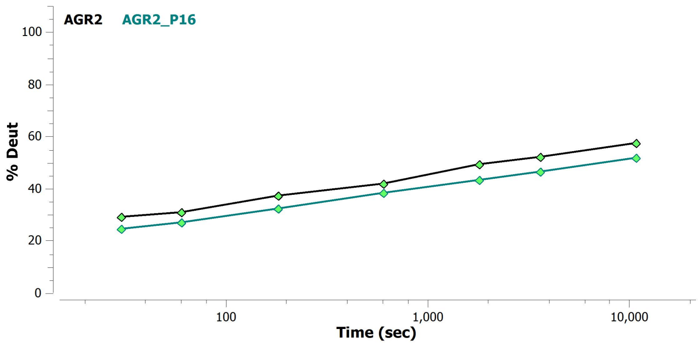

158-167: YAYEPADTAL

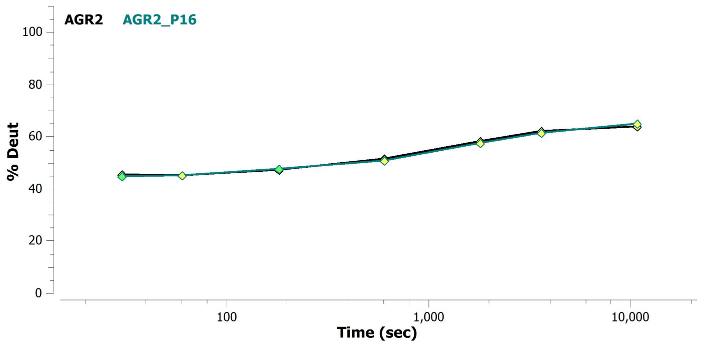

158-168: YAYEPADTALL

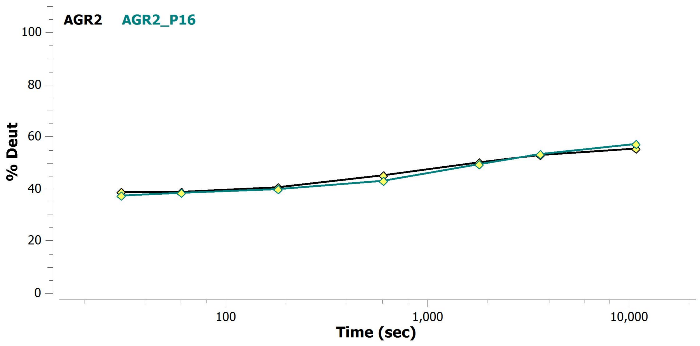

158-178: YAYEPADTALLLDNMKKALKL

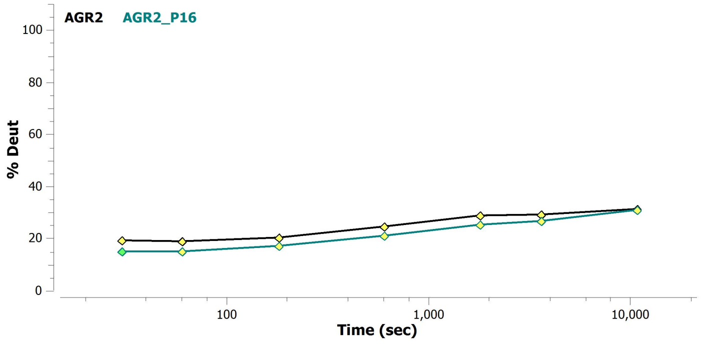

158-183: YAYEPADTALLLDNMKKALKLLKTEL

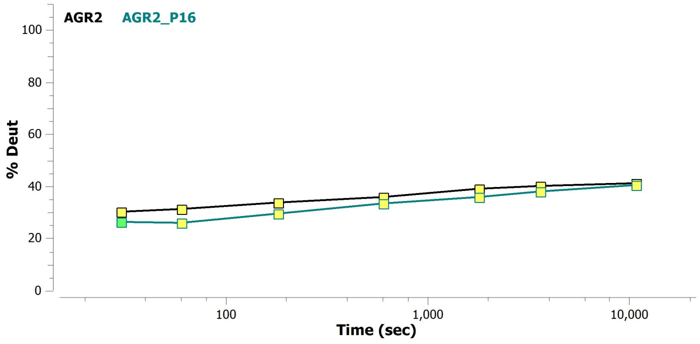

168-178: LLDNMKKALKL

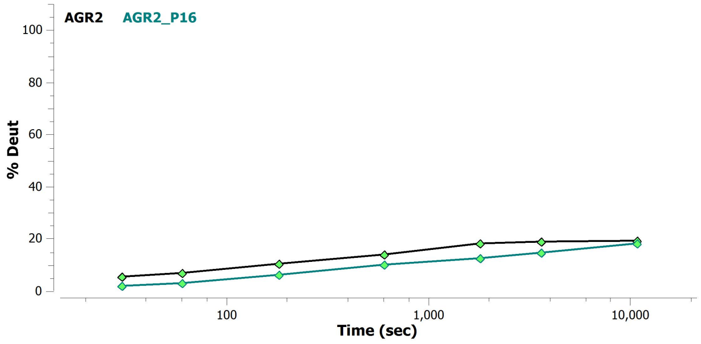

168-182: LLDNMKKALKLLKTE

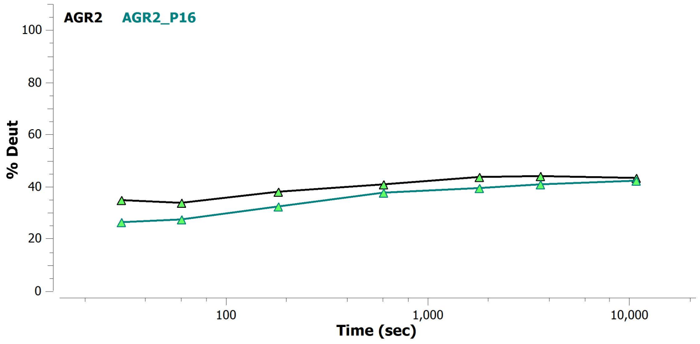

168-183: LLDNMKKALKLLKTEL

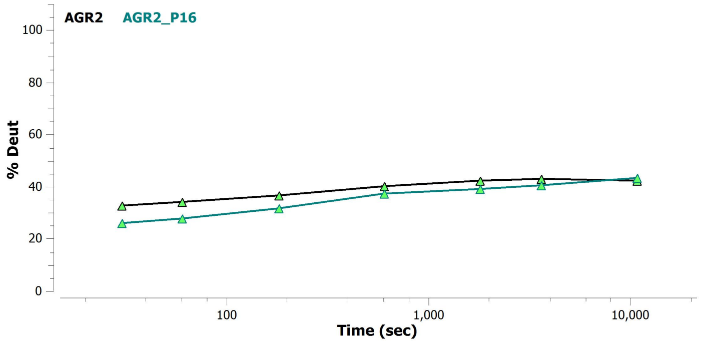

169-178: LDNMKKALKL

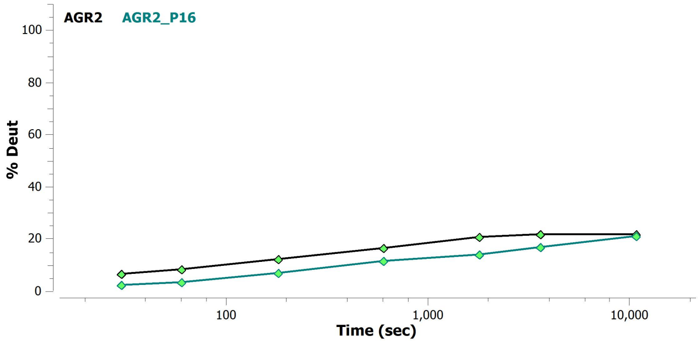

169-183: LDNMKKALKLLKTEL

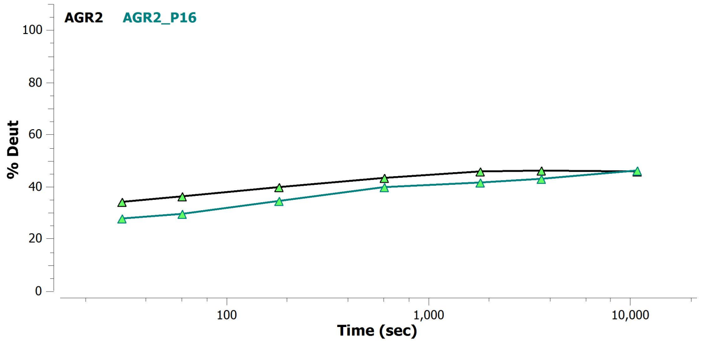

170-183: DNMKKALKLLKTEL

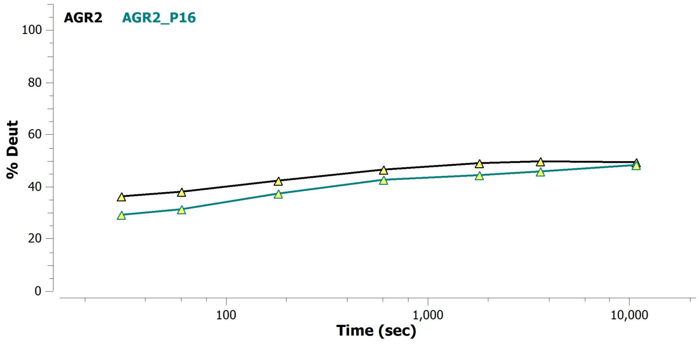

173-183: KKALKLLKTEL

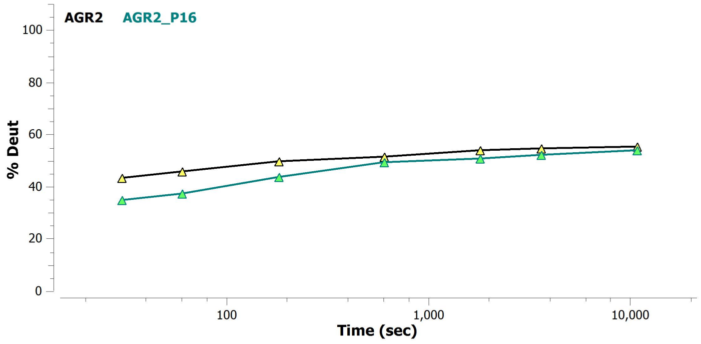

Supplement: Supplemental Data [file supp_RA118.000573_134890_0_supp_50209_p258z0.pdf]
