# Supplementary material for: The Sequence-specific Peptide-binding Activity of the Protein Sulfide Isomerase AGR2 Directs Its Stable Binding to the Oncogenic Receptor EpCAM
Source: Mol Cell Proteomics. 2018 Jan 16;17(4):737–63. doi: 10.1074/mcp.RA118.000573 (PMC5880107; doi:10.1074/mcp.RA118.000573)
Supplement: Supplemental Data [file supp_RA118.000573_134890_0_supp_50207_p258tz.pdf]

2-13: SYYHHHHHHLES

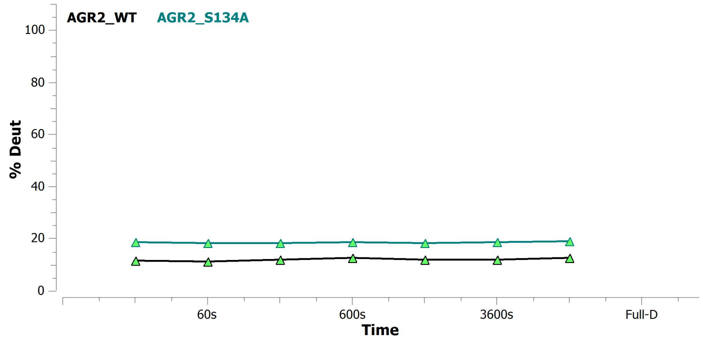

2-16: SYYHHHHHHLESTSL

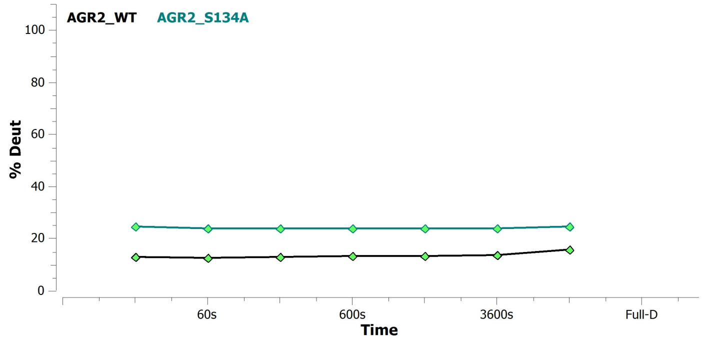

17-30: YKKAGFEGDRTMRD

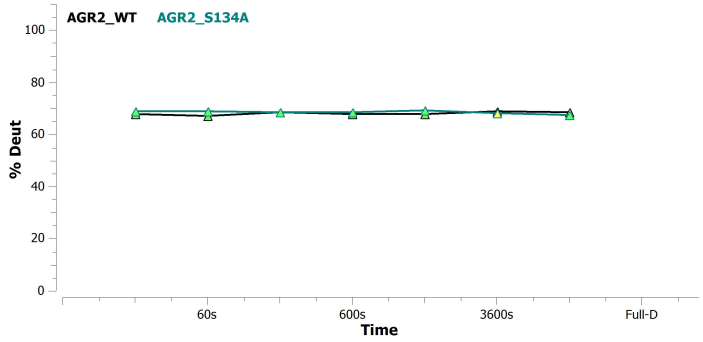

17-52: YKKAGFEGDRTMRDTTVKPGAKKDTKDSRPKLPQTL

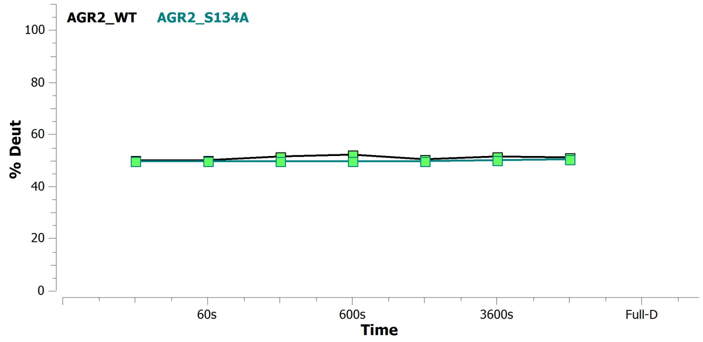

17-59: YKKAGFEGDRTMRDTTVKPGAKKDTKDSRPKLPQTLSRGWGDQ

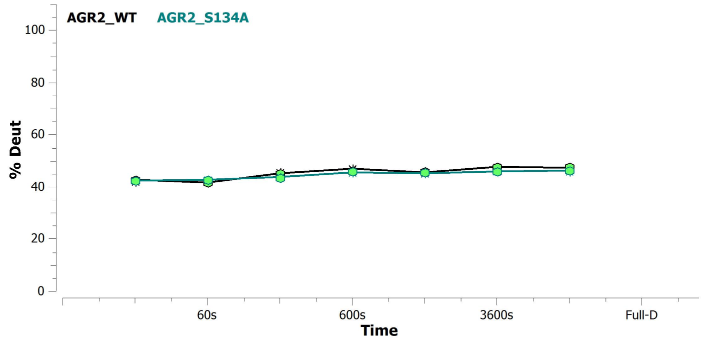

17-60: YKKAGFEGDRTMRDTTVKPGAKKDTKDSRPKLPQTLSRGWGDQL

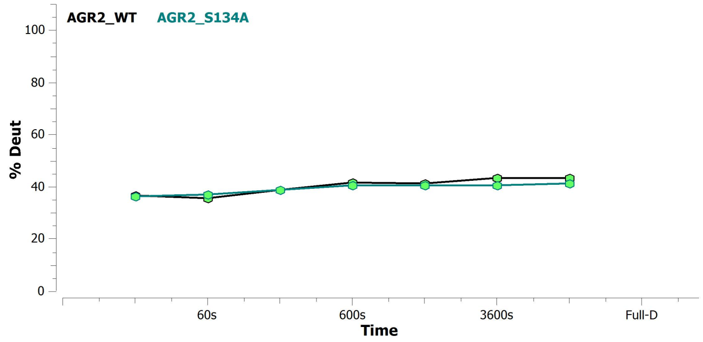

18-30: KKAGFEGDRTMRD

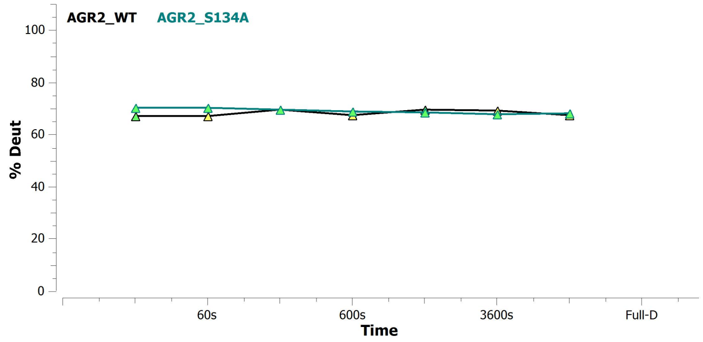

23-52: EGDRTMRDTTVKPGAKKDTKDSRPKLPQTL

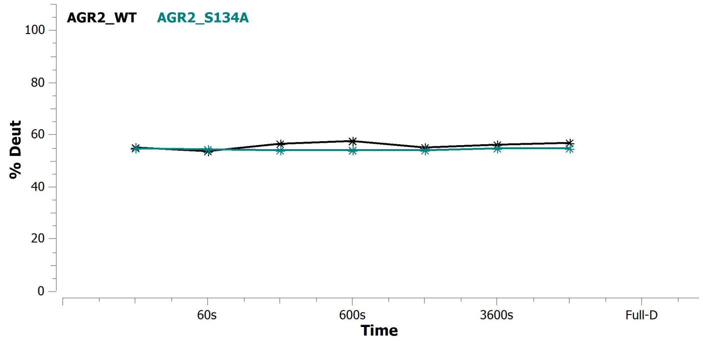

23-60: EGDRTMRDTTVKPGAKKDTKDSRPKLPQTLSRGWGDQL

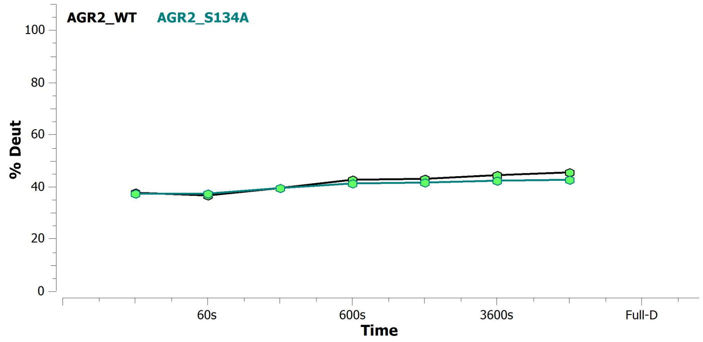

25-52: DRTMRDTTVKPGAKKDTKDSRPKLPQTL

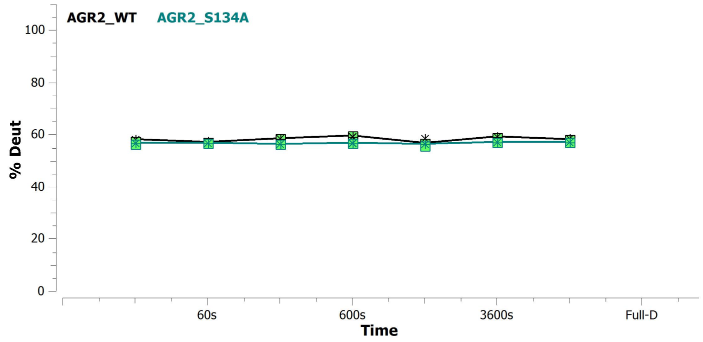

25-60: DRTMRDTTVKPGAKKDTKDSRPKLPQTLSRGWGDQL

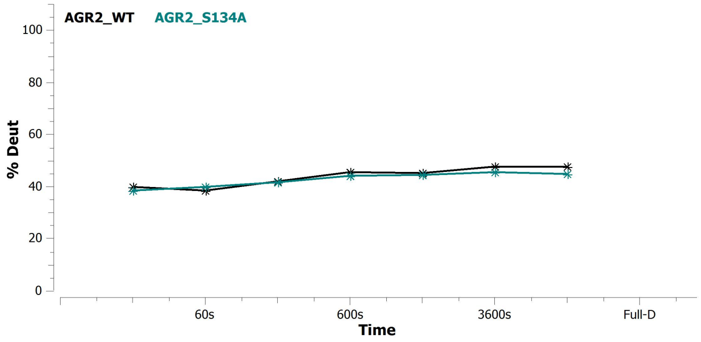

26-52: RTMRDTTVKPGAKKDTKDSRPKLPQTL

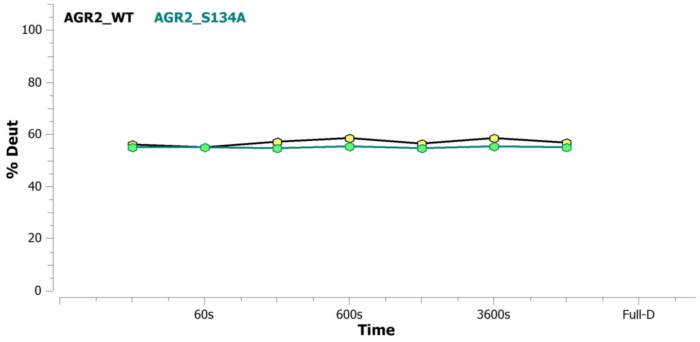

28-52: MRDTTVKPGAKKDTKDSRPKLPQTL

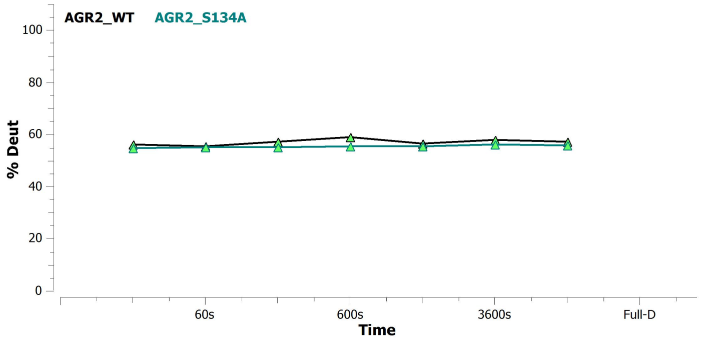

28-59: MRDTTVKPGAKKDTKDSRPKLPQTLSRGWGDQ

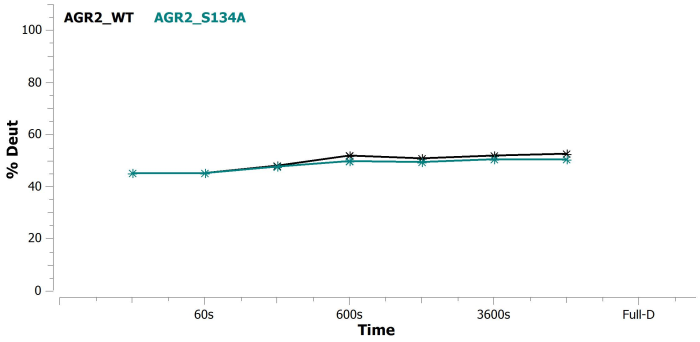

28-60: MRDTTVKPGAKKDTKDSRPKLPQTLSRGWGDQL

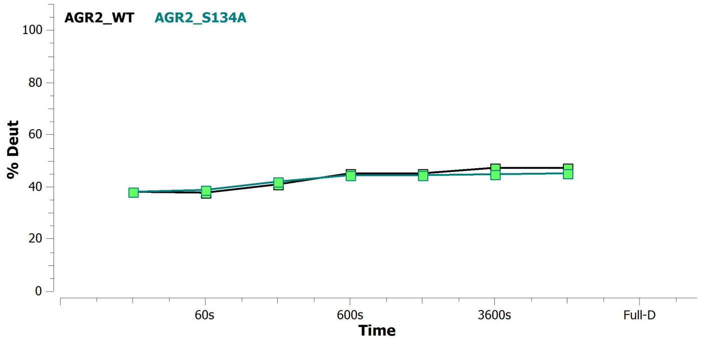

28-65: MRDTTVKPGAKKDTKDSRPKLPQTLSRGWGDQLIWTQT

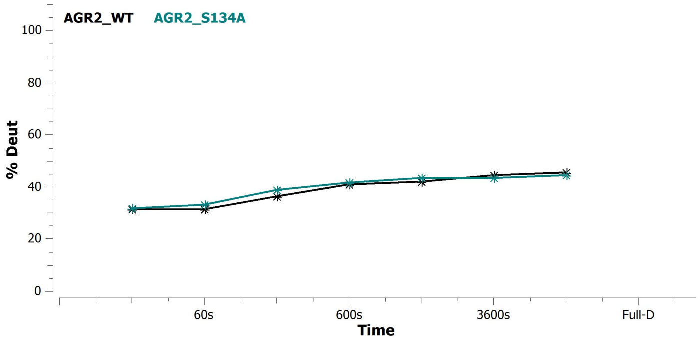

31-51: TTVKPGAKKDTKDSRPKLPQT

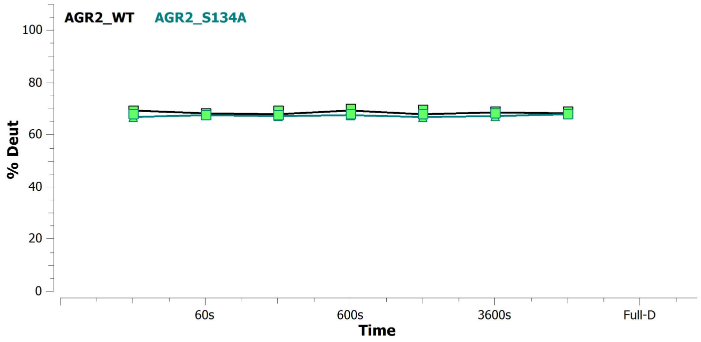

31-52: TTVKPGAKKDTKDSRPKLPQTL

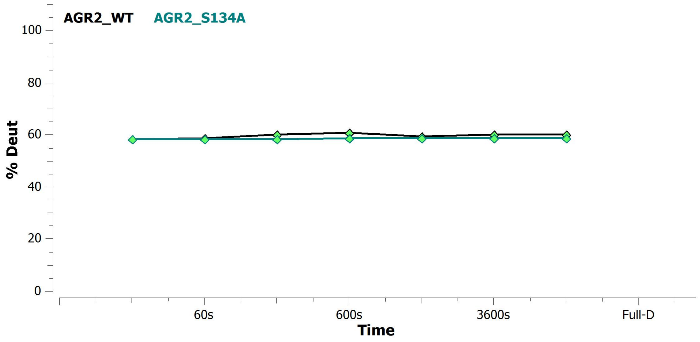

31-55: TTVKPGAKKDTKDSRPKLPQTLSRG

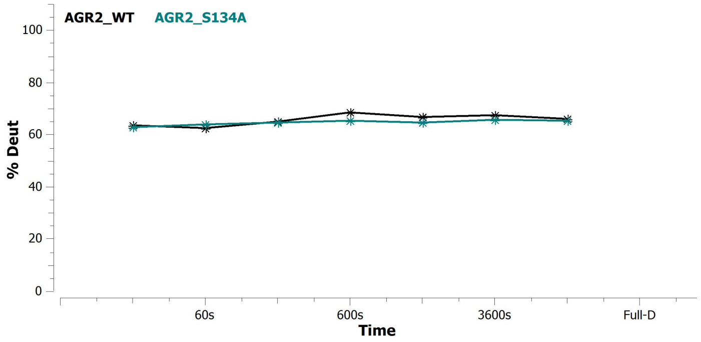

31-56: TTVKPGAKKDTKDSRPKLPQTLSRGW

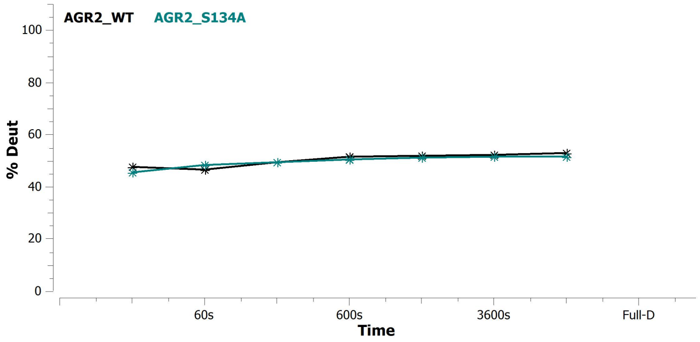

31-59: TTVKPGAKKDTKDSRPKLPQTLSRGWGDQ

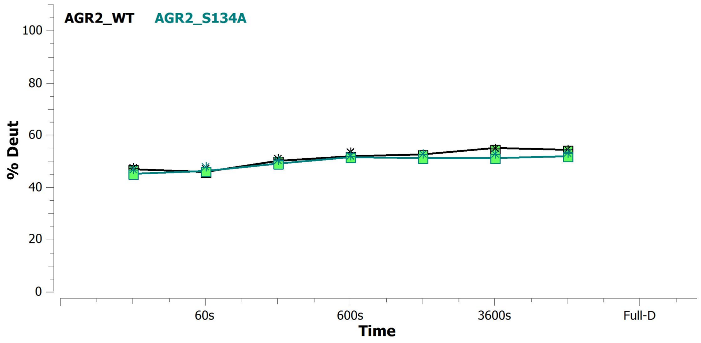

31-60: TTVKPGAKKDTKDSRPKLPQTLSRGWGDQL

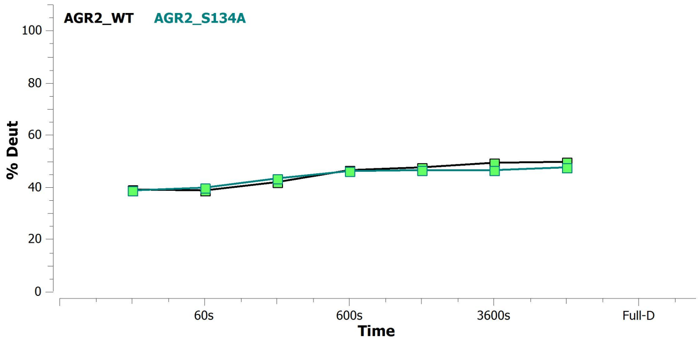

49-60: PQTLSRGWGDQL

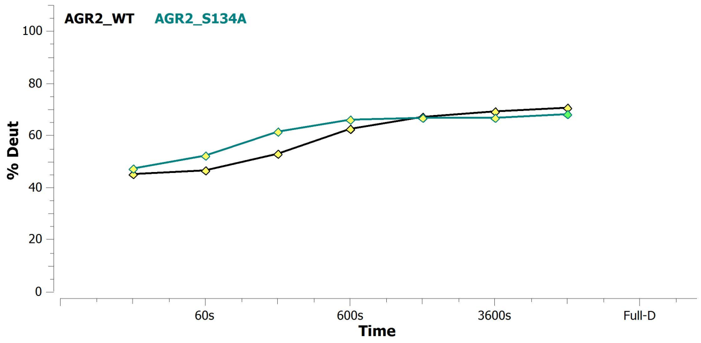

52-60: LSRGWGDQL

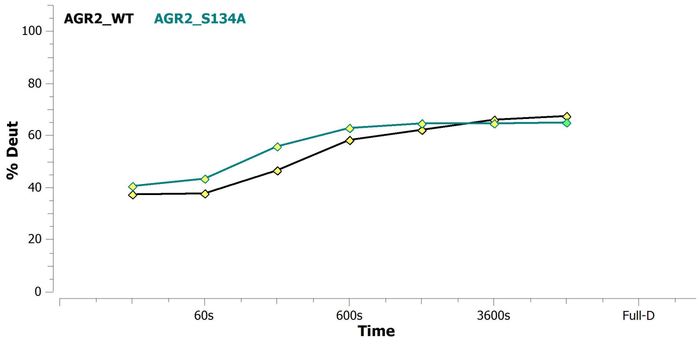

53-59: SRGWGDQ

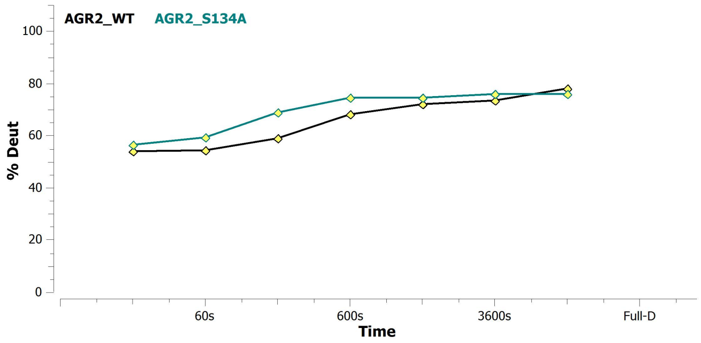

53-60: SRGWGDQL

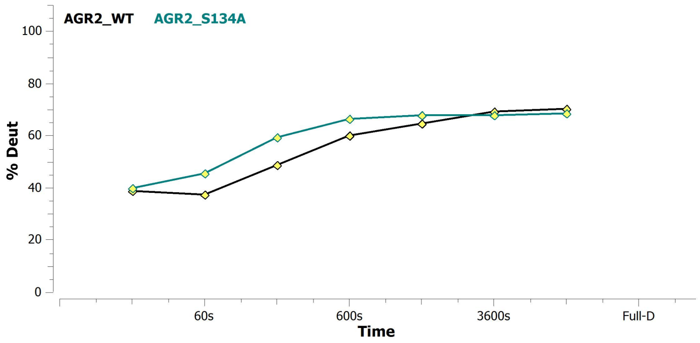

53-65: SRGWGDQLIWTQT

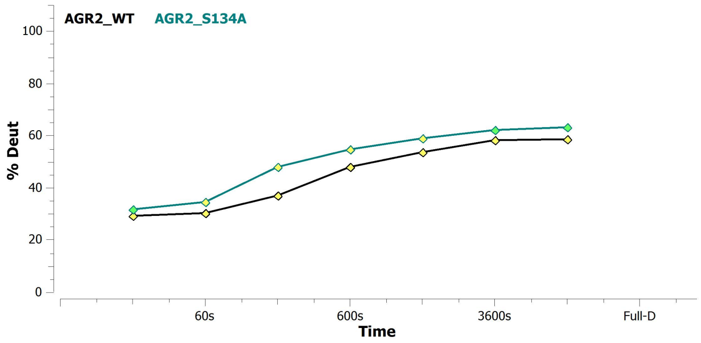

60-67: LIWTQTYE

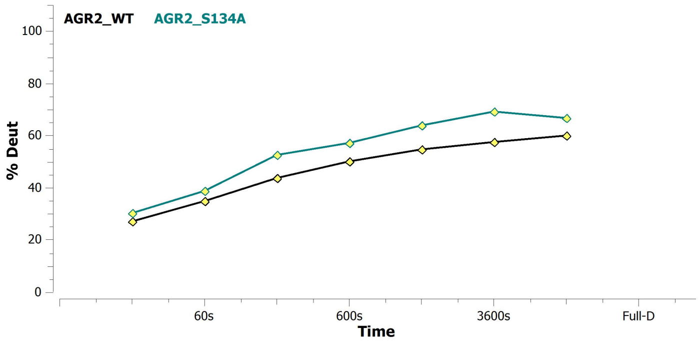

66-81: YEEALYKSKTSSKPLM

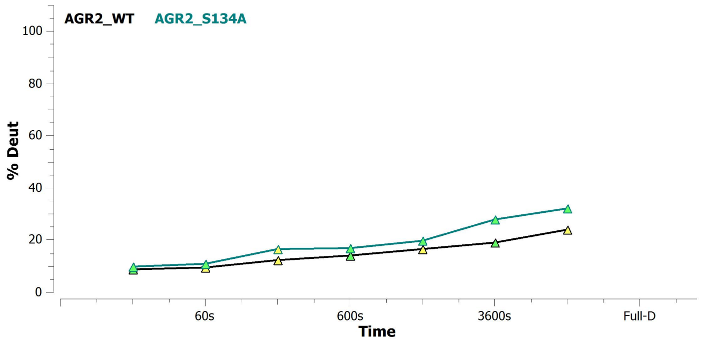

69-111: ALYKSKTSSKPLMIIHHLDECPHSQALKKVFAENKEIQKLAEQ

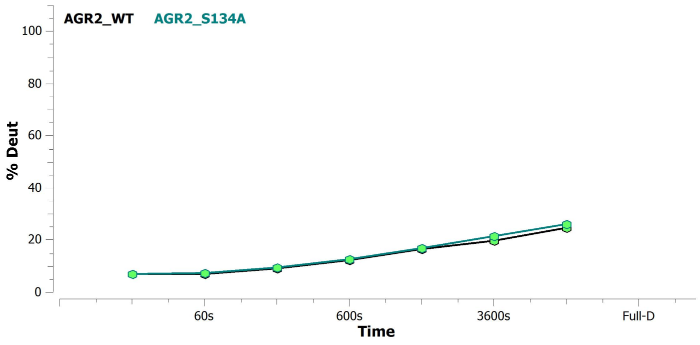

70-81: LYKSKTSSKPLM

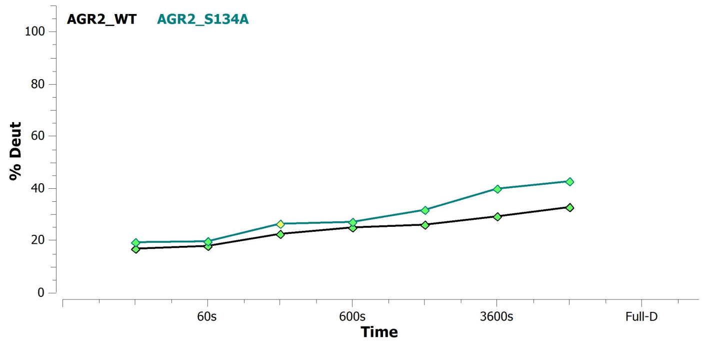

70-111: LYKSKTSSKPLMIIHHLDECPHSQALKKVFAENKEIQKLAEQ

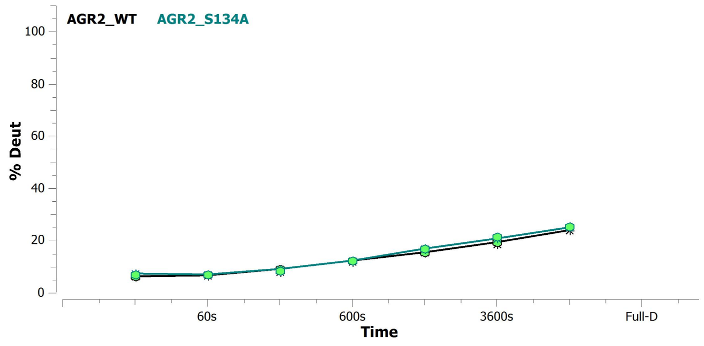

71-94: YKSKTSSKPLMIIHHLDECPHSQA

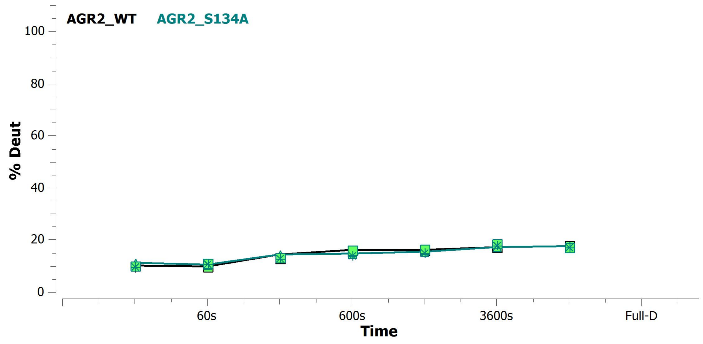

71-95: YKSKTSSKPLMIIHHLDECPHSQAL

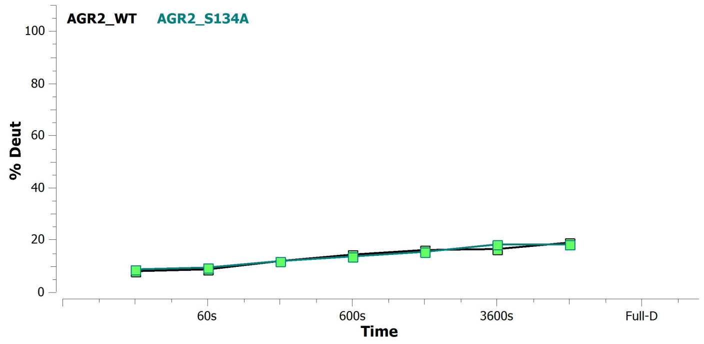

71-101: YKSKTSSKPLMIIHHLDECPHSQALKKVFAE

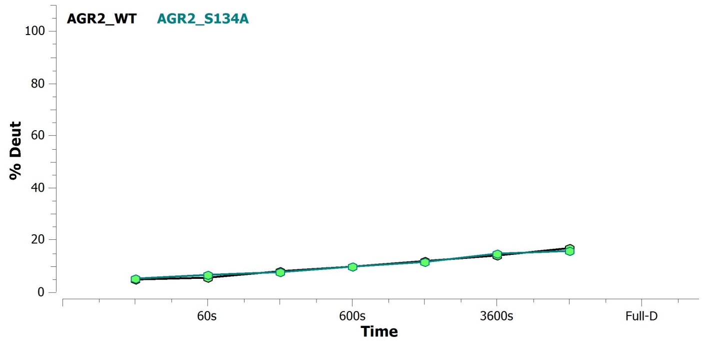

71-102: YKSKTSSKPLMIIHHLDECPHSQALKKVFAEN

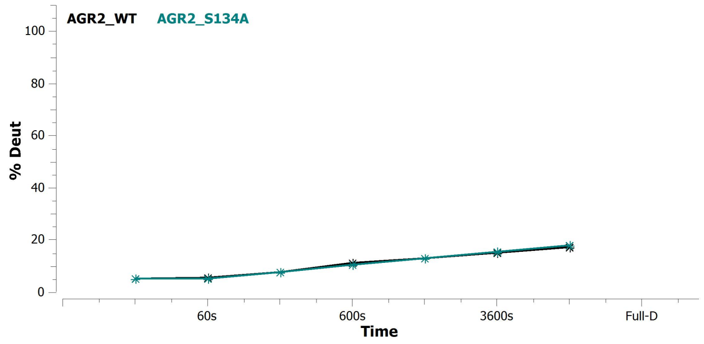

71-104: YKSKTSSKPLMIIHHLDECPHSQALKKVFAENKE

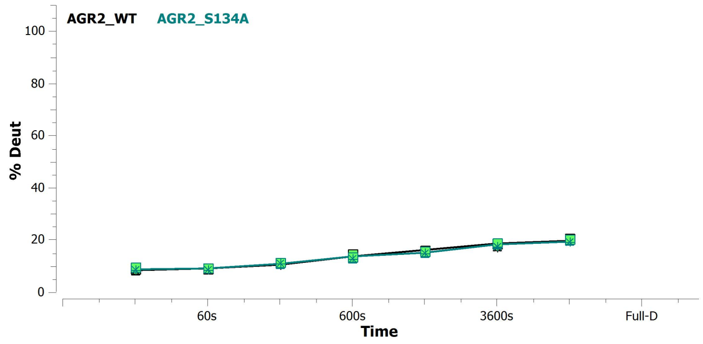

71-108: YKSKTSSKPLMIIHHLDECPHSQALKKVFAENKEIQKL

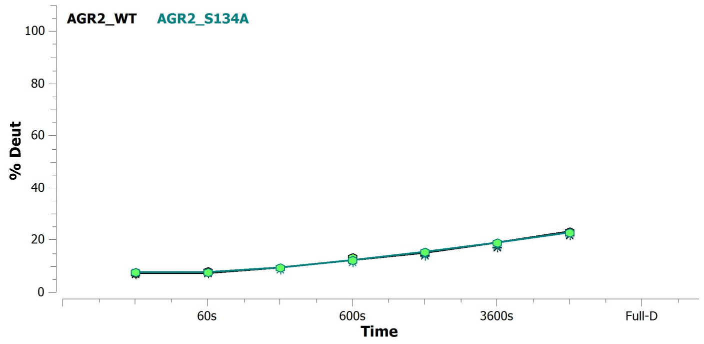

71-111: YKSKTSSKPLMIIHHLDECPHSQALKKVFAENKEIQKLAEQ

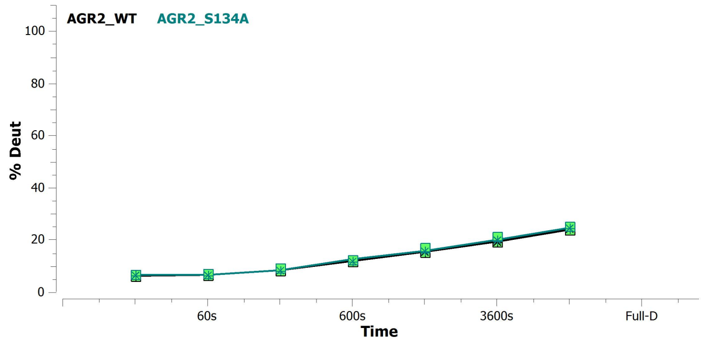

71-112: YKSKTSSKPLMIIHHLDECPHSQALKKVFAENKEIQKLAEQF

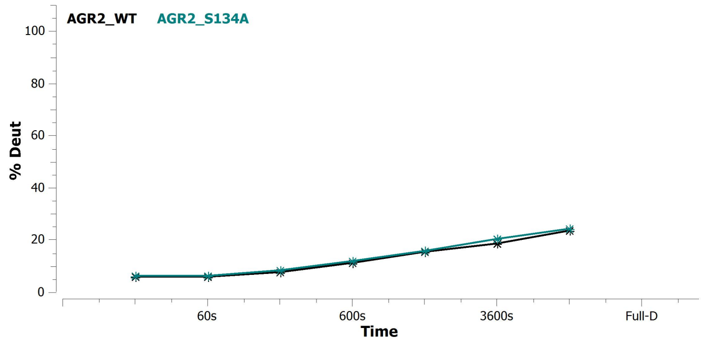

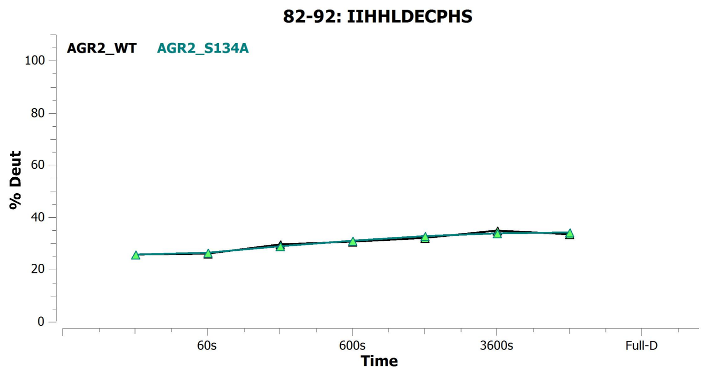
82-92: IIHHLDECPHS
AGR2_WT
AGR2_S134A
% Deut
Time
60s
600s
3600s
Full-D

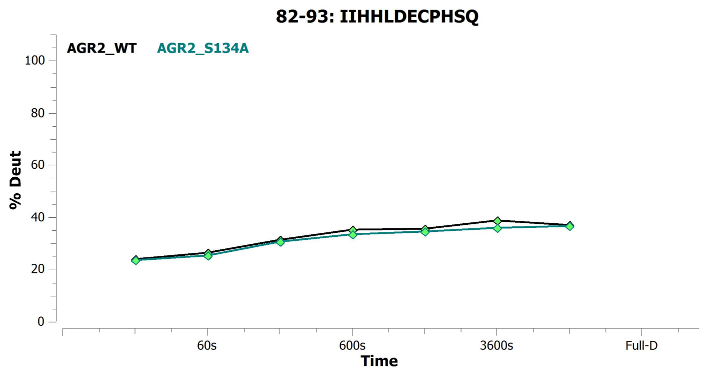
82-93: IIHHLDECPHSQ
AGR2_WT
AGR2_S134A
% Deut
Time
60s
600s
3600s
Full-D

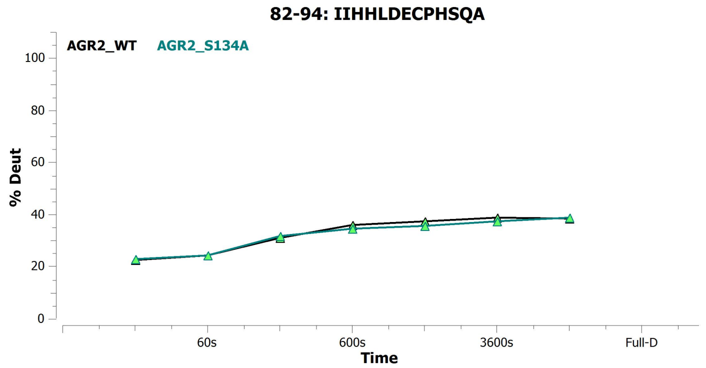
82-94: IIHHLDECPHSQA
AGR2_WT
AGR2_S134A
% Deut
Time
60s
600s
3600s
Full-D

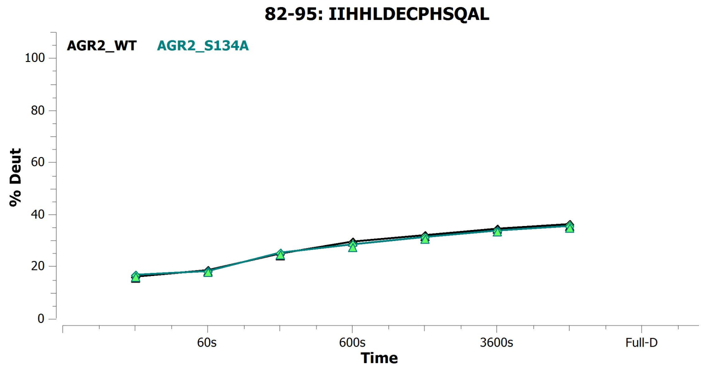
82-95: IIHHLDECPHSQAL
AGR2_WT
AGR2_S134A
% Deut
Time
60s
600s
3600s
Full-D

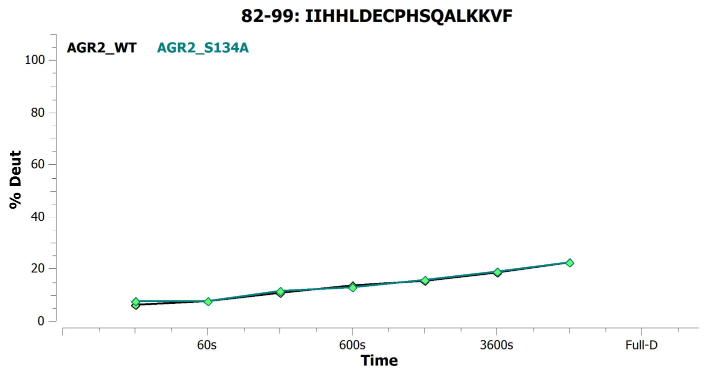
82-99: IIHHLDECPHSQALKKVF
AGR2_WT
AGR2_S134A
% Deut
Time
60s
600s
3600s
Full-D

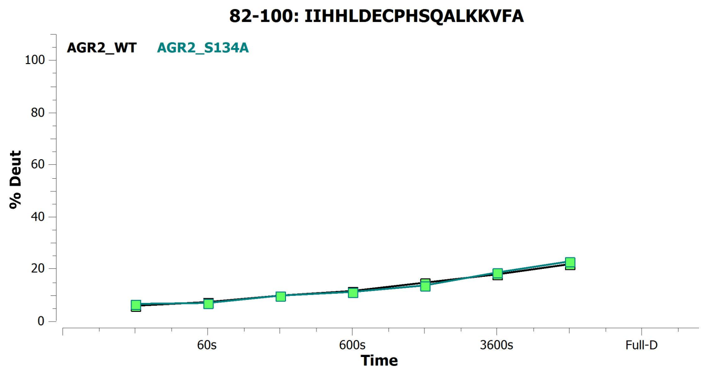
82-100: IIHHLDECPHSQALKKVFA
AGR2_WT
AGR2_S134A
% Deut
Time
60s
600s
3600s
Full-D

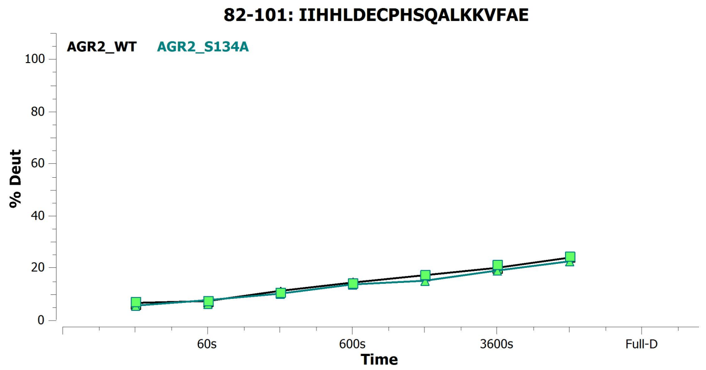
82-101: IIHHLDECPHSQALKKVFAE
AGR2_WT
AGR2_S134A
% Deut
Time
60s
600s
3600s
Full-D

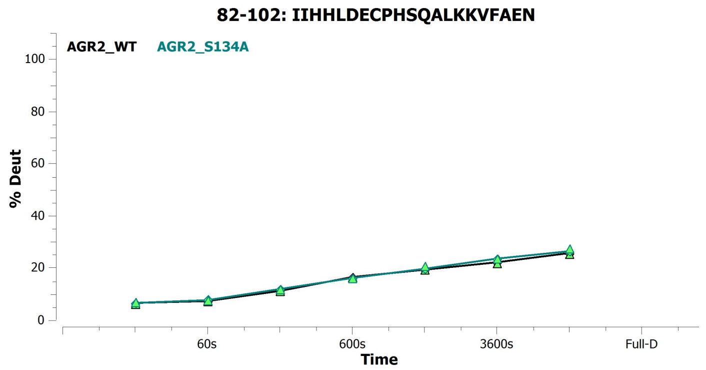
82-102: IIHHLDECPHSQALKKVFAEN
AGR2_WT
AGR2_S134A
% Deut
Time
60s
600s
3600s
Full-D

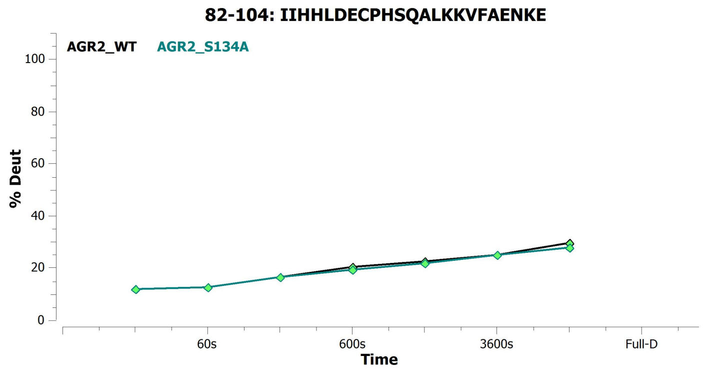
82-104: IIHHLDECPHSQALKKVFAENKE
AGR2_WT
AGR2_S134A
% Deut
Time
60s
600s
3600s
Full-D

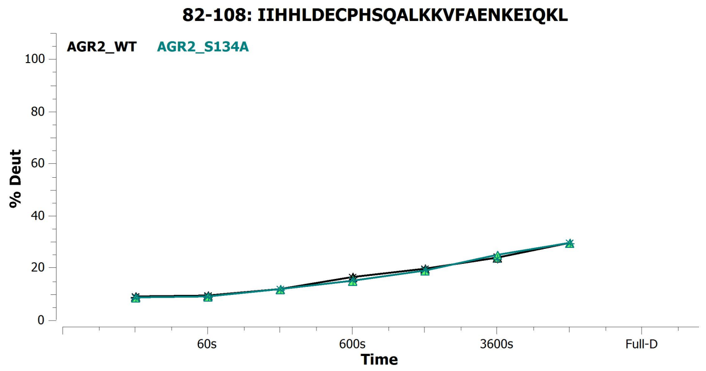
82-108: IIHHLDECPHSQALKKVFAENKEIQKL
AGR2_WT
AGR2_S134A
% Deut
Time
60s
600s
3600s
Full-D

82-111: IIHHLDECPHSQALKKVFAENKEIQKLAEQ

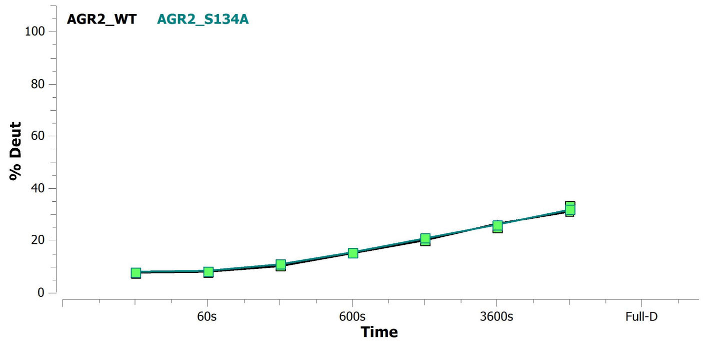

82-112: IIHHLDECPHSQALKKVFAENKEIQKLAEQF

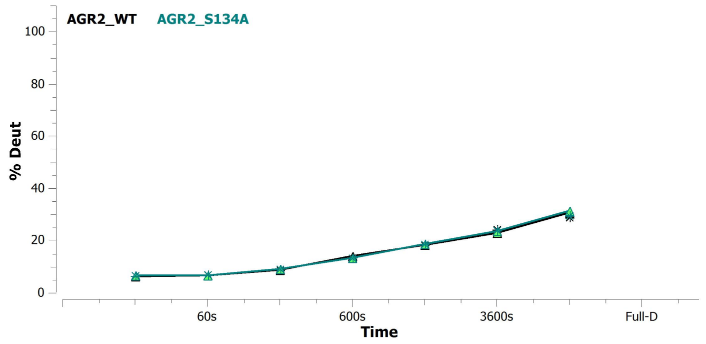

82-114: IIHHLDECPHSQALKKVFAENKEIQKLAEQFVL

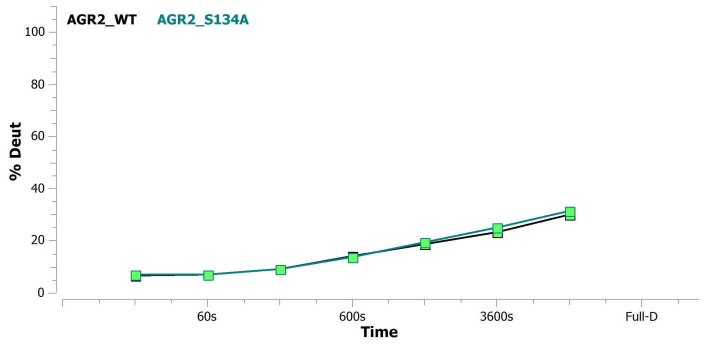

83-99: IHHLDECPHSQALKKVF

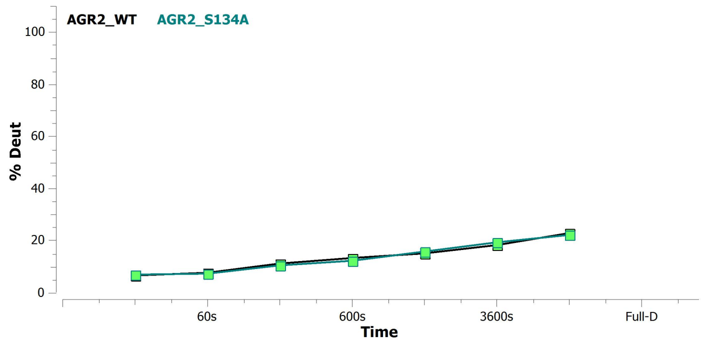

83-104: IHHLDECPHSQALKKVFAENKE

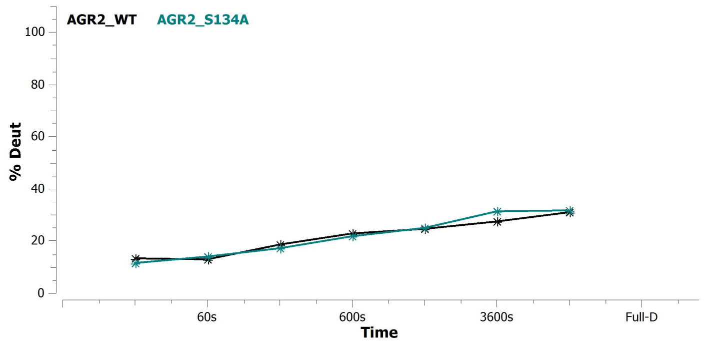

83-105: IHHLDECPHSQALKKVFAENKEI

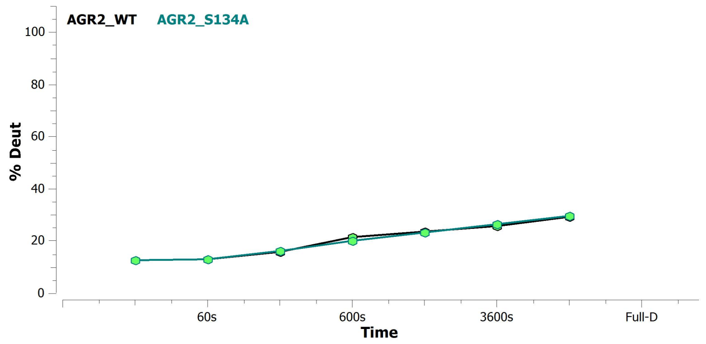

84-99: HHLDECPHSQALKKVF

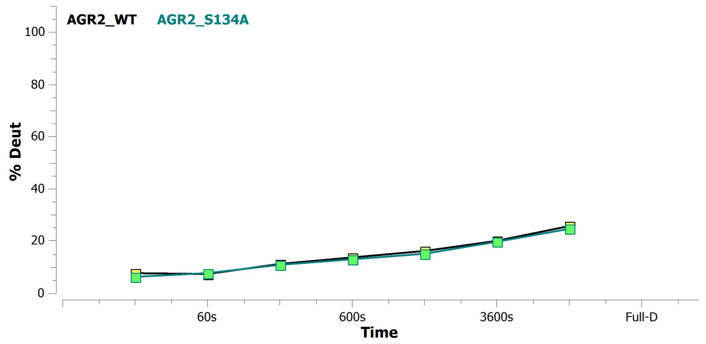

95-104: LKKVFAENKE

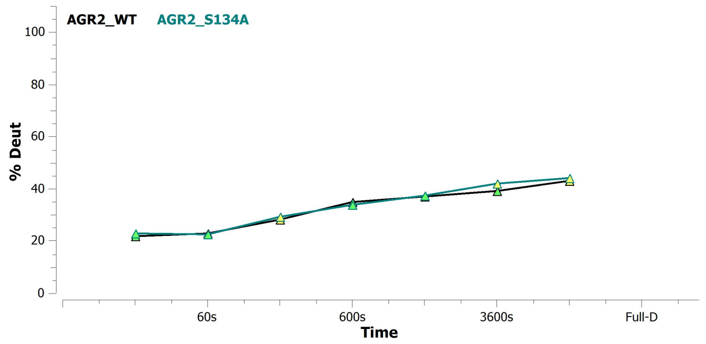

95-108: LKKVFAENKEIQKL

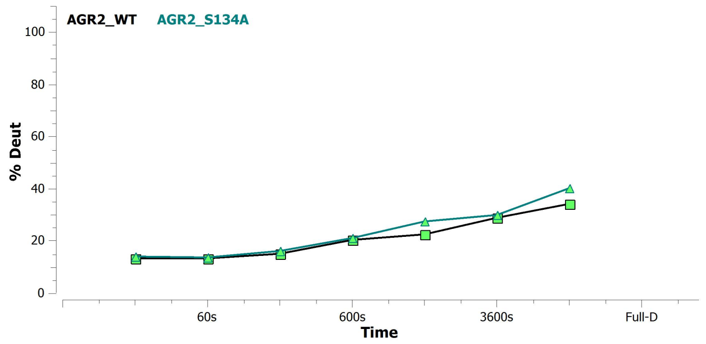

95-111: LKKVFAENKEIQKLAEQ

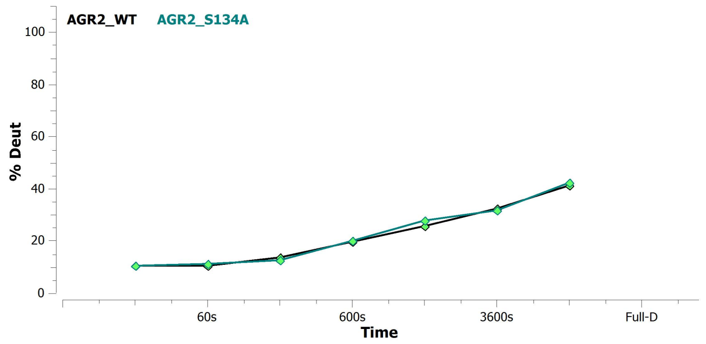

96-111: KKVFAENKEIQKLAEQ

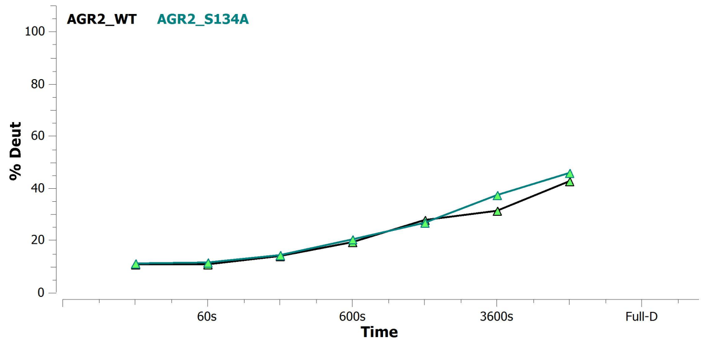

96-112: KKVFAENKEIQKLAEQF

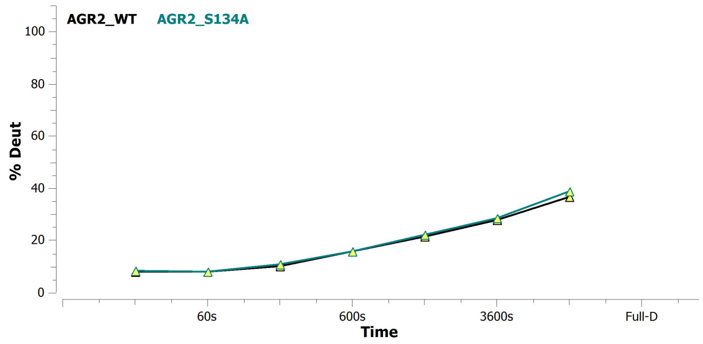

100-111: AENKEIQKLAEQ

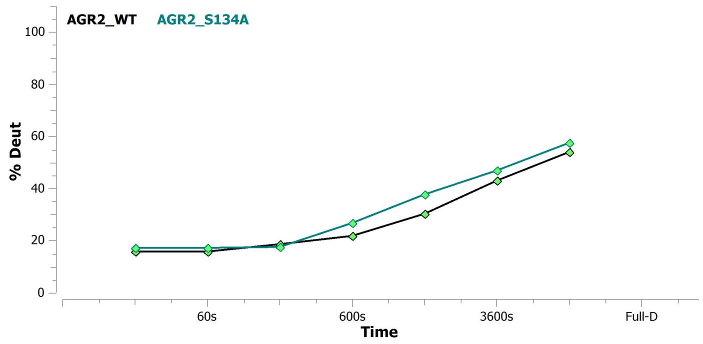

102-111: NKEIQKLAEQ

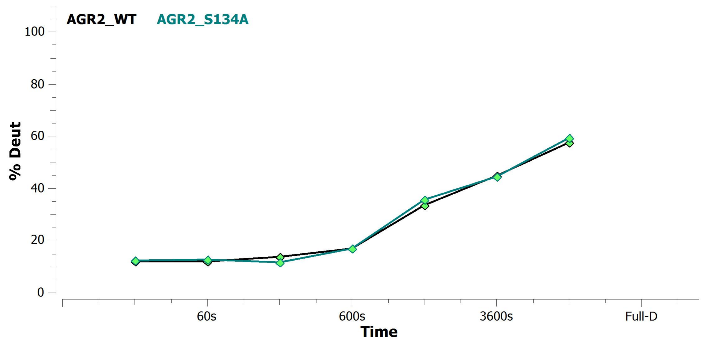

103-111: KEIQKLAEQ

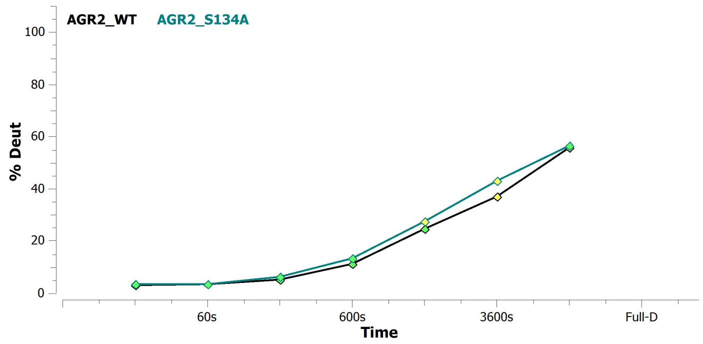

103-112: KEIQKLAEQF

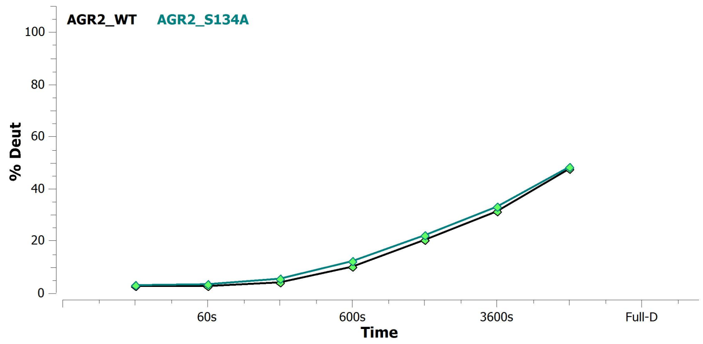

105-111: IQKLAEQ

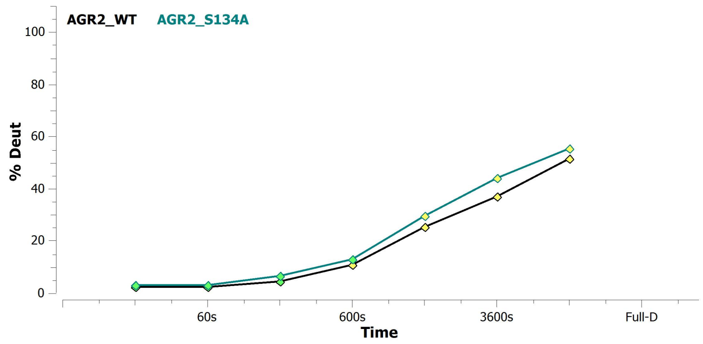

105-112: IQKLAEQF

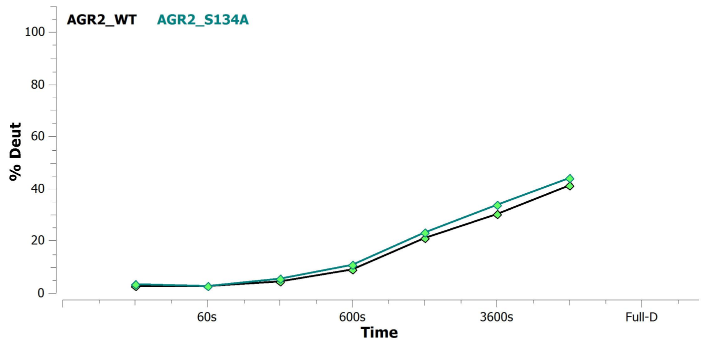

116-137: NLVYETTDKHLSPDGQYVPRIM

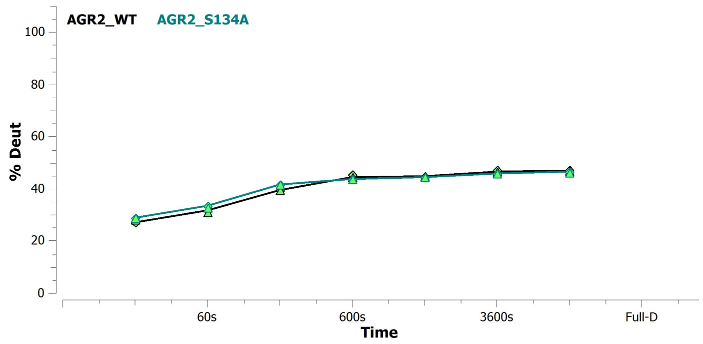

116-138: NLVYETTDKHLSPDGQYVPRIMF

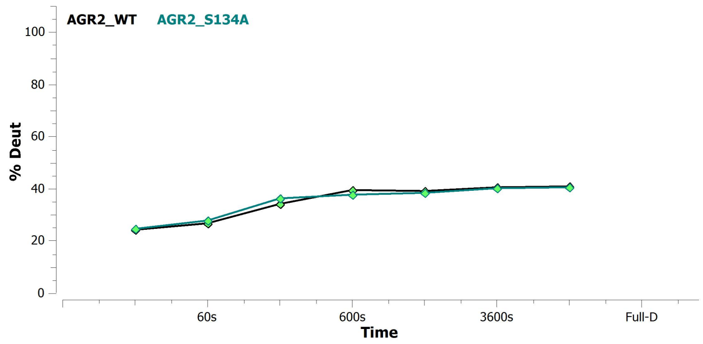

117-138: LVYETTDKHLSPDGQYVPRIMF

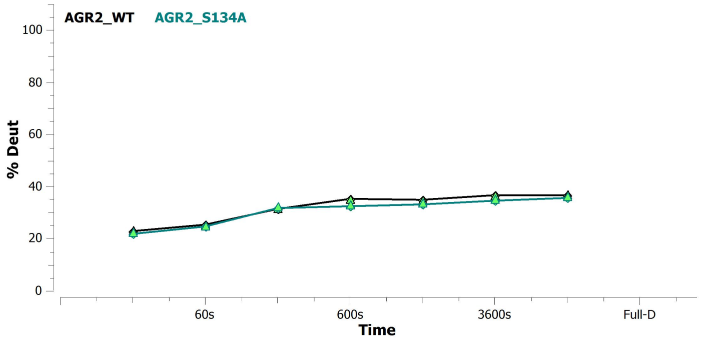

118-131: VYETTDKHLSPDGQ

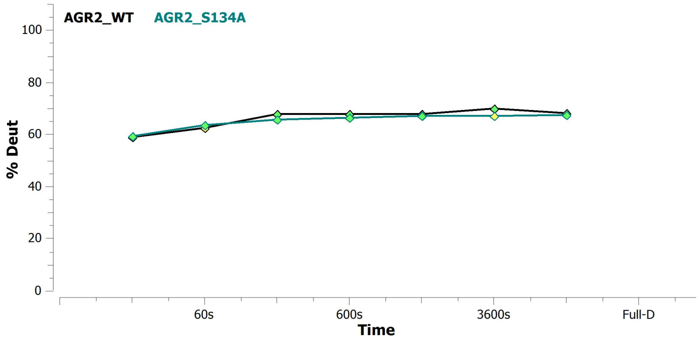

118-133: VYETTDKHLSPDGQYV

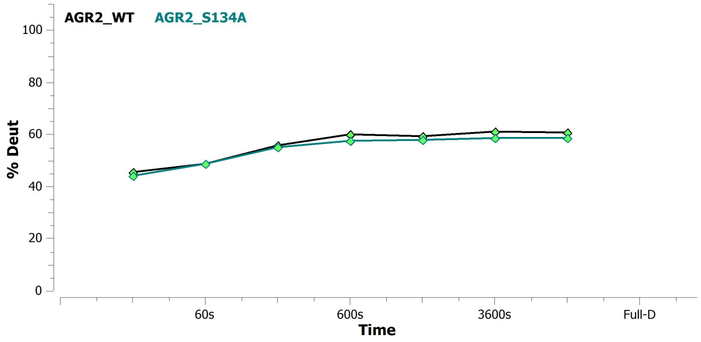

118-137: VYETTDKHLSPDGQYVPRIM

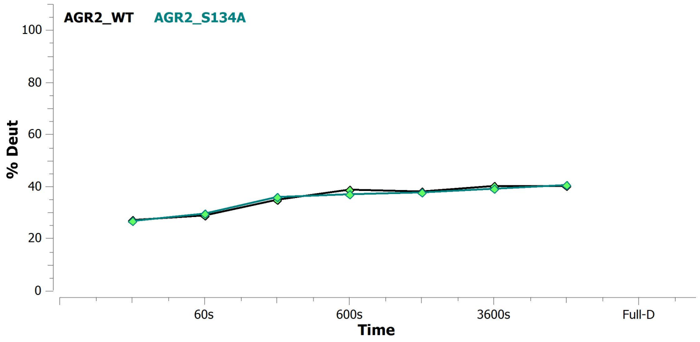

118-138: VYETTDKHLSPDGQYVPRIMF

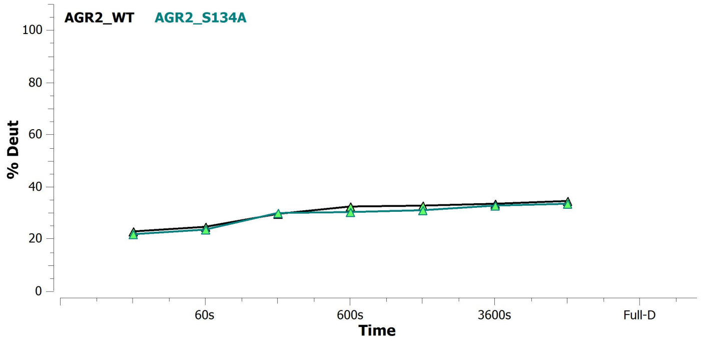

118-140: VYETTDKHLSPDGQYVPRIMFVD

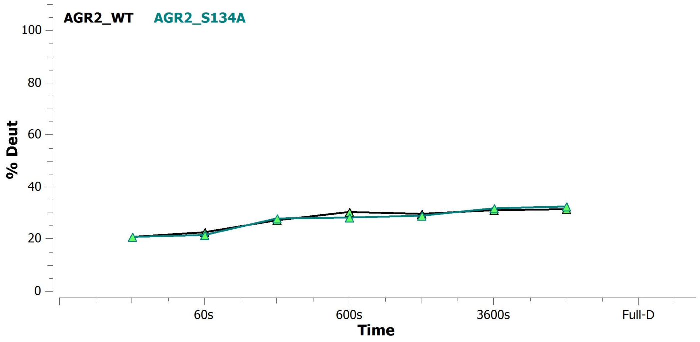

118-143: VYETTDKHLSPDGQYVPRIMFVDPSL

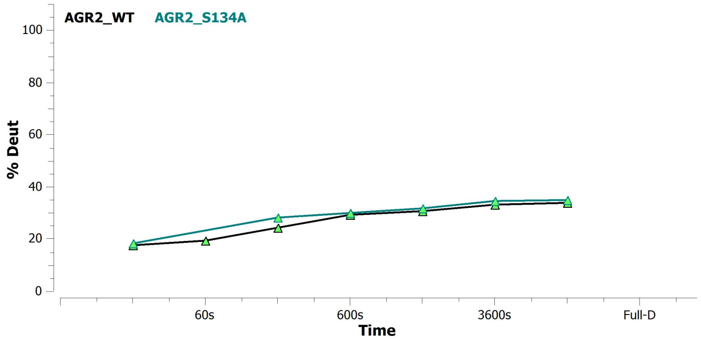

118-144: VYETTDKHLSPDGQYVPRIMFVDPSLT

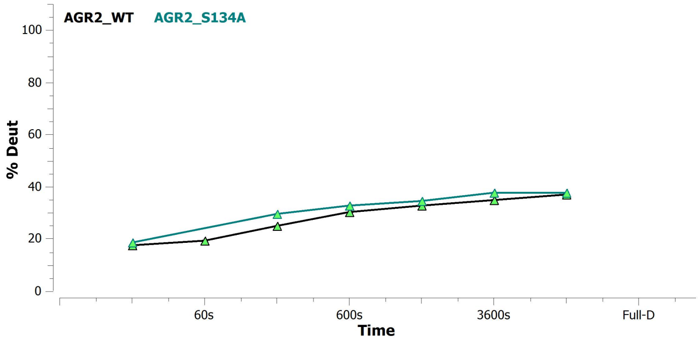

118-145: VYETTDKHLSPDGQYVPRIMFVDPSLTV

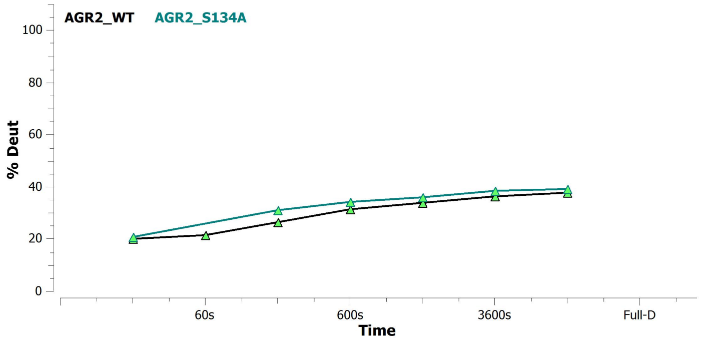

118-147: VYETTDKHLSPDGQYVPRIMFVDPSLTVRA

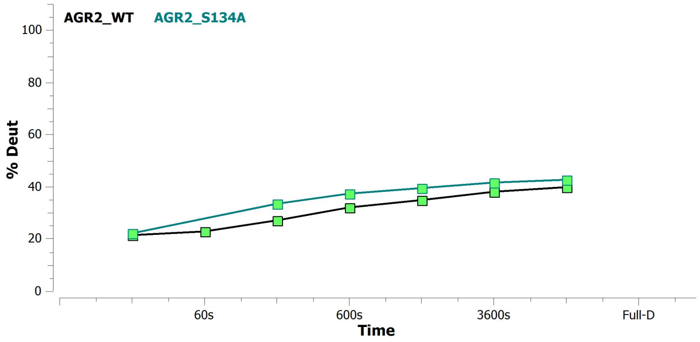

119-138: YETTDKHLSPDGQYVPRIMF

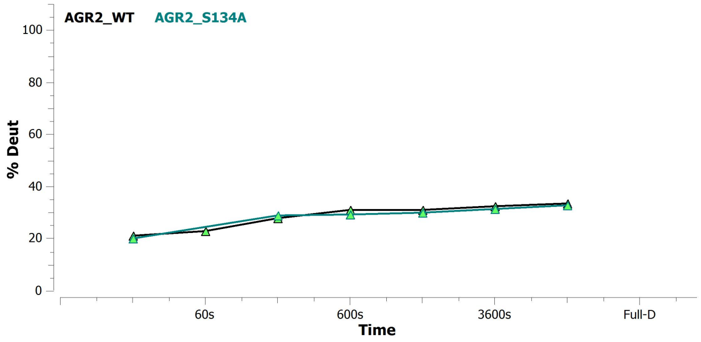

120-138: ETTDKHLSPDGQYVPRIMF

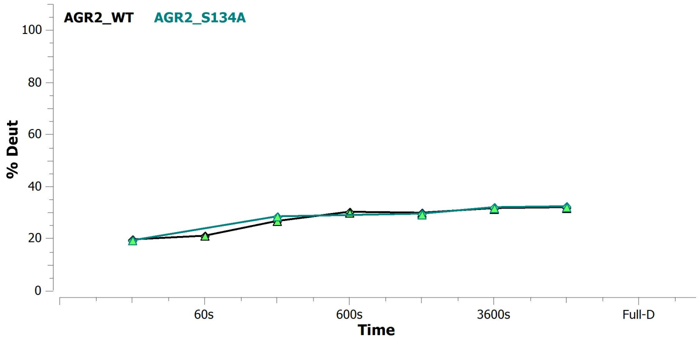

121-138: TTDKHLSPDGQYVPRIMF

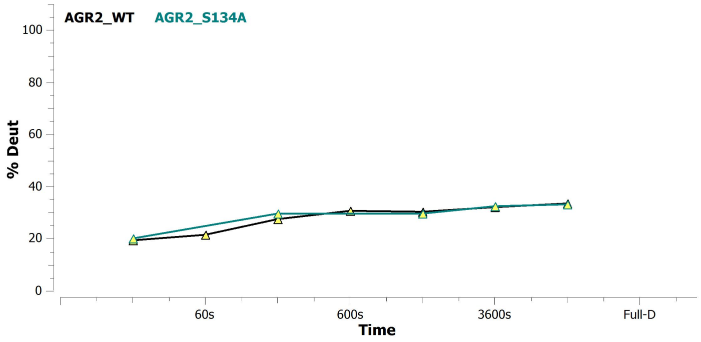

123-138: DKHLSPDGQYVPRIMF

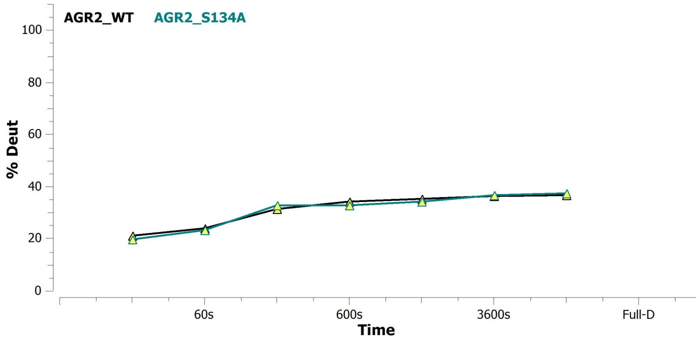

131-138: QYVPRIMF

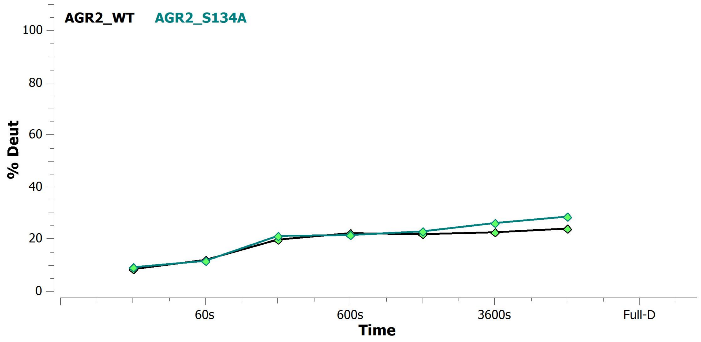

132-138: YVPRIMF

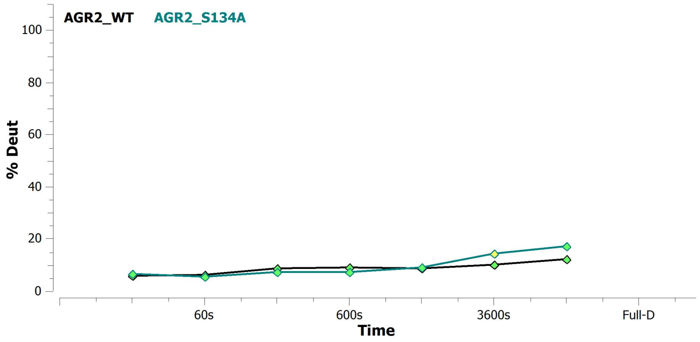

139-147: VDPSLTVRA

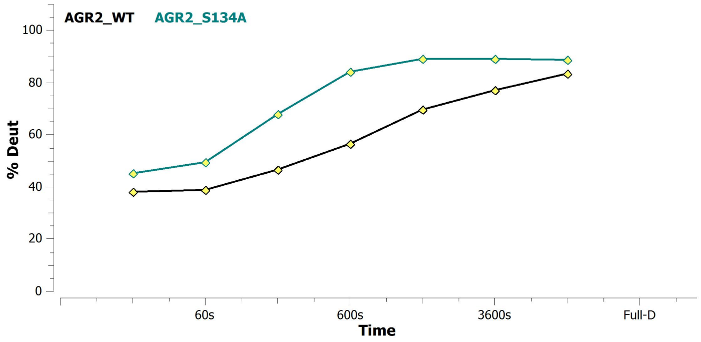

144-153: TVRADITGRY

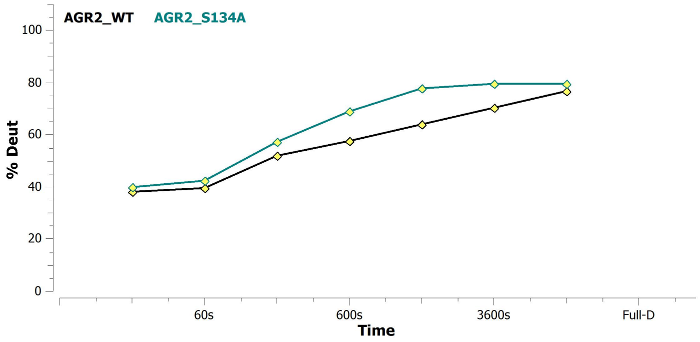

144-157: TVRADITGRYSNRL

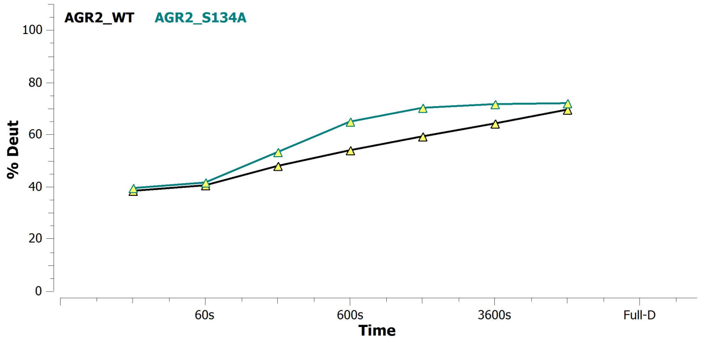

145-157: VRADITGRYSNRL

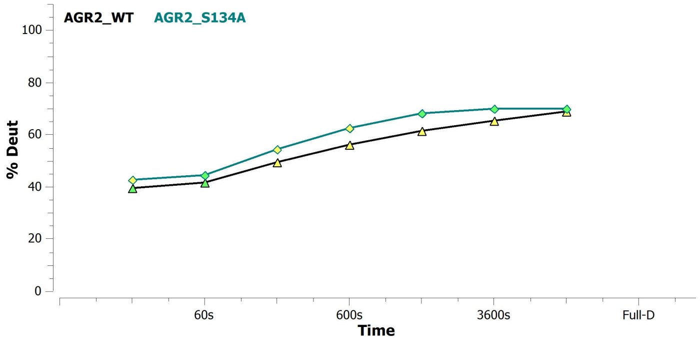

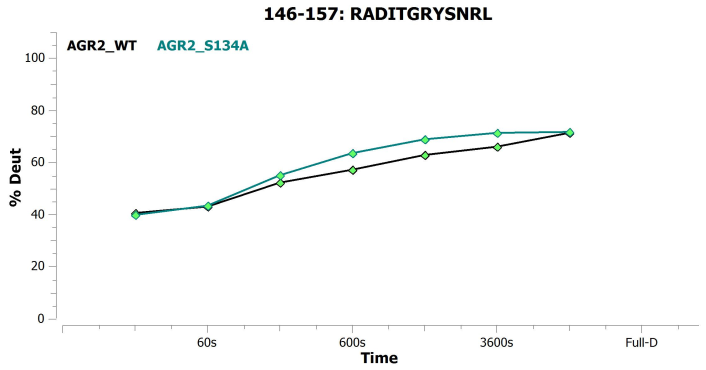
146-157: RADITGRYSNRL
AGR2_WT
AGR2_S134A
% Deut
Time
0
20
40
60
80
100
60s
600s
3600s
Full-D

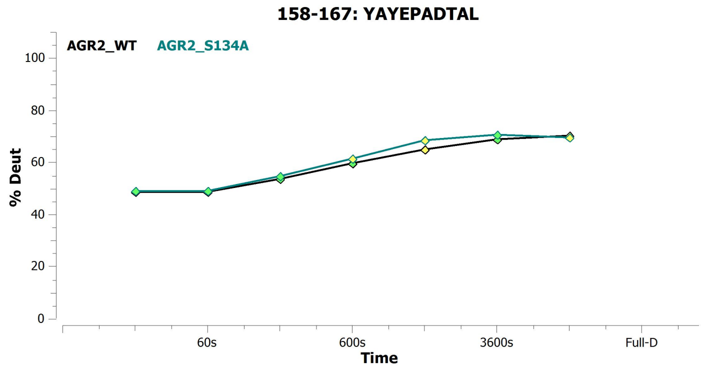
158-167: YAYEPADTAL
AGR2_WT
AGR2_S134A
% Deut
Time
0
20
40
60
80
100
60s
600s
3600s
Full-D

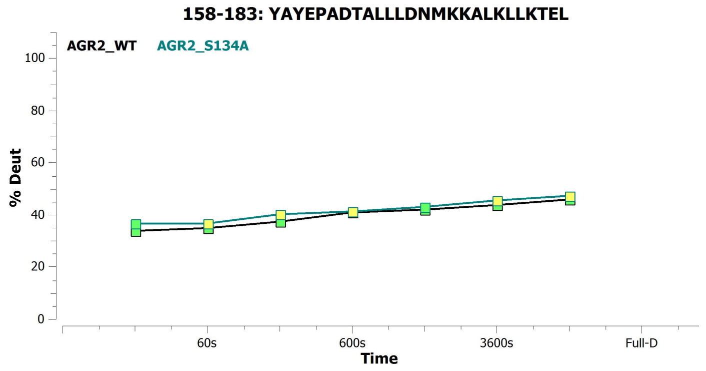
158-183: YAYEPADTALLLDNMKKALKLLKTEL
AGR2_WT
AGR2_S134A
% Deut
Time
0
20
40
60
80
100
60s
600s
3600s
Full-D

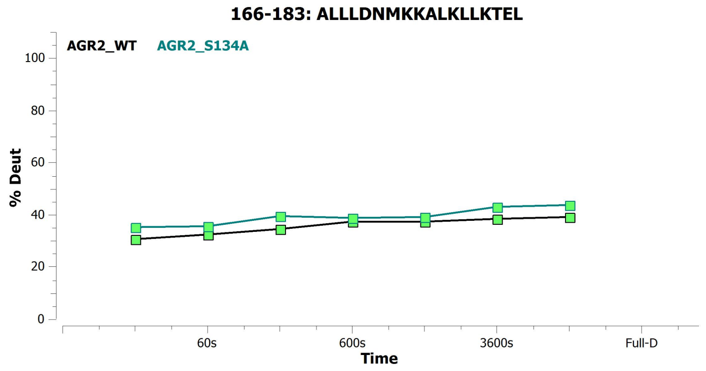
166-183: ALLLDNMKKALKLLKTEL
AGR2_WT
AGR2_S134A
% Deut
Time
0
20
40
60
80
100
60s
600s
3600s
Full-D

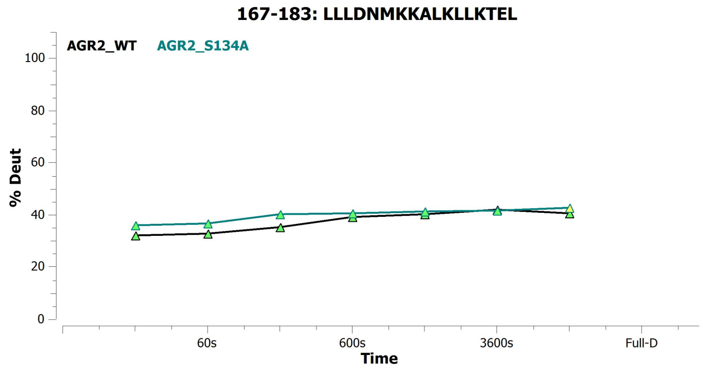
167-183: LLLDNMKKALKLLKTEL
AGR2_WT
AGR2_S134A
% Deut
Time
0
20
40
60
80
100
60s
600s
3600s
Full-D

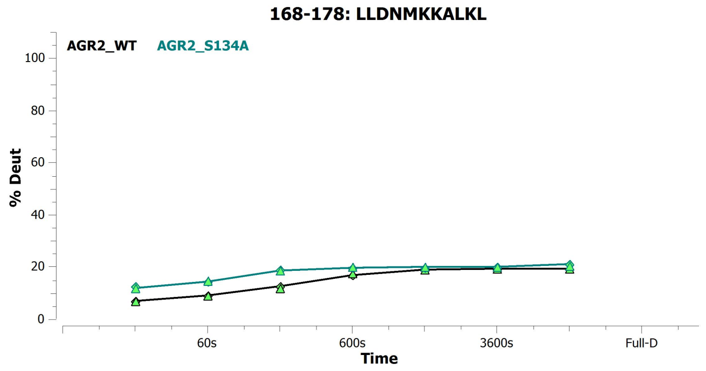
168-178: LLDNMKKALKL
AGR2_WT
AGR2_S134A
% Deut
Time
0
20
40
60
80
100
60s
600s
3600s
Full-D

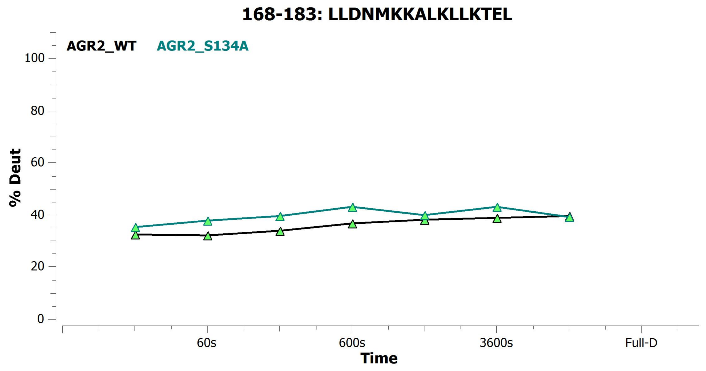
168-183: LLDNMKKALKLLKTEL
AGR2_WT
AGR2_S134A
% Deut
Time
0
20
40
60
80
100
60s
600s
3600s
Full-D

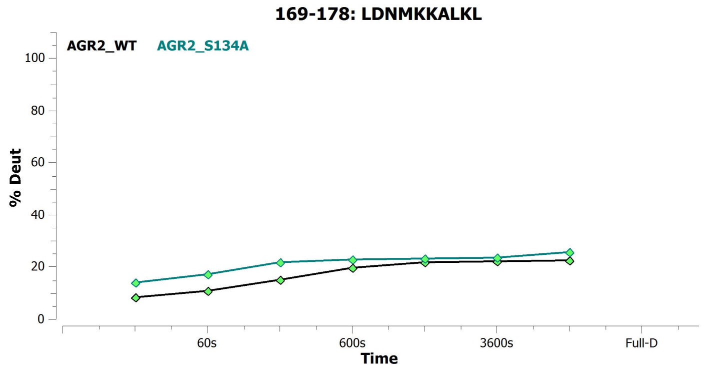
169-178: LDNMKKALKL
AGR2_WT
AGR2_S134A
% Deut
Time
0
20
40
60
80
100
60s
600s
3600s
Full-D

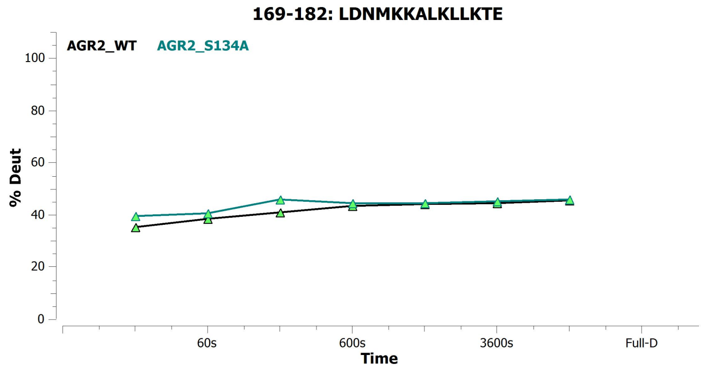
169-182: LDNMKKALKLLKTE
AGR2_WT
AGR2_S134A
% Deut
Time
0
20
40
60
80
100
60s
600s
3600s
Full-D

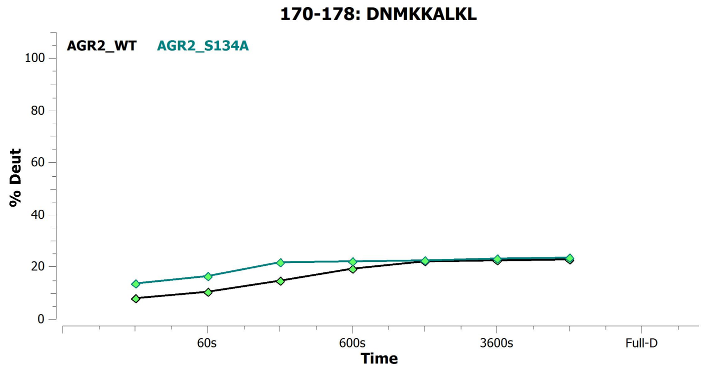
170-178: DNMKKALKL
AGR2_WT
AGR2_S134A
% Deut
Time
0
20
40
60
80
100
60s
600s
3600s
Full-D

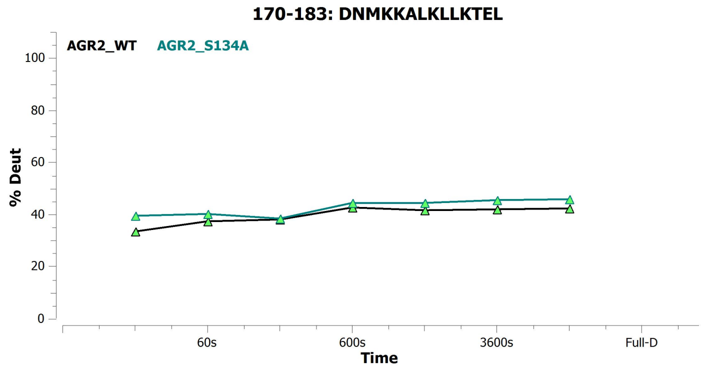
170-183: DNMKKALKLLKTEL
AGR2_WT
AGR2_S134A
% Deut
100
80
60
40
20
0
60s
600s
3600s
Full-D
Time

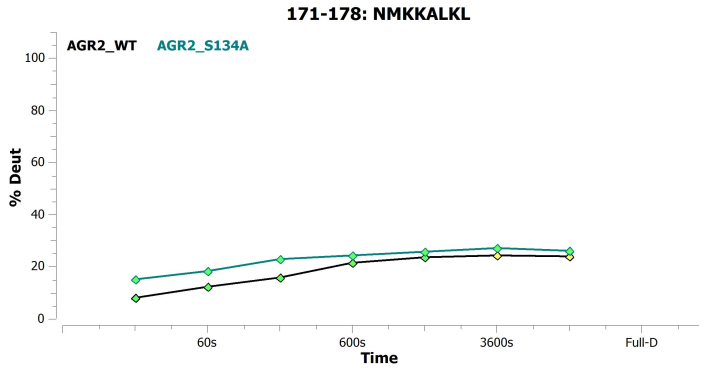
171-178: NMKKALKL
AGR2_WT
AGR2_S134A
% Deut
100
80
60
40
20
0
60s
600s
3600s
Full-D
Time

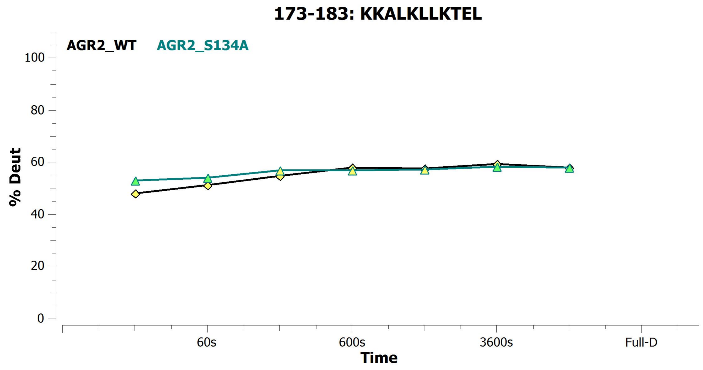
173-183: KKALKLLKTEL
AGR2_WT
AGR2_S134A
% Deut
100
80
60
40
20
0
60s
600s
3600s
Full-D
Time
