# Supplementary material for: The Sequence-specific Peptide-binding Activity of the Protein Sulfide Isomerase AGR2 Directs Its Stable Binding to the Oncogenic Receptor EpCAM
Source: Mol Cell Proteomics. 2018 Jan 16;17(4):737–63. doi: 10.1074/mcp.RA118.000573 (PMC5880107; doi:10.1074/mcp.RA118.000573)
Supplement: Supplemental Data [file supp_RA118.000573_134890_0_supp_50203_p258tz.pptx]

## Slide 1
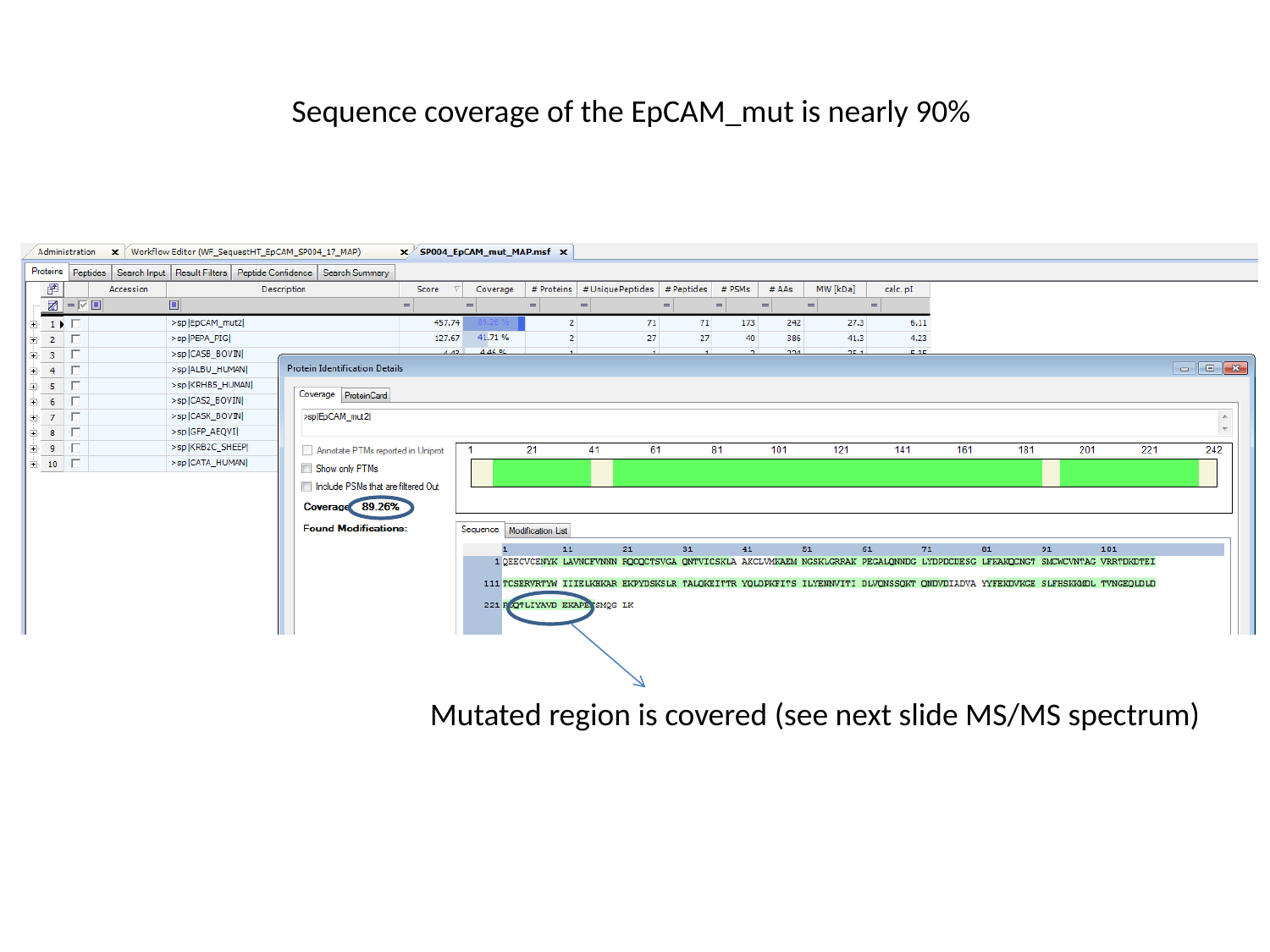

Sequence coverage of the EpCAM_mut is nearly 90%
Mutated region is covered (see next slide MS/MS spectrum)

## Slide 2
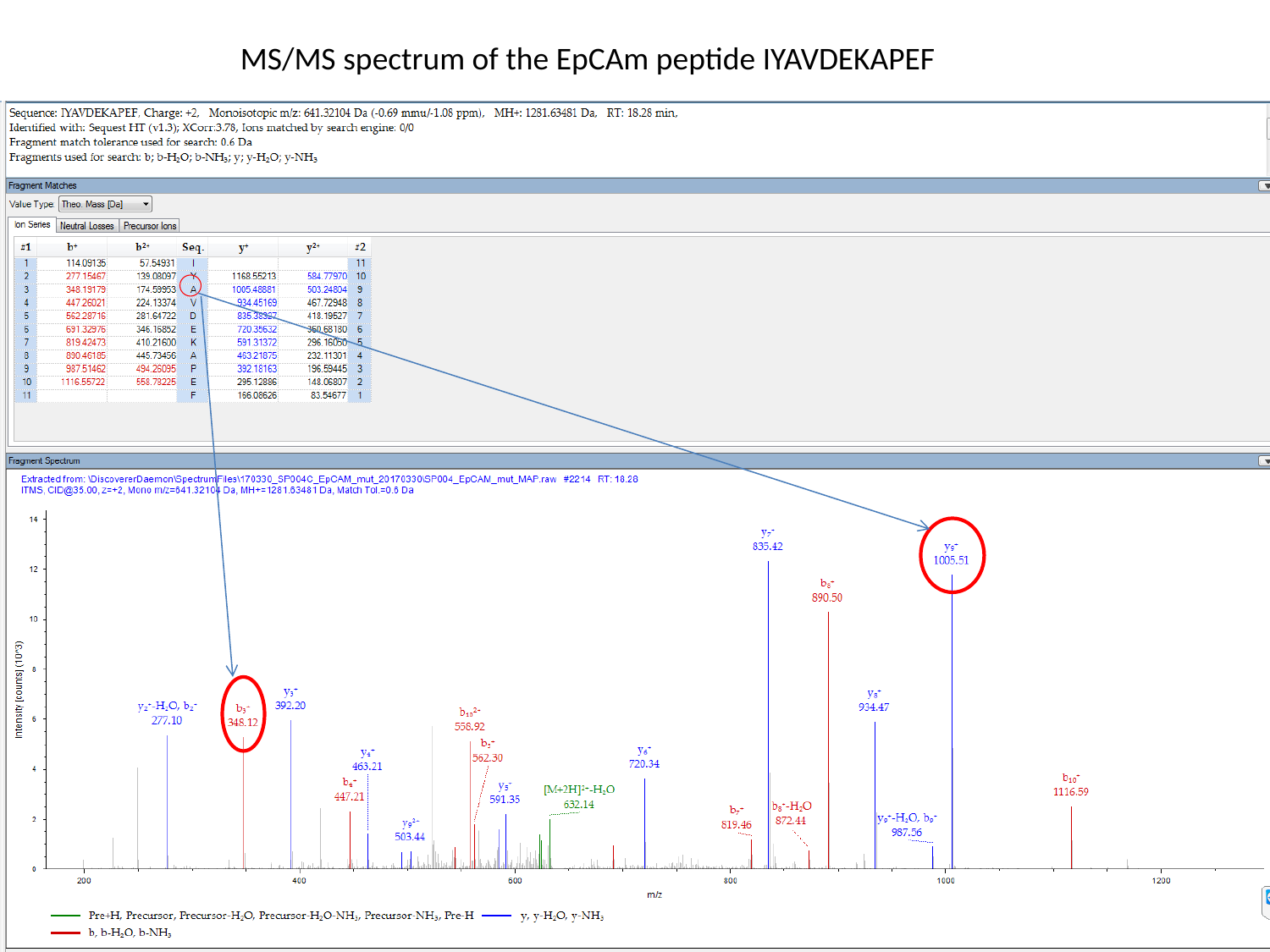

MS/MS spectrum of the EpCAm peptide IYAVDEKAPEF
