# Supplementary material for: The Sequence-specific Peptide-binding Activity of the Protein Sulfide Isomerase AGR2 Directs Its Stable Binding to the Oncogenic Receptor EpCAM
Source: Mol Cell Proteomics. 2018 Jan 16;17(4):737–63. doi: 10.1074/mcp.RA118.000573 (PMC5880107; doi:10.1074/mcp.RA118.000573)
Supplement: Supplemental Data [file supp_RA118.000573_134890_0_supp_51414_p2zdzt.docx]

**SUPPLEMENTARY Table 1**

1. Excel file of AGR2 linear peptide motif hits from *ScanProsite* database mining.

**SUPPLEMENTARY Table 2**

1. Excel file of functional enrichment analysis using *FunRich*.

**SUPPLEMENTARY Table 3**

1. Excel file of AGR2 linear peptide motif hits from *SLIMSEARCH4* database mining

**SUPPLEMENTARY Figures 1-10.**

1. Datasets summarizing MDM2 ligand binding effects on MDM2 conformation analyzed by hydrogen-deuterium exchange mass spectrometry.
2. Butterfly plots showing the effect of Nutlin-3 ligand on overall MDM2 deuteration changes
3. Datasets summarizing AGR2 ligand binding effects on AGR2 conformation analyzed by hydrogen-deuterium exchange mass spectrometry.
4. Butterfly plots showing the effect of peptide ligand on overall AGR2 deuteration changes
5. Datasets summarizing effects of the S134A mutation on the overall conformation of AGR2 protein analyzed by hydrogen-deuterium exchange mass spectrometry.
6. Butterfly plots showing the effect of S134A mutation on overall AGR2 deuteration changes
7. Datasets summarizing the deuteration of EpCAM protein (wt and mutant) with buffer only or with AGR2 protein.
8. Butterfly plots showing the effect of Y251A EpCAM mutation on overall deuteration changes in the presence of AGR2
9. Peptic coverage of both wt-EpCAM and Y251A-mutant EpCAM used for HDX analysis
10. Sequence coverage and MS/MS spectrum of Y251A EpCAM mutant to demonstrate that the mutated EpCAM protein can be detected in the HDX reaction (Figure 14C).
